# Exploring secondary-sphere interactions in Fe–N_*x*_H_*y*_ complexes relevant to N_2_ fixation†
†Electronic supplementary information (ESI) available: Synthetic procedures, spectroscopic data, reactivity studies, computational studies, crystallographic information. CCDC 1511362 (**1′**), 1511363 (**2′**), 1511369 (**3**), 1511371 (**3′**), 1511365 (**4**), 1511368 (**5**), 1511367 (**6**), 1511364 (**6′**), 1511366 and 1511370 contain supplementary crystallographic data for this paper. For ESI and crystallographic data in CIF or other electronic format see DOI: 10.1039/c6sc04805f



**DOI:** 10.1039/c6sc04805f

**Published:** 2016-12-08

**Authors:** Sidney E. Creutz, Jonas C. Peters

**Affiliations:** a California Institute of Technology , Division , of Chemistry and Chemical Engineering , Pasadena , California 91125 , USA . Email: jpeters@caltech.edu

## Abstract

Hydrogen bonding and other types of secondary-sphere interactions are ubiquitous in metalloenzyme active sites and are critical to the transformations they mediate. Exploiting secondary sphere interactions in synthetic catalysts to study the role(s) they might play in biological systems, and to develop increasingly efficient catalysts, is an important challenge. Whereas model studies in this broad context are increasingly abundant, as yet there has been relatively little progress in the area of synthetic catalysts for nitrogen fixation that incorporate secondary sphere design elements. Herein we present our first study of Fe–N_*x*_H_*y*_ complexes supported by new tris(phosphine)silyl ligands, abbreviated as [SiPNMe3] and [SiPiPr2P^NMe^], that incorporate remote tertiary amine hydrogen-bond acceptors within a tertiary phosphine/amine 6-membered ring. These remote amine sites facilitate hydrogen-bonding interactions *via* a boat conformation of the 6-membered ring when certain nitrogenous substrates (*e.g.*, NH_3_ and N_2_H_4_) are coordinated to the apical site of a trigonal bipyramidal iron complex, and adopt a chair conformation when no H-bonding is possible (*e.g.*, N_2_). Countercation binding at the cyclic amine is also observed for anionic {Fe–N_2_}^–^ complexes. Reactivity studies in the presence of proton/electron sources show that the incorporated amine functionality leads to rapid generation of catalytically inactive Fe–H species, thereby substantiating a hydride termination pathway that we have previously proposed deactivates catalysts of the type [EPR3]FeN_2_ (E = Si, C).

## Abstract 2

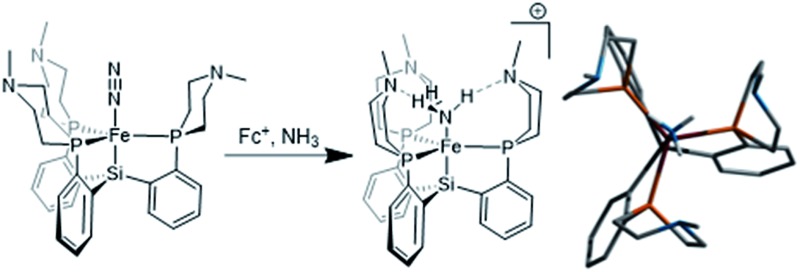
Pendant amines on a scaffold for Fe–N_2_ chemistry form hydrogen bonds with N_*x*_H_*y*_ substrates and facilitate proton delivery to Fe.

## Introduction

Whereas there has been sustained interest in modeling the structures and functions of metalloenzymes using synthetic, small-molecule systems,^1,2^ models of metalloenzyme active sites that reproduce only the primary coordination sphere rarely capture the catalytic activity of interest. In the vast majority of cases, model complexes lack important peripheral secondary sphere interactions commonly present in biological systems. Structural, computational, and mechanistic studies on metalloenzymes indicate that critical secondary sphere interactions, such as hydrogen bonding, commonly facilitate the enzyme's catalytic activity, mediated at one or more active site metal centers; these interactions can both stabilize intermediates and orchestrate the necessary arrangement of reactants.^3^

Synthetic inorganic chemists have increasingly taken up the challenge of preparing coordination complexes that incorporate secondary sphere interactions, especially *via* hydrogen bonding, with the hope of realizing both structurally and functionally faithful models of metalloenzyme active sites. In a number of cases, especially in the context of proton reduction and oxygen activation, these approaches have been notably successful, both with respect to engendering favorable catalytic reactivity and in stabilizing reactive species such as terminal metal oxos.^2^

A role for secondary sphere interactions in nitrogen fixation is less well established. Biological nitrogen fixation is a fascinatingly complex process that is catalyzed by several iron-containing active sites, of which the most well-studied is the Fe_7_MoS_9_C cluster in the iron–molybdenum cofactor (FeMoco). There is great interest in understanding the mechanism(s) by which nitrogenases mediate nitrogen fixation, but much uncertainty remains, including the potential presence or importance of hydrogen bonding interactions near the active site.^4–7^

**Fig. 1:**
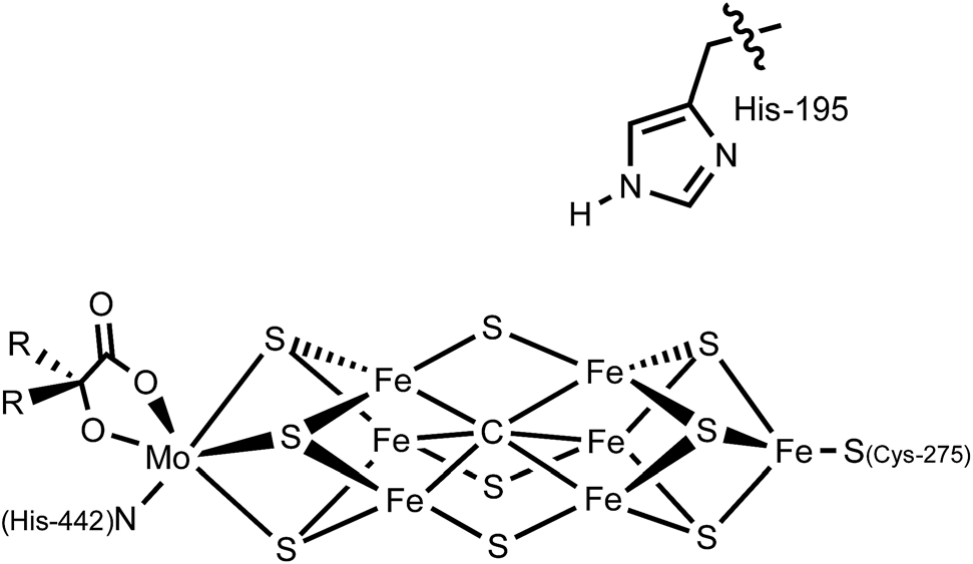
Schematic of the nitrogenase FeMo cofactor; His-195 may interact *via* hydrogen-bonding with the active site.

In the nitrogenase enzyme of *Azotobacter vinelandii*, a highly conserved histidine residue (His-195) is poised above the central irons (Fe_2_ and Fe_6_) on one face of the cluster. Based on computations and biological studies of mutant enzymes, it has been suggested that one or more of these iron centers may be the binding site for N_2_ and the locus of catalytic reduction to ammonia (Fig. 1).^4,5^ Removing this histidine residue *via* site-directed mutagenesis shuts down N_2_ fixation,^8^ suggesting that it may participate in the reaction, perhaps *via* hydrogen bonding or by acting as a proton shuttle to the active site. A recent crystal structure of a CO-bound form of FeMoco appears consistent with the presence of a hydrogen bond between the terminal oxygen atom of a bridging CO ligand and the N–H moiety of this histidine residue.^9^

**Fig. 2:**
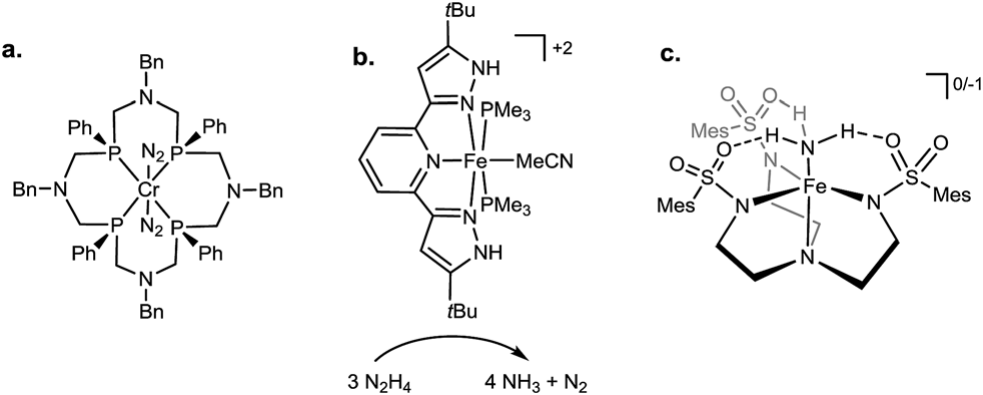
Select previously reported systems incorporating hydrogen-bonding or proton-responsive ligands for the binding and/or conversion of nitrogenous substrates. (a) Treatment of a Cr(0)–(N_2_)_2_ complex within a scaffold bearing tertiary amines with acid produces ammonia and hydrazine.^13*a*^ (b) The participation of a proton-responsive ligand is invoked in the disproportionation of hydrazine by Fe(ii).^14^ (c) Fe(ii) and Fe(iii) complexes of ammonia show multiple hydrogen-bonding interactions with ligand.^16^

While there has been considerable effort invested in the development of functional inorganic Mo and Fe systems as limiting models of biological nitrogen fixation,^10,11^ there has been correspondingly less investment of effort towards incorporating secondary sphere interactions into such models. There are nonetheless several studies that have probed for secondary sphere interactions in M–N_*x*_H_*y*_ species (Fig. 2). For instance, within group 6, the influence of pendant amine groups on the protonation of Mo– and W–N_2_ complexes was examined,^12^ and Cr–N_2_ complexes supported by ligands featuring pendant tertiary amines were shown to undergo protonation to liberate some NH_3_ (Fig. 2a);^13^ it remains as yet unclear what role, if any, these amines play in the N_2_ protonation in this Cr system. In relation to Fe–N_*x*_H_*y*_ systems, secondary sphere participation has been highlighted in an iron catalyst for hydrazine disproportionation; ligand –NH groups were proposed to aid in the delivery of protons to the substrate (Fig. 2b).^14^ Recent work from our laboratory furnished a structurally characterized Fe=NNH_2_^+^ species derived from protonation of Fe–N_2_; this reactive species was stabilized in the solid state by hydrogen bonding interactions to exogenous anion and ethereal solvent.^15^ Finally, Fe(ii)–NH_3_ and Fe(iii)–NH_3_ complexes bearing secondary-sphere hydrogen-bonding interactions were recently characterized by the Borovik group (Fig. 2c).^16^ Examples of secondary sphere interactions in M–N_*x*_H_*y*_ species are known for other transition metals as well.^17^

Our lab has extensively studied a series of tetradentate trisphosphine ligands with apical N, Si, B, or C donors in the context of N_2_ activation and reduction, including catalytic N_2_-to-NH_3_ conversion.^11a,b,18^ We sought to redesign these ligands to enable the examination of intramolecular secondary sphere H-bonding interactions within Fe–N_*x*_H_*y*_ species in systems that mediate N_2_-to-NH_3_ conversion, and to probe the resulting consequences on reactivity.

Towards this end we report here our first foray in this effort *via* the synthesis of tetradentate tris(phosphine)silyl ligands that incorporate tertiary amines as hydrogen bond acceptors. We have successfully elucidated the presence of intramolecular hydrogen-bonding interactions with several Fe–N_*x*_H_*y*_ species of relevance to catalytic N_2_ reduction. Additionally, we have shown that the presence of the pendant tertiary amine dramatically alters the outcome of Fe–N_2_ protonation reactions in these scaffolds.

## Results and discussion

### Ligand design and synthesis

We targeted modification of the [SiP_3_]Fe system we have studied previously (SiP_3_ = tris(phosphine)silyl)^18^ by inclusion of tertiary amines within the phosphine donor groups as conduits of secondary-sphere functionality, anticipating the stability of such motifs to the presence of strong reductants and acids, reagents we have employed in Fe-mediated N_2_-to-NH_3_ conversion.^11^ Incorporation of the pendant amine groups within six-membered heterocyclic phosphine/amine rings was expected to provide sufficient rigidity to position the hydrogen-bonding groups around the substrate binding cavity while allowing sufficient flexibility for the ligand to adjust to the presence of different substrates in the metal binding pocket.

**Scheme 1:**
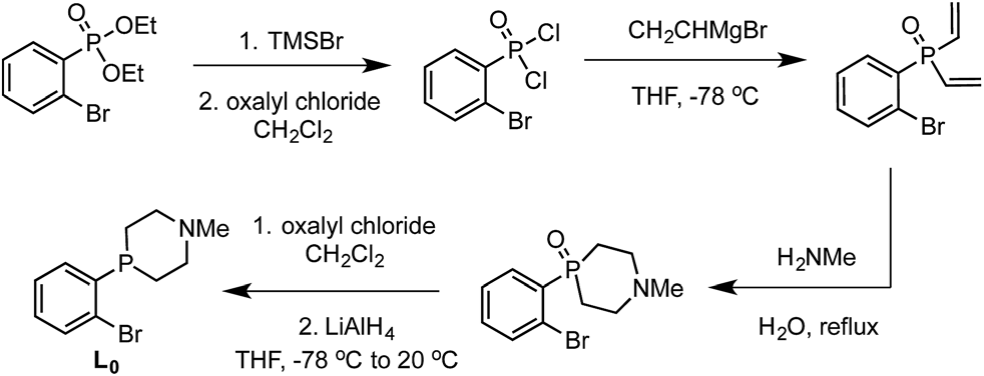
Synthesis of ligand arm **L_0_**.

**Scheme 2:**
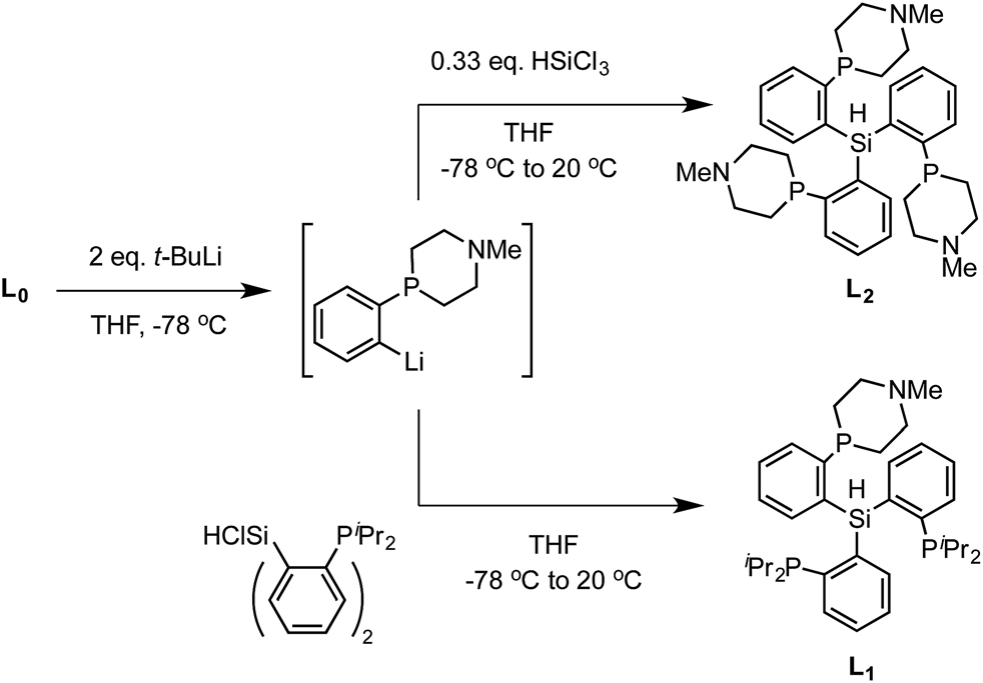
Synthesis of ligands **L_1_** and **L_2_**.

Synthesis of the required donor arm (**L_0_**) involved generating *o*-bromophenyldivinylphosphine oxide followed by cyclization with methylamine *via* a double Michael-type addition.^19^ The resulting phosphine oxide azacycle was then reduced to the desired phosphine (Scheme 1), followed by lithiation of the donor arm and addition to an appropriate electrophile; unsymmetric ([SiPiPr2P^NMe^]H, **L_1_**) and symmetric ([SiPNMe3]H, **L_2_**) tris(phosphine)silyl ligands were assembled in this fashion (Scheme 2).

### Synthesis of precursor iron complexes of **L_1_** and **L_2_**

**Scheme 3:**
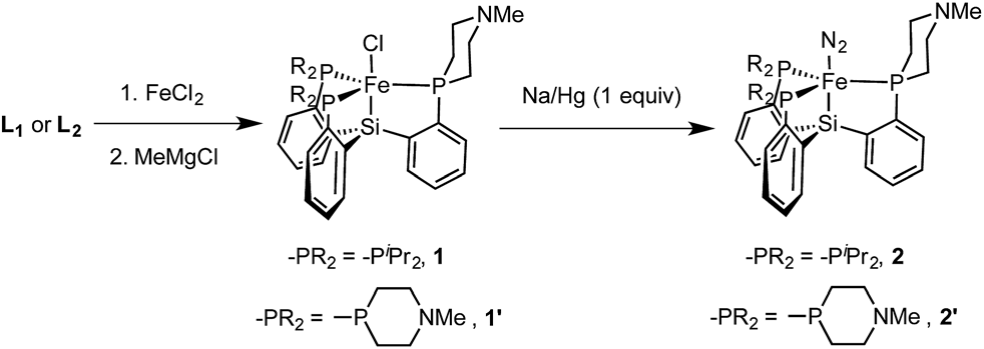
Synthesis of Fe(ii) and Fe(i) precursor complexes.

**Fig. 3:**
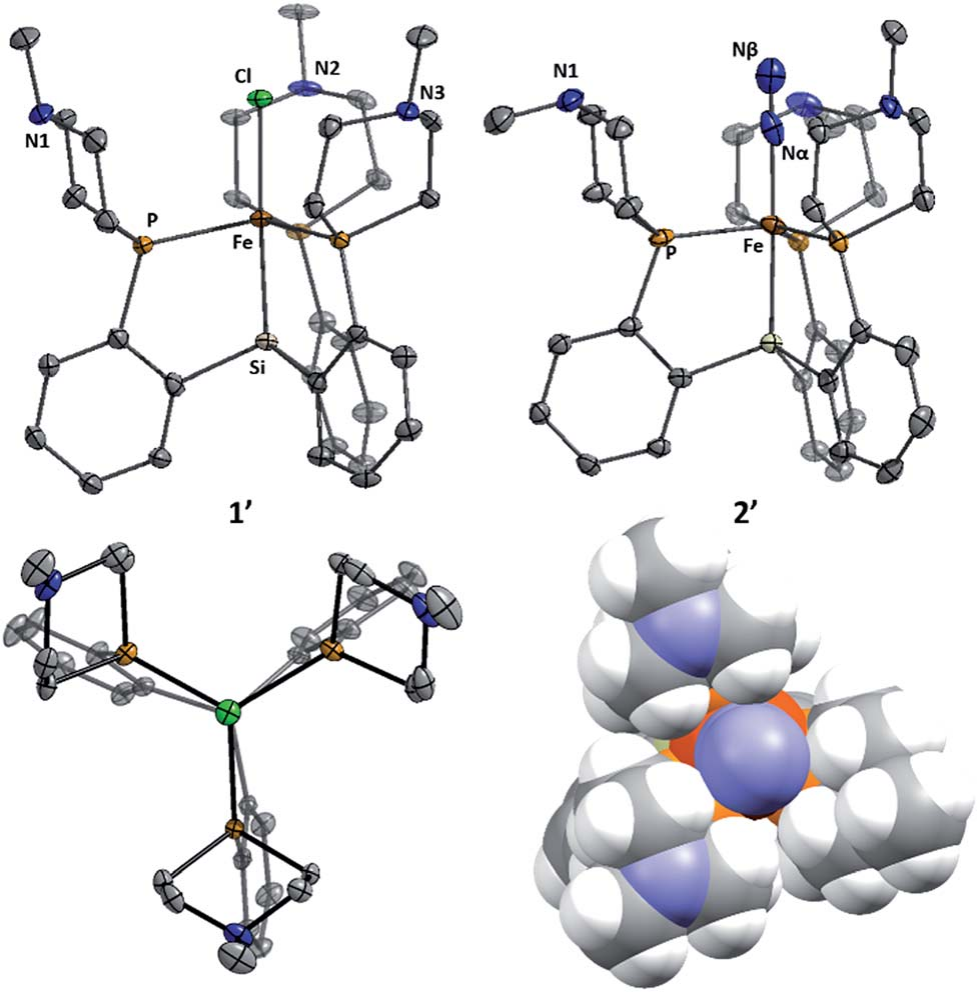
Structures of **1′** (left, two views) and **2′** (right, with space-filling view down N–N–Fe axis). Solvent molecules and hydrogen atoms omitted for clarity. Thermal ellipsoids are shown at 50% probability.

Metallation of the new ligands **L_1_** and **L_2_** on iron followed an approach similar to that used previously for the preparation of complexes of the parent ligand [SiPiPr3]H;^18b,c^ initial complexation of the phosphines **L_1_** or **L_2_** with FeCl_2_, followed by treatment with methylmagnesium chloride, putatively generates a transient Fe–Me species that loses methane *via* Si–H activation to afford trigonal bipyramidal Fe(ii) chloride complexes. Sodium amalgam (Na/Hg) reduction then generates the corresponding Fe(i) complexes with loss of sodium chloride and uptake of N_2_ (Scheme 3 and Fig. 3). Comparison of the spin states and IR parameters for these complexes to the parent [SiPiPr3]Fe system establishes that the electronic properties of the azacyclic phosphine donor are very similar to the parent donor—**2**, **2′**, and [SiPiPr3]FeN_2_ are all low-spin, *S* = 1/2 complexes with similar N–N vibrational frequencies (2005, 2007, and 2008 cm^–1^, respectively). This fact should facilitate interpretation of the effects of secondary sphere interactions on the properties of these respective complexes.

The azaphosphacycles in these complexes, in which no hydrogen bonding is present, adopt a chair conformation, with the N–Me group residing in either a pseudo-axial or pseudo-equatorial position. This is to be expected for a saturated six-membered ring,^20^ and in this conformation the tertiary amine is not well positioned for hydrogen bonding to an N_*x*_H_*y*_ substrate coordinated to iron in the axial site. As described below, hydrogen bonding requires the ring to adopt the energetically less favorable boat or twist-boat conformation. This feature provides a useful structural diagnostic for the presence of hydrogen bonding interactions.

### Generation of Fe–N_*x*_H_*y*_ complexes

We have synthesized and structurally characterized iron complexes coordinated by the N_*x*_H_*y*_ ligands NH_3_, N_2_H_4_, and NH_2_ using **L_1_** and **L_2_**. These compounds demonstrate the ability of these new auxiliary ligands to engage in hydrogen bonds with reduced N_*x*_H_*y*_ substrates.

**Scheme 4:**
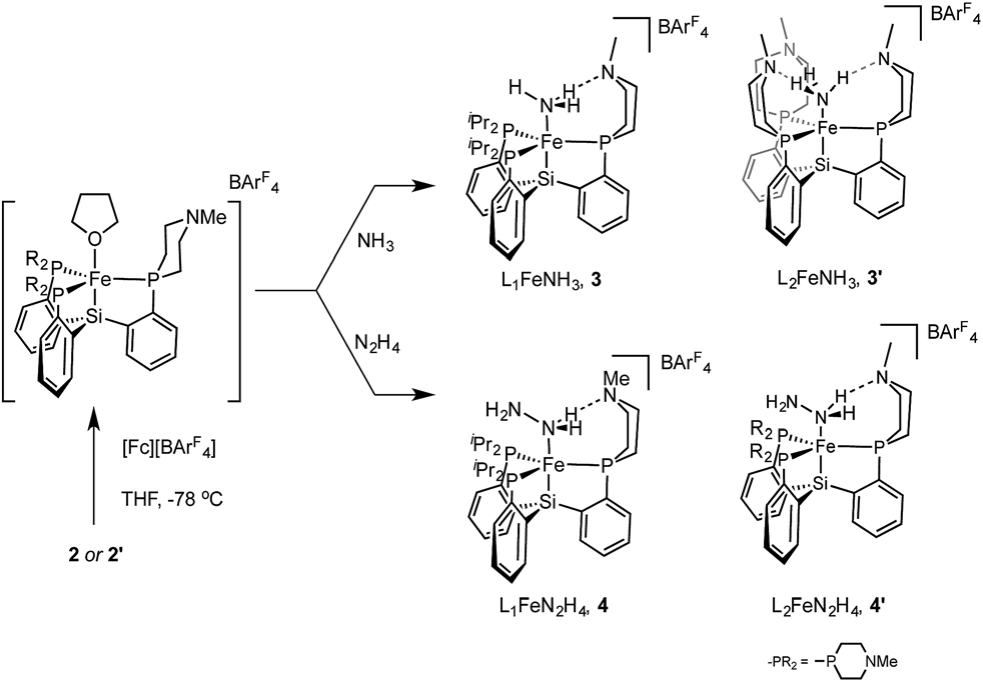
Synthesis of cationic NH_3_ and N_2_H_4_ adducts of LFe(ii).

Access to the hydrazine and ammonia adducts proceeds *via* oxidation of **2** or **2′** with [Fc][BArF4] (Ar^F^ = 3,5-bistrifluoromethylphenyl; Fc = ferrocenium) to generate *in situ* an **L_1_** [Fe(ii)] or **L_2_** [Fe(ii)] complex which is presumably solvated by THF (Scheme 4), followed by treatment with hydrazine or gaseous ammonia to afford the desired complex. The resulting Fe–NH_3_ adducts **3** and **3′**, and the Fe–N_2_H_4_ adduct **4** are stable to vacuum and are readily isolated in pure form; they have been crystallographically characterized. Complex **4′** is not sufficiently stable to be isolated (*vide infra*).

**Fig. 4:**
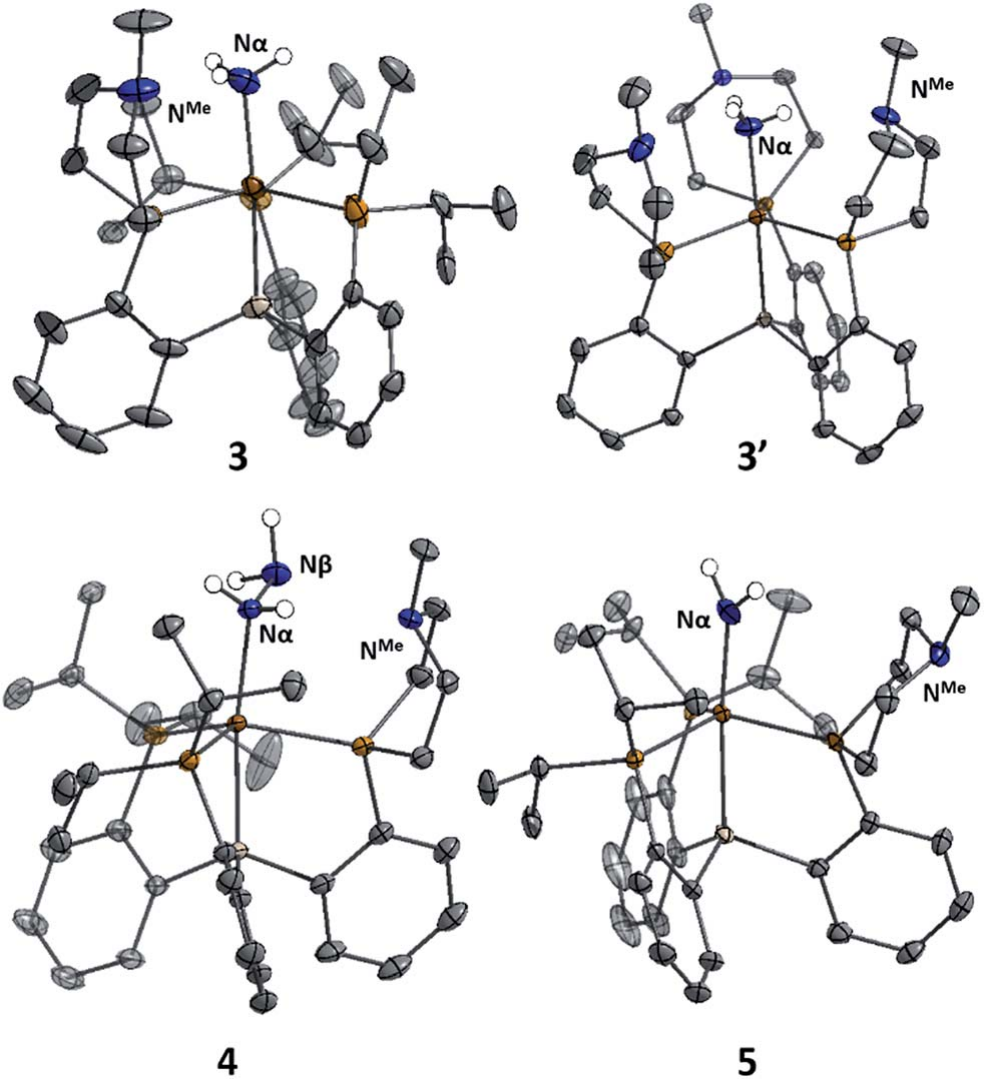
Structure of ammonia, hydrazine, and amide complexes **3**, **3′**, **4**, and **5**. BArF4 counteranions, solvent molecules, and carbon-bound hydrogen atoms have been omitted for clarity. Thermal ellipsoids are shown at 50% probability.

The X-ray structures of **3**, **3′**, and **4** are gratifying in that they clearly illustrate the presence of hydrogen bonding interactions between the tertiary amines of the tris(phosphine)silyl ligands and the N–H bonds of the respective Fe–N_*x*_H_*y*_ species (Fig. 4). The boat-type conformations of the six-membered azaphosphine rings in **3**, **3′**, and **4** are diagnostic for the presence of at least moderately strong hydrogen bonds; the energetic benefit of the hydrogen bonds must be sufficient to overcome the energetic penalty of adopting the disfavored boat (*vs.* chair) conformation (*vide infra*). Accordingly, chair conformations of the azaphosphine rings are instead observed in all structures we have examined that lack hydrogen bond interactions with an apically coordinated substrate.

In the structures of complexes **3**, **3′**, and **4**, the N-bound hydrogen atoms were located in the density difference map and their positions were allowed to refine freely. In all cases where hydrogen bonds were apparent from the conformation of the azaphosphacycles, hydrogen atoms were located in the expected positions, bound to the Fe-coordinated nitrogen atom and appropriately oriented for interaction with the tertiary amine acceptors. In the case of ammonia complex **3′**, two independent molecules were found to be present in the asymmetric unit. In one of these molecules all three ligand azaphosphacycles occupy a boat conformation indicative of hydrogen bonding interactions with the three ammonia hydrogen atoms; in the second molecule, two of the ligand arms are engaged in hydrogen bonding, while the third arm is disordered (3 : 2) between boat and chair conformations, perhaps suggestive of a weaker interaction. The shortest donor–acceptor bond lengths between N_α_ and N^Me^ are 2.994 Å, 2.984 Å, and 3.074 Å in **3**, **3′**, and **4**, respectively. The donor–acceptor distances for the hydrogen bonds are in the regime of what has been classified as a “moderate” strength hydrogen bond.^21^

Since engaging in hydrogen bonds with a coordinated substrate requires the six-membered rings of the ligand to adopt a higher-energy conformation (boat rather than chair), a lower limit for the strength of the hydrogen bonds can be obtained if the difference in energy between these two conformations is known. For cyclohexane, the twist-boat conformation lies approximately 5.5 kcal mol^–1^ higher in energy than the chair conformation.^20^ To approximate the energy difference for our system, DFT optimizations were carried out on the uncoordinated ligand **L_1_** with the azaphosphine ring in the chair (as in the structure of **1′** or **5**, *vide infra*) or boat (as in the structure of **3**) conformation, with ligand isopropyl groups truncated to methyls (see ESI† for details). The energy difference between the two conformers was calculated to be approximately 4.1 kcal mol^–1^; this value corresponds to the minimum stabilization energy imparted by each hydrogen bond, again consistent with a hydrogen bond of moderate strength.

Both **4** and **4′** are unstable in solution, decomposing to give the ammonia complexes **3** and **3′**, respectively. While **4** decomposes over the course of several hours at 60 °C, **4′** decomposes so quickly in solution that it has not been possible to isolate it in pure form; full conversion to **3′** is observed within several minutes at room temperature. The parent complex, {[SiPiPr3]FeN_2_H_4_}{BArF4}, is also susceptible to a similar decomposition process; however, in this case full conversion to the ammonia complex requires heating at 60 °C for several days (see ESI†). The kinetics of these decomposition reactions show complex behavior that we suspect are indicative of autocatalytic reactions, similar to that reported for the metalloboratrane complex {[TPBFe]N_2_H_4_}{BArF4}.^22^ The nature of the autocatalyst has not, however, been identified in either case and complicates quantitative kinetic analysis. Qualitatively, the rate of the decomposition reaction appears to be increased in the presence of hydrogen bond acceptors within the secondary coordination sphere. Similar observations have been made in another iron system that is capable of catalytically disproportionating hydrazine.^14^

**Scheme 5:**
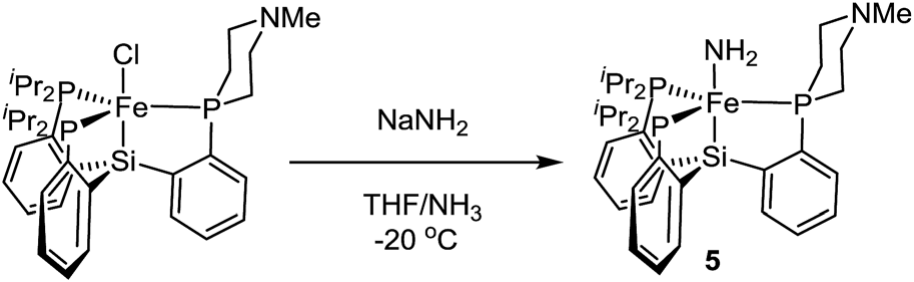
Synthesis of a parent amide complex (**5**).

Terminal parent amide (–NH_2_) complexes of iron are relatively rare despite their relevance as a possible late stage intermediate in Fe-mediated N_2_-to-NH_3_ conversion;^4^ only two examples of terminal Fe–NH_2_ species have been previously structurally characterised.^22,23^ The Fe(ii) amide complex **L_1_** Fe–NH_2_ (**5**) is readily synthesized by treating **L_1_** FeCl (**1**) with excess NaNH_2_ in 1 : 1 THF/NH_3_ solvent (Scheme 5). The identity of the –NH_2_ ligand was confirmed by digesting the complex with HCl and then analyzing the resulting solution for ammonia using the indophenol method;^24^ quantitative generation of ammonia was established. Complex **5** has been structurally characterized and does not show any hydrogen bonding to the ligand in the solid state (Fig. 4). Consistent with this, the azaphosphine ring adopts a chair conformation. The Fe–NH_2_ ligand in **5** is expected to be less acidic than the Fe–NH_3_^+^ ligand in the cationic complexes **3** and **3′**; further stabilization of the smaller degree of partial positive charge on the Fe–NH_2_ hydrogens *via* hydrogen bonding is hence not sufficiently favorable to overcome the energetic cost of conformationally flipping the azaphosphacycle to the boat conformation.

Oxidation of **5** with [Fc][BArF4] in Et_2_O in an attempt to generate **L_1_** Fe–NH_2_^+^ instead afforded the ammonia complex **3** as the primary detectable product. We had hoped that generation of **L_1_** Fe–NH_2_^+^ might afford a viable precursor to a terminal **L_1_** Fe(NH) species. However, the instability of **L_1_** Fe–NH_2_^+^ (or a corresponding **L_1_** Fe(NH) species in the event a proton is transferred to the cyclic amine) suggests that reaction with solvent (perhaps *via* HAT) and/or disproportionation is too rapid. Carrying out the reaction in thawing 2-MeTHF does allow for the detection of a new *S* = 1/2 species by EPR at 77 K (see ESI†), but this species decays upon warming. Attempts to trap or further characterize this intermediate have not yet been successful.

### Synthesis and reactivity of reduced Fe–N_2_ complexes

**Scheme 6:**
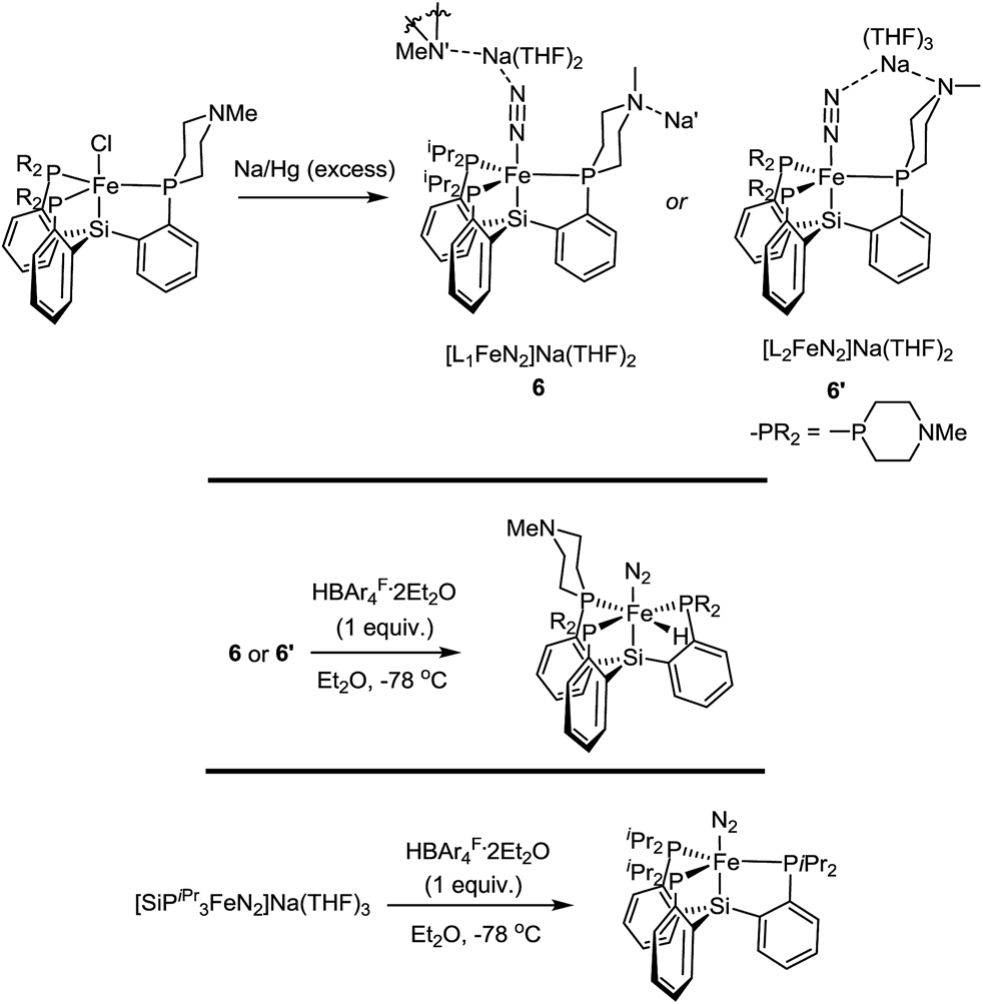
Synthesis of Fe(0)–N_2_ complexes, and their reaction profiles with HBArF4·2Et_2_O.

**Fig. 5:**
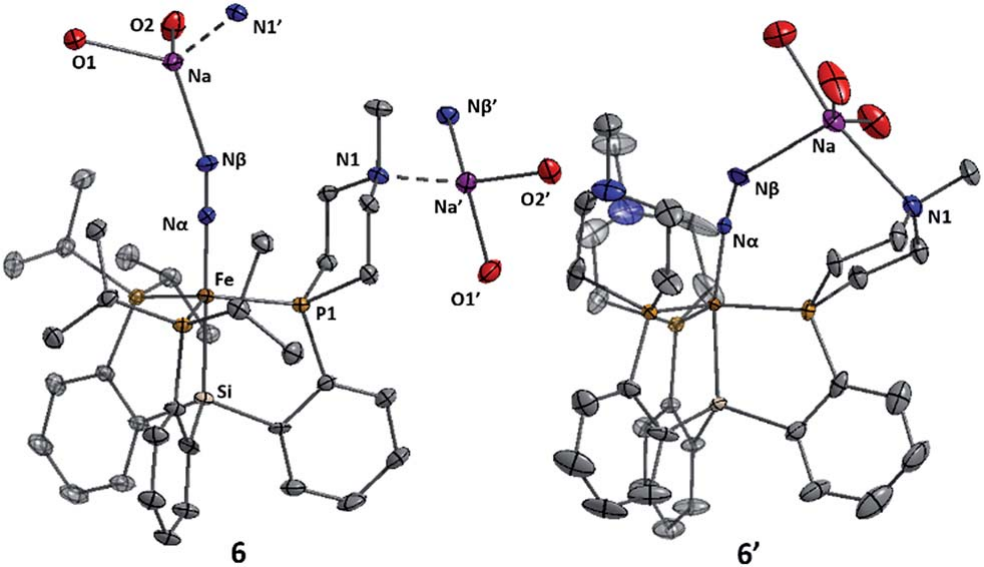
Structures of complexes **6** and **6′**. For **6**, the intermolecular interaction (dashed lines) between the sodium cation and the tertiary amine group of a neighboring molecule (N1′) is shown, as well as the interaction with the sodium countercation of another neighbor (Na′). Thermal ellipsoids are shown at 50% probability, and hydrogen atoms and uncoordinated solvent are omitted for clarity. Coordinated THF molecules are truncated to show only the oxygen atom bound to Na.

The Fe systems we have studied previously that generate significant or catalytic amounts of NH_3_ from N_2_ use anionic (pre)catalysts of the type L_*n*_FeN_2_^–^.^11^ Therefore, we synthesized and characterized **L_1_** FeN_2_^–^ and **L_2_** FeN_2_^–^ as their sodium salts by reduction of **1** and **1′** with excess sodium amalgam in THF (Scheme 6). The solid-state structure of **6′** (*ν*(NN) = 1878 cm^–1^) illustrates that, in addition to acting as a hydrogen-bond acceptor, the tertiary amine in the secondary coordination sphere can serve as a Lewis base and interact with a Lewis acidic countercation such as [Na(THF)_3_]^+^ (Fig. 5). In this case, the N–Na distances are sufficiently long that the six-membered azacycle maintains a distorted chair conformation to accommodate this interaction. In the solid state the Fe–N–N angle, which is typically very close to 180° in terminal Fe–N_2_ complexes,^11,18,25^ is distorted to 171.7° due to interaction with the Na cation; the cation position is constrained by coordination to the pendant amine in the ligand. In contrast, the crystal structure of **6** (*ν*(NN) = 1874 cm^–1^) shows intermolecular coordination of the sodium cation to the amine of a neighboring molecule, forming an infinite chain structure; in this case the azacycle again adopts a chair conformation and the Fe–N–N angle is nearly linear (Fig. 5).

{[SiPiPr3]FeN_2_}^–^ can catalyze N_2_-to-NH_3_ conversion with poor efficiency at very high acid/reductant loading,^26^ and at lower loadings of 48 equiv. HBArF4·2Et_2_O and 50 equiv. KC_8_ generates 0.8 ± 0.4 equivalents of NH_3_ per Fe (Et_2_O, –78 °C, 1 atm N_2_).^11*a*^ In contrast, exposure of **L_1_** FeN_2_^–^ and **L_2_** FeN_2_^–^ complexes **6** and **6′** to analogous reaction conditions affords no detectable NH_3_. This dichotomy in reaction profile is interesting; the stoichiometric reactions of these respective species with acid provide some clues as to the reason for the disparate reactivity.

In the case of {[SiPiPr3]FeN_2_}^–^, treatment with one equivalent of either HBArF4·2Et_2_O or the tertiary ammonium acid [HN^i^Pr_2_Et][BArF4] at –78 °C results in clean oxidation to [SiPiPr3]FeN_2_, a reaction which is believed to proceed with formal loss of ½H_2_ from a transient Fe–N=NH intermediate.^15^ However, with either **6** or **6′**, the same reaction conditions instead result in immediate generation of the hydride complexes **L_1_** Fe(N_2_)(H) and **L_2_** Fe(N_2_)(H) (Scheme 6). Given that formation of a transient Fe–N=NH species is believed to be a necessary first step in the transformation of N_2_ to NH_3_ on complexes of these types,^15,26,27^ it seems plausible that **6** and **6′** do not give rise to Fe–N=NH species on exposure to acid.

**Fig. 6:**
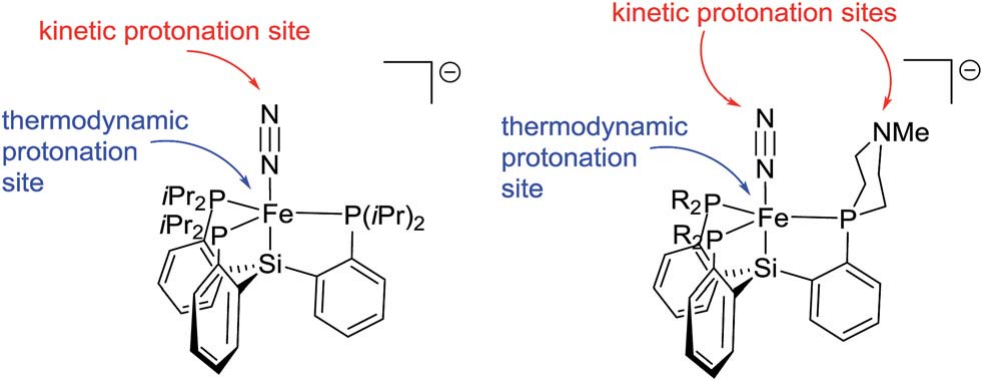
Kinetic and thermodynamic protonation sites of {[SiPR3]FeN_2_}^–^ anions.

The difference in product profiles between {[SiPiPr3]FeN_2_}^–^ and **L_1_** FeN_2_^–^ or **L_2_** FeN_2_^–^ upon treatment with acid can be rationalized by considering the kinetically and thermodynamically preferred sites of protonation in these complexes. The metal center is the thermodynamically preferred site of protonation in each system (*i.e.*, to afford an LFe(N_2_)(H) product); for **6**, DFT computations suggest the proton affinity of the iron center is at least 20 kcal mol^–1^ higher than for protonation at N_2_ or at the donor arm amine position (see ESI† for details). However, in the absence of an exposed basic site on the ligand, the kinetic site of protonation is most likely the terminal N-atom of the N_2_ ligand due to steric crowding at the iron center. If we presume protonation at the N_2_ transiently generates an Fe–N=NH species with {[SiPiPr3]FeN_2_}^–^ we can conclude that there is thereafter no kinetically facile pathway for the proton to then migrate to Fe; therefore the Fe–N=NH species instead decomposes *via* other (presumed bimolecular) pathways that release H_2_. When a tertiary amine is instead present in a phosphine donor arm, the amine can serve as the kinetic site of protonation, and can likely act as a proton shuttle to transfer a proton from N_2_ to the iron center, leading to stable iron hydride complexes (Fig. 6). A similar observation has been made in the case of protonation of anionic tungsten N_2_ complexes with and without pendant amines in the ligand.^12b,c^

We have previously observed the formation of iron hydride species during the course of catalytic N_2_-to-NH_3_ conversion. In the case of NH_3_ production catalyzed by the tris(phosphine)borane complex {[TP^iPr^B]FeN_2_}^–^, we have demonstrated that such a hydride species most likely serves as an off-path catalyst resting state.^26^ While the mechanisms of N_2_-to-NH_3_ conversion on the analogous alkyl- and silyl-supported complexes, {[CPiPr3]FeN_2_}^–^ and {[SiPiPr3]FeN_2_}^–^, have not yet been as thoroughly studied, we have proposed that the [EPiPr3]Fe(N_2_)(H) (E = C, Si) hydride complexes may be thermodynamic sinks that irreversibly deactivate the catalyst when formed under the reaction conditions.^11*b*^ The evidence presented here corroborates this, and supports the idea that efficient molecular catalysts for N_2_-to-NH_3_ conversion must either avoid the formation of metal hydrides, or facilitate kinetically competent pathways for these hydride complexes to reenter the catalytic cycle, likely through the evolution of H_2_.^26^

## Conclusions

We have demonstrated the incorporation of secondary-sphere interactions into iron–phosphine scaffolds relevant to synthetic nitrogen fixation. The presence of modestly strong hydrogen bonds between pendant ligand functionalities and possible N_2_ reduction products, ammonia and hydrazine, has been demonstrated; the ligand donor arms respond to the presence of favorable H-bonding interactions with a coordinated N_*x*_H_*y*_ substrate *via* conformational flipping of the cyclic phosphine/amine donor arm from a chair to a boat confirmation. While these new phosphine/amine ligands demonstrate an approach to tuning the secondary coordination sphere in Fe–N_2_ systems, the present systems do not show improved catalysis for N_2_ fixation but instead completely shut down such function. Kinetically facile formation of iron hydride products occurs instead due to the presence of the pendant amine proton shuttle. As the modified complexes are not competent catalysts, caution must be exercised in extrapolating these results to the catalytic systems, including the nitrogenase cofactor itself. Nevertheless, further tuning of the scaffold, such as additional steric protection of the metal center to hinder protonation, better p*K*_a_-tuning of the pendant base, or design of a system where hydrogen-bonding is less conformationally disfavored, may improve the reactivity. Work to further explore structure–function relationships in systems of these types is underway.
