# Supplementary material for: Exploring secondary-sphere interactions in Fe–N x H y complexes relevant to N2 fixation
Source: Chem Sci. 2016 Dec 8;8(3):2321–8. doi: 10.1039/c6sc04805f (PMC5363375; doi:10.1039/c6sc04805f)
Supplement: Supplementary file 1 [file SC-008-C6SC04805F-s001.pdf]

Electronic Supplementary Material (ESI) for Chemical Science.
This journal is © The Royal Society of Chemistry 2016

Supporting Information for:

Exploring secondary-sphere interactions in Fe-N_xH_y complexes relevant to N_2 fixation

*Sidney E. Creutz and Jonas C. Peters**

Division of Chemistry and Chemical Engineering, California Institute of Technology, Pasadena, CA 91125, United States

E-mail: jpeters@caltech.edu

I. General……………………………………………………………………………p. S2

II. Synthetic Procedures……………………………………………………..p. S3

III. Spectroscopic Characterization of Compounds……………p. S14

IV. Reactivity Studies………………………………………………………p. S30

V. Computational Details……………………………………………….p. S39

VI. Crystallographic Details……………………………………………p. S42

VII. References………………………………………………………………p. S55

I. General

General information. All syntheses and measurements, unless otherwise stated, were carried out under an inert atmosphere (N_2) in a glovebox or using standard Schlenk techniques, and solvents were dried and degassed by thoroughly sparging with N_2 and then passing through an activated alumina column in a solvent purification system supplied by SG Water, LLC. Combustion analyses were carried out by Midwest Microlabs (Indianapolis). Non-halogenated solvents were tested with a standard purple solution of sodium benzophenone ketyl in tetrahydrofuran in order to confirm effective moisture removal. Diethyl (2-bromophenyl)phosphonate,[1] Fc[BAr^F_4] (Ar^F = 3,5trifluoromethylphenyl),[2] $HBAr^F_4 \cdot 2Et_2O$ (Ar^F = 3,5-bistrifluoromethylphenyl),[3] KC_8,[4] Bis(2diisopropylphosphinophenyl)chlorosilane,[5] {[SiP^{iPr}_3]FeN_2}{Na(12-crown-4)$_2$},[6] and {[SiP^{iPr}_3]FeN_2H_4}{BAr^F_4}[6] were prepared according to literature procedures. All other reagents were purchased from commercial vendors and used without further purification unless otherwise stated.

Physical methods. Optical spectroscopy measurements were taken on a Cary 50 UV−vis spectrophotometer using a 1-cm two-window quartz cell. Fourier transform infrared ATR spectra were collected on a Thermo Scientific Nicolet iS5 Spectrometer with diamond ATR crystal. Solution phase magnetic measurements were performed by the method of Evans.[7] ^{1}H and ^{13}C chemical shifts are reported in ppm relative to tetramethylsilane, using residual solvent resonances as internal standards. ^{31}P chemical shifts are reported in ppm relative to 85% aqueous H_3PO_4. X-band EPR spectra were obtained on a Bruker EMX spectrometer; solutions prepared as frozen glasses in 2-MeTHF.

II. Synthetic Procedures

(2-bromophenyl)divinylphosphine oxide. Diethyl (2-bromophenyl)phosphonate (15.1 g, 0.0515 mol) was transferred to a 1L roundbottom Schlenk flask under N_2 with a large stir bar. TMSBr (17.2 mL, 0.130 mol, neat) was added by syringe at room temperature and the resulting solution was stirred at room temperature for three hours. The reaction was then concentrated *in vacuo* with mild heating (50 ^{o}C) until a pale yellow oil remained. This oil was redissolved in 80 mL of dry dichloromethane and then 20 drops of dry DMF were added. Oxalyl chloride (17.6 mL, 0.205 mol, neat) was added via syringe; vigorous bubbling was observed and the color of the reaction mixture darkened slightly. This mixture was stirred for one hour at room temperature and then concentrated *in vacuo* with mild heating (50 ^{o}C). The resulting residue was redissolved in 300 mL of dry, degassed THF and cooled to -78 ^{o}C. Vinyl magnesium bromide (1.0 M in THF, 102.9 mL, 0.103 mol) was diluted with an additional 150 mL of dry, degassed THF in an addition funnel and added slowly over the course of four hours to the cold reaction mixture. After the addition was completed the reaction mixture, still at -78 ^{o}C, was stirred for an additional 2 hours. Then the reaction was quenched by swiftly pouring it into a stirring mixture of ~150 mL of HCl and ~300 mL of ice. After reaching room temperature the quenched solution was extracted with dichloromethane (2 x 300 mL). The organic washings were combined and washed with 2 M NaOH and then saturated brine solution. The organic fraction was dried over magnesium sulfate, filtered, and concentrated to give a pale, sometimes cloudy oil (10.6 g, 0.0440 mol, 85%). This material was typically of sufficient purity to be used in the next step without further purification; however if desired analytically pure material can be obtained by column chromatography on silica gel (2%

MeOH in ethyl acetate). ^{1}H NMR ($CDCl_3$, 300 MHz, 298 K): δ 8.14 (ddd, J = 12, 8, 2 Hz, 1H, CH_{Ar}), 7.63 (ddd, J = 8, 4, 1 Hz, 1H, CH_{Ar}), 7.44 (m, 2H, CH_{Ar}), 6.83 (ddd, J = 27.4, 18.7, 12.6 Hz, 2H, CH_{vinyl}), 6.31 (m, 4H, CH_{vinyl}). ^{31}P NMR ($CDCl_3$, 121 MHz, 298 K): δ 17.8 ppm.

4-(2-bromophenyl)-1-methyl-1,4-azaphosphinane-4-oxide. (2-bromophenyl)-divinylphosphine oxide (10.6 g, 0.0440 mol) was transferred (using a small amount of THF) to a 500 mL round bottom flask with a stir bar and 200 mL of water were added. Then 4.0 mL of a 40 wt% aqueous solution of methylamine (1.2 equiv) were added. This mixture was refluxed for 4 hours, then cooled to room temperature and extracted with dichloromethane. The dichloromethane portions were dried over sodium sulfate, filtered, and concentrated to give an off-white solid. This residue was purified by column chromatography on silica gel using 2% NEt_3 in 5:1 DCM:MeOH. The desired product was isolated as a white solid (8.5 g, 0.0295 mmol, 67%). ^{1}H NMR ($CDCl_3$, 400 MHz, 298 K): δ 7.91 (m, 1H, CH_{Ar}), 7.49 (m, 1H, CH_{Ar}), 7.33 (t, 1H, CH_{Ar}), 7.24 (t, 1H, CH_{Ar}), 2.6-3.0 (m, 6H, azaphosphinane-CH_2), 2.27 (s, 3H, N-CH_3), 1.87 (m, 2H, azaphosphinane-CH_2) ppm. ^{31}P NMR ($CDCl_3$, 161.9 MHz, 298 K): δ 28.6 ppm. ^{13}C NMR ($CDCl_3$, 100.6 MHz, 298 K): δ 135.3 (d, J_{CP} = 7.3 Hz, C_{Ar}), 134.3 (d, J_{CP} = 7.8 Hz, C_{Ar}), 133.5 (d, J_{CP} = 2.5 Hz, C_{Ar}), 131.9 (d, J_{CP} = 94.8 Hz, C_{Ar}), 127.4 (d, J_{CP} = 9.9 Hz, C_{Ar}), 124.2 (d, J_{CP} = 6.0 Hz, C_{Ar}), 51.2 (d, J_{CP} = 7.4 Hz, P-$(CH_2CH_2)_2NCH_3$), 46.3 (s, P-$(CH_2CH_2)_2NCH_3$), 26.7 (d, J_{CP} = 66.5 Hz, P-$(CH_2CH_2)_2NCH_3$) ppm.

4-(2-bromophenyl)-1-methyl-1,4-azaphosphinane (L_0). 4-(2-bromophenyl)-1-methyl-1,4azaphosphinane-4-oxide (2.16 g, 7.50 mmol) was dissolved in 80 mL of dry, degassed DCM to give a clear, homogeneous solution. Then neat oxalyl chloride (629 μL, 1.0 equiv) was added by syringe in one portion. Over the next few minutes an off-white precipitate developed and the solution color changed to pale yellow. This reaction mixture was stirred for one hour and then

concentrated to dryness *in vacuo*. Then, 80 mL of dry, degassed THF was added; the resulting suspension was stirred vigorously and cooled to -78° C. Lithium aluminum hydride (1.0 M solution in THF, 7.56 mL, 1.0 equiv) was added *via* syringe over 5 minutes. Bubbling was observed. After 30 minutes the reaction was transferred to a 0 °C bath and stirred for an additional one hour, at which point the solution is pale yellow and homogeneous. The reaction is allowed to warm to room temperature for one hour, and then quenched with 3 mL of degassed ethyl acetate; a precipitate slowly develops. After about 20 minutes the volume is reduced *in vacuo* to 20 mL. Under continued anaerobic conditions, this mixture is diluted with 200 mL of DCM and washed with 100 mL of 1.0 M aqueous NaOH. The organic fraction is dried over sodium sulfate, filtered, and concentrated to a pale orange, cloudy oil. This is extracted into pentane (2 x 30 mL) and filtered through Celite, removing some orange and white solids. The filtrate is concentrated to a white powder (1.72 g, 6.32 mmol, 84%). The material thus obtained is typically spectroscopically pure and is used in subsequent steps without additional purification, but can be further recrystallized from cold pentane if needed. ^{1}H NMR (C_6D_6, 400 MHz, 298 K): δ 7.34 (dd, *J* = 8.0, 3.0 Hz, 1H, CH_{Ar}), 7.06 (d, *J* = 8 Hz, 1H, CH_{Ar}), 6.92 (t, *J* = 8 Hz, 1H, CH_{Ar}), 6.70 (t, *J* = 7.6 Hz, 1H, CH_{Ar}), 2.63 (m, 2H, azaphosphinane-CH_2), 2.33 (m, 2H, azaphosphinane-CH_2), 2.035 (s, 3H, N-CH_3), 2.00 (m, 4H, azaphosphinane-CH_2) ppm. ^{31}P NMR (C_6D_6, , 161.9 MHz, 298 K): δ -41.2 ppm. ^{13}C NMR (C_6D_6, 100.6 MHz, 298 K): δ 140.7 (d, J_{CP} = 22.7 Hz, C_{Ar}), 132.9 (s, C_{Ar}), 131.4 (s, C_{Ar}), 129.0 (s, C_{Ar}), 128.9 (d, J_{CP} = 26.2 Hz, C_{Ar}), 126.948 (s, C_{Ar}), 53.5 (s, P-$(CH_2CH_2)_2NCH_3$), 46.6 (s, P-$(CH_2CH_2)_2NCH_3$), 23.7 (d, J_{CP} = 12.5 Hz, P-$(CH_2CH_2)_2NCH_3$) ppm.

[$SiP_2^{iPr}P^{NMe}$]H (L_1). 4-(2-bromophenyl)-1-methyl-1,4-azaphosphinane (1.511 g, 5.56 mmol) was dissolved in THF (20 mL) and cooled to -78 °C. *t*BuLi (1.7 M in pentane, 6.527 mL, 11.12 mmol) was added over 5 minutes and the resulting mixture was stirred at low temperature for 1.5 hours.

Bis(2-diisopropylphosphinophenyl)chlorosilane (2.505 g, 5.56 mmol) was added over five minutes as a solution in THF (10 mL). This reaction mixture was allowed to stir overnight while slowly warming to room temperature. The resulting yellow-orange solution was concentrated to dryness, extracted with benzene, and filtered through Celite. The filtrate was concentrated to give a white powder which was washed with pentane, giving 2.61 g of the desired product (4.29 mmol, 77%). ^{1}H NMR (C_6D_6, 300 MHz, 298 K): δ 7.60 (m, 1H, CH_{Ar}), 7.38 (m, 6H, CH_{Ar}), 7.18 (m, 2H, CH_{Ar}), 7.02 (t, J = 7 Hz, 3H, CH_{Ar}), 2.74 (m, 2H, azaphosphine-CH_2), 1.9-2.3 (m, 11H, -NCH_3, azaphosphine-CH_2, and –CH(CH_3)$_2$), 1.82 (m, 2H, azaphosphine-CH_2), 1.16 (m, 12H, CH(CH_3)$_2$), 0.97 (m, 12H, -CH(CH_3)$_2$) ppm. ^{13}C NMR (C_6D_6, 100.6 MHz, 298 K): δ 146.3 (d, J_{CP} = 52 Hz), 144.54 (d, J_{CP} = 17 Hz), 143.9 (d, J_{CP} = 42 Hz), 138.7 (d, J_{CP} = 13 Hz), 138.4 (d, J_{CP} = 14 Hz), 132.2 (s), 130.5 (s), 129.6 (s), 128.9 (s), 54.9 (s), 47.0 (s), 25.5 (m), 20.7 (d, J_{CP} = 17 Hz), 20.3 (m) ppm. NMR (C_6D_6, 121 MHz, 298 K): δ 0.8 (s, 2P), -48.4 (s, 1P) ppm.

[SiP$_3^{NMe}$]H (L$_2$). 4-(2-bromophenyl)-1-methyl-1,4-azaphosphinane (3.13 g, 11.5 mmol) was dissolved in THF (20 mL) and cooled to -78 °C. *t*BuLi (1.7 M in pentane, 13.53 mL, 23.0 mmol) was added over 5 minutes and the resulting mixture was stirred at low temperature for 1.5 hours. Trichlorosilane (380 μL, 3.76 mmol) was added in one neat portion. This reaction mixture was allowed to stir overnight while slowly warming to room temperature. The resulting yellow-orange solution was concentrated to dryness, extracted with benzene, and filtered through Celite. The filtrate was concentrated to give a white powder which was washed with pentane, giving 2.19 g of the desired product (3.61 mmol, 94%). ^{1}H NMR (C_6D_6, 300 MHz, 298 K): δ 7.65 (dd, J = 7.4, 3.3 Hz, 3H, CH_{Ar}), 7.44 (t, J = 6.2 Hz, 3H, CH_{Ar}), 7.22 – 7.12 (m, 3H, CH_{Ar}), 7.02 (t, J = 7.4 Hz, 3H, CH_{Ar}), 2.85 – 2.61 (m, 6H, azaphosphine-CH_2), 2.24 – 2.06 (m, 12H, azaphosphine-CH_2), 2.01 (s, 9H, -NCH_3), 1.73 (t, J = 12.1 Hz, 6H, azaphosphine-CH_2) ppm. ^{31}P NMR (C_6D_6, 121 MHz, 298

K): δ -49.4 (s) ppm. ^{13}C NMR (C_6D_6, 100.6 MHz, 298 K): δ 146.3 (d, J_{CP} = 16.8 Hz, C_{Ar}), 137.8 (d, J_{CP} = 13.6 Hz, C_{Ar}), 130.5 (s, C_{Ar}), 129.4 (s, C_{Ar}), 128.2 (C_{Ar}), 54.4 (s, P-($CH_2\mathit{C}H_2$)$_2$NCH$_3$), 46.7 (s, P-(CH_2CH_2)$_2$N$\mathit{C}H_3$), 25.4 (d, J_{CP} = 11.6 Hz, P-($\mathit{C}H_2CH_2$)$_2$NCH$_3$) ppm.

[SiP$^{iPr}_2$P^{NMe}]FeCl (1). [SiP$^{iPr}_2$P^{NMe}]H (2.61 g, 4.29 mmol) was combined with $FeCl_2$ (1.10 g, 8.68 mmol) in 50 mL of THF and vigorously stirred overnight to give a homogeneous yellow solution. This reaction mixture was cooled to -78 °C, and MeMgBr (2.86 mL, 3.0 M in THF, 8.58 mmol) was diluted with 18 mL of THF and added dropwise over 20 minutes to the reaction mixture. The reaction was allowed to warm slowly overnight with stirring. To the resulting dark brown reaction mixture was added dioxane (20 mL) and pentane (20 mL) and the mixture was stirred for an hour at room temperature and then filtered through Celite. The bright orange-yellow filtrate was concentrated to dryness, extracted with benzene, and filtered again. This was concentrated to give a yellow-orange solid which was washed with pentane to give **1** (2.57 g, 3.68 mmol, 85%). ^1H NMR (C_6D_6, 300 MHz, 298 K): δ 127.9, 80.1, 55.0, 26.7, 13.1, 10.9, 6.6, 6.3, 5.7, 5.3, 5.0, 2.9, 3.0, -1.4, -2.6 ppm. μ_{eff} (Evans Method, C_6D_6, 298 K): 3.0 μ_B. Anal. Calcd. for $C_{35}H_{51}ClFeNP_3Si$: C, 60.22; H, 7.36; N, 2.01. Found: C, 59.92; H, 7.27; N, 2.11.

[SiP$^{NMe}_3$]FeCl (1'). [SiP$_3^{NMe}$]H (2.19 g, 3.62 mmol) was combined with $FeCl_2$ (917 mg, 7.23 mmol) in 50 mL of THF and vigorously stirred overnight to give a homogeneous yellow solution. This reaction mixture was cooled to -78 °C, and MeMgBr (2.41 mL, 3.0 M in THF, 7.23 mmol) was diluted with 18 mL of THF and added dropwise over 20 minutes to the reaction mixture. The reaction was allowed to warm slowly overnight with stirring. To the resulting dark brown reaction mixture was added dioxane (20 mL) and pentane (20 mL) and the mixture was stirred for an hour at room temperature and then filtered through Celite. The bright orange-yellow filtrate was concentrated to dryness, extracted with benzene, and filtered again. This was concentrated to give

a yellow-orange solid which was washed with pentane to give **1'** (0.910 g, 1.31 mmol, 36%). Crystals suitable for X-ray diffraction were grown by vapor diffusion of pentane into a concentrated benzene solution. ^{1}H NMR (C_6D_6, 300 MHz, 298 K): δ 158, 33.9, 17.1, 10.6, 5.9, 5.3, 2.6, -1.1 ppm. μ_{eff} (Evans Method, C_6D_6, 298 K): 3.0 μ_B. Anal. Calcd. for $C_{33}H_{45}ClFeN_3P_3Si$: C, 56.95; H, 6.52; N, 6.04. Found: C, 56.30; H, 6.52; N, 5.72.

[SiP$^{iPr}_2$P^{NMe}]FeN$_2$ (2). [SiP$^{iPr}_2$P^{NMe}]FeCl (245 mg, 0.351 mmol) was dissolved in THF (10 mL) and stirred over 1% sodium-mercury amalgam (9.7 mg Na, 0.422 mmol, 1.2 equiv.) for three hours. The reaction mixture was concentrated to dryness, extracted into benzene, filtered through Celite, and concentrated to dryness. The residue was taken up in pentane and again filtered through Celite, then concentrated to give an orange powder which was recrystallized from cold pentane to give an orange microcrystalline solid which was washed with cold pentane (141 mg, 0.204 mmol, 58%). ^{1}H NMR (C_6D_6, 300 MHz, 298 K): δ 69.1, 26.9, 19.3, 8.3, 8.0, 6.4, 5.9, 4.7, 3.9, 3.2, 2.4, 1.8, 2.1, -13.9 ppm. μ_{eff} (Evans Method, C_6D_6, 298 K): 2.1 μ_B. IR (thin film deposited from C_6D_6): 2005 cm^{-1} (ν(N-N)). We were unable to obtain satisfactory EA after several attempts, likely due to some lability of the coordinated N_2 ligand.

[SiP$^{NMe}_3$]FeN$_2$ (2'). [SiP$^{NMe}_3$]FeCl (500 mg, 0.716 mmol) was dissolved in THF (10 mL) and stirred over 1% sodium-mercury amalgam (18.2 mg Na, 0.791 mmol, 1.1 equiv.) for three hours. The reaction mixture was concentrated to dryness, extracted into benzene, filtered through Celite, and concentrated to dryness. The residue was taken up in 1:1 benzene:pentane and again filtered through Celite, then concentrated to give a yellow-orange powder which was washed with pentane to give spectroscopically clean product (245 mg, 50%). Single crystals suitable for X-ray diffraction were grown by cooling a concentrated Et_2O solution in a -35 ^oC freezer overnight. ^{1}H NMR (C_6D_6, 300 MHz, 298 K): δ 85.1, 19.9, 10.6, 8.31, 7.20, 6.95, 6.38, 5.58, 3.03 ppm. μ_{eff}

(Evans Method, C_6D_6, 298 K): 2.1 μ_B. IR (thin film deposited from C_6D_6): 2007 cm^{-1} (ν(N-N)). We were unable to obtain satisfactory EA after several attempts, likely due to some lability of the coordinated N_2 ligand.

{[SiP$^{iPr}{}_2$P^{NMe}]FeNH$_3$}{BAr$^F{}_4$} (3). [SiP$^{iPr}{}_2$P^{NMe}]FeN$_2$ (100 mg, 0.145 mmol) was dissolved in THF (5 mL) and a solution of [Fc][BAr$^F{}_4$] (144 mg, 0.95 equiv) in THF (5 mL) was added. The orange solution darkened to brown and the reaction mixture was allowed to stir at room temperature for one hour before being transferred to a Schlenk tube and degassed by briefly exposing to dynamic vacuum. This solution was exposed at room temperature to 1 atm of NH_3 (which had been dried by stirring over sodium at -78 ^oC). The color changed from brown to bright orange. The reaction mixture was concentrated to dryness and the orange residue was extracted with ether and filtered through Celite. The ether solution was concentrated to 3 mL, layered with pentane, and allowed to stand overnight, resulting in the formation of orange crystals which were thoroughly washed with pentane (162 mg, 70%). Crystals suitable for X-ray analysis were grown by vapor diffusion of pentane into an ether solution. ^{1}H NMR (5:1 C_6D_6:d_8-THF, 300 MHz, 298 K): δ 91.2, 59.9, 46.0, 29.0, 10.34, 8.32 (BAr$^F{}_4$), 7.70 (BAr$^F{}_4$), 5.89, 5.59, 5.28, 3.79, -0.35, -2.72, -3.28 ppm. ^{19}F NMR (5:1 C_6D_6:d_8-THF, 282 MHz, 298 K): δ -62 ppm. μ_{eff} (Evans Method, 4:1 C_6D_6:d_8THF, 298 K): 3.3 μ_B. Anal. Calcd. for $C_{67}H_{66}BF_{24}FeN_2P_3Si{\cdot}Et_2O$: C, 52.74; H, 4.74; N, 1.73. Found: C, 52.64; H, 4.54; N, 1.42.

{[SiP$^{NMe}{}_3$]FeNH$_3$}{BAr$^F{}_4$} (3'). (a) [SiP$^{NMe}{}_3$]FeN$_2$ (42 mg, 0.061 mmol) was dissolved in THF (5 mL) and a solution of [Fc][BAr$^F{}_4$] (61 mg, 0.95 equiv) in THF (5 mL) was added. The orange solution darkened to brown and the reaction mixture was allowed to stir at room temperature for one hour before being transferred to a Schlenk tube and degassed by briefly exposing to dynamic vacuum. This solution was exposed at room temperature to 1 atm of NH_3 (which had been dried

by stirring over sodium at -78 °C). The color changed from brown to bright orange. The reaction mixture was concentrated to dryness and the orange residue was extracted with ether and filtered through Celite. The ether solution was concentrated to 3 mL, layered with pentane, and allowed to stand overnight, resulting in the formation of orange crystals which were thoroughly washed with pentane (69 mg, 73%). (b) $[SiP^{NMe}{}_3]FeN_2$ (45 mg, 0.065 mmol) was dissolved in THF (5 mL) and a solution of $[Fc][BAr^F{}_4]$ (62 mg, 0.9 equiv) in THF (5 mL) was added. The orange solution darkened to brown and the reaction mixture was allowed to stir at room temperature for two hours before being cooled to -78 °C; excess hydrazine (100 μL) was then added. The reaction was allowed to warm to room temperature for 10 minutes during which time it turned bright orange; it was then concentrated *in vacuo*, extracted with Et_2O, filtered through Celite, and concentrated down to 2 mL of a bright orange Et_2O solution. This solution was layered with pentane and stored at -35 °C overnight, resulting in the formation of feathery orange crystals; the supernatant was decanted and the crystals were washed with pentane and dried to give the desired product as an orange crystalline solid (37 mg, 41%). Crystals suitable for X-ray analysis were grown by vapor diffusion of pentane into an ether solution. ^1H NMR (5:1 C_6D_6:d_8-THF, 300 MHz, 298 K): δ 122 (v.b.), 41 (v.b.), 13.1, 8.37 ($BAr^F{}_4$), 7.74 ($BAr^F{}_4$), 5.69, 4.54, 3.46, -0.68, -1.78 ppm. ^{19}F NMR (5:1 C_6D_6:d_8-THF, 282 MHz, 298 K): δ -62 ppm. μ_{eff} (Evans Method, 4:1 C_6D_6:d_8THF, 298 K): 3.2 μ_B. Anal. Calcd. for $C_{65}H_{66}BF_{24}FeN_4P_3Si$: C, 50.67; H, 3.93; N, 3.64. Found: 50.11; H, 3.86; N, 3.40.

$\{[SiP^{iPr}{}_2P^{NMe}]FeN_2H_4\}\{BAr^F{}_4\}$ (4). $[Fc][BAr^F{}_4]$ was added to $[SiP^{iPr}{}_2P^{NMe}]FeN_2$ (130 mg, 0.188 mmol) in THF (10 mL) and the resulting brown-orange solution was stirred at room temperature for 2 hours before cooling to -78 °C. Excess neat hydrazine (100 μL) was added and then the

solution was allowed to warm to room temperature while stirring for 30 minutes, then concentrated *in vacuo*. The orange residue was taken up in Et_2O (5 mL) and filtered through Celite, then layered with pentane (10 mL) and allowed to stand overnight. The resulting dark red crystals were washed copiously with pentane and then dried *in vacuo* to give the desired product (200 mg, 68%). Single crystals suitable for X-ray diffraction were grown by vapor diffusion of pentane into an ether solution. ^{1}H NMR (4:1 C_6D_6:d_8THF, 300 MHz, 298 K): δ 46.8, 36.8, 34.6, 4.7, 11.6, 8.23, 7.62, 6.24, 5.84, 5.39, 2.97, 1.91, -2.4 (br), -2.94, -3.39, -3.92 ppm. ^{19}F NMR (4:1 C_6D_6:d_8THF, 282 MHz, 298 K): δ -62 ppm. μ_{eff} (Evans Method, 4:1 C_6D_6:d_8THF, 298 K): 3.3 μ_B. Anal. Calcd. for $C_{67}H_{67}BF_{24}FeN_3P_3Si \cdot 1.5Et_2O$: C, 52.53; H, 4.95; N, 2.52. Found: C, 52.27; H, 4.45; N, 2.00.

{[SiP$^{iPr}_2$P^{NMe}]FeN$_2$}{Na(THF)$_2$} (6). [SiP$^{iPr}_2$P^{NMe}]FeCl (214 mg, 0.307 mmol) was dissolved in THF (10 mL) and stirred over an excess of sodium amalgam (37 mg Na, 1 wt% in Hg) overnight. The dark red solution was then decanted from the excess mercury, dried *in vacuo*, extracted with Et_2O, filtered through Celite, and again dried *in vacuo* to give a dark red residue. This residue was taken up in 2:1 Et_2O:THF, layered with pentane, and allowed to stand overnight to give dark crystals of the desired product (115 mg, 44%). Single crystals suitable for X-ray diffraction were grown by vapor diffusion of pentane into a concentrated 2:1 Et_2O:THF solution. ^{1}H NMR (d_8THF, 300 MHz, 298 K): δ 7.95 (m, 3H, CH_{Ar}), 7.51 (m, 1H, CH_{Ar}), 7.20 (m, 2H, CH_{Ar}), 6.93-6.80 (m, 6H, CH_{Ar}), 3.25 (m, 2H, azaphosphine-CH_2), 2.78 (m, 4H, -CH(CH_3)$_2$), 2.32 (m, 7H, -NCH_3 and azaphosphine-CH_2), 1.07 (m, 18H, -CH(CH_3)$_2$), 0.63 (m, 6H, -CH(CH_3)$_2$) ppm. ^{31}P NMR (d_8THF, 121 MHz, 298 K): δ 92 (br s, 2P, -P^{iPr2}), 37 (br s, 1P, -P(CH_2CH_2)$_2$NMe) ppm. IR: solid powder on ATR, 1874 cm^{-1} (ν(N-N)); thin film deposited from THF, 1880 cm^{-1} (ν(N-N)): . Anal. Calcd. for $C_{43}H_{65}FeN_3NaO_2P_3Si$: C, 60.35; H, 7.66; N, 4.91. Found: C, 60.30; H, 7.69; N, 5.00.

{[SiP$^{NMe}_3$]FeN$_2$}{Na(THF)$_3$} (6'). [SiP$^{NMe}_3$]FeCl (203 mg, 0.292 mmol) was dissolved in THF (10

ml) and stirred vigorously over an excess of sodium amalgam (30 mg Na in 4.0 g Hg) overnight. The dark red reaction mixture was then filtered through Celite and concentrated to a volume of 2 mL. This was layered with 5 mL of Et_2O and 10 mL of pentane and allowed to stand overnight to allow the formation of dark crystalline solids. The supernatant was decanted and the crystals were washed with pentane, benzene, and additional pentane and then dried *in vacuo* to give the desired product (175 mg, 65%). Crystals suitable for X-ray diffraction were grown by vapor diffusion of pentane into a 2:1 ether:THF solution. ^{1}H NMR (d_8-THF, 400 MHz, 298 K): δ 8.083 (d, 3H, J = 7 Hz, CH_{Ar}), 7.52 (d, 3H, J = 7 Hz, CH_{Ar}), 7.03 (t, 3H, J = 7 Hz, CH_{Ar}), 6.94 (t, 3H, J = 7 Hz, CH_{Ar}), 3.46 (m, 6H, azaphosphine-CH_2), 2.91 (m, 6H, -CH(CH_3)$_2$), 2.38 (s, 9H, -NCH_3) ppm. ^{31}P NMR (d_8-THF, 161 MHz, 298 K): δ 44 (br s) ppm. IR: solid powder on ATR, 1878 cm^{-1} (ν(N-N)); thin film deposited from THF, 1870 cm^{-1} (ν(N-N)). Anal. Calcd. for $C_{45}H_{66}FeN_5NaO_3P_3Si$: C, 58.44; H, 7.19; N, 7.57. Found: C, 57.79; H, 6.96; N, 7.42.

[SiP$^{iPr}_2$P^{NMe}]FeNH$_2$ (5). [SiP$^{iPr}_2$P^{NMe}]FeCl (148 mg, 0.217 mmol) was dissolved in THF (5 mL) in a Schlenk bomb and excess sodium amide (65 mg) was added. The reaction mixture was frozen at 77 K and the headspace was evacuated. Liquid ammonia (~2 mL), which had been previously dried by stirring over excess sodium metal, was condensed onto the reaction mixture which was then thawed to give a homogeneous yellow-orange mixture which was stirred at -20 °C for one hour until the color darkened to red-orange. The solvent was then removed *in vacuo* and the residue was extracted with pentane and filtered through Celite. The red-orange residue was recrystallized by slow evaporation of pentane, and the resulting red crystals were washed with cold pentane to give the desired product (110 mg, 0.162 mmol, 75%). Crystals suitable for X-ray diffraction were grown by slow evaporation of a pentane solution into HMDSO. ^{1}H NMR (C_6D_6, 300 MHz, 298 K): δ 134.6, 87.8, 70.2, 27.8, 8.72, 7.05, 6.46, 6.19, 4.88, 4.24, 2.04, 1.88, 0.54,

0.46, -2.5 ppm. μ_{eff} (Evans Method, C_6D_6, 298 K): 3.1μ_B. Anal. Calcd. for $C_{35}H_{53}FeN_2P_3Si$: C, 61.94; H, 7.87; N, 4.13. Found: C, 62.16; H, 7.93; N, 3.36. The identity of the $–NH_2$ ligand was further confirmed by treating an Et_2O solution of the product with excess HCl in Et_2O and then subjecting the resulting solution to a standard workup and indophenol test for ammonia (see below). Quantitative ammonia (1.0 equiv.) was detected.

III. Spectroscopic characterization of compounds

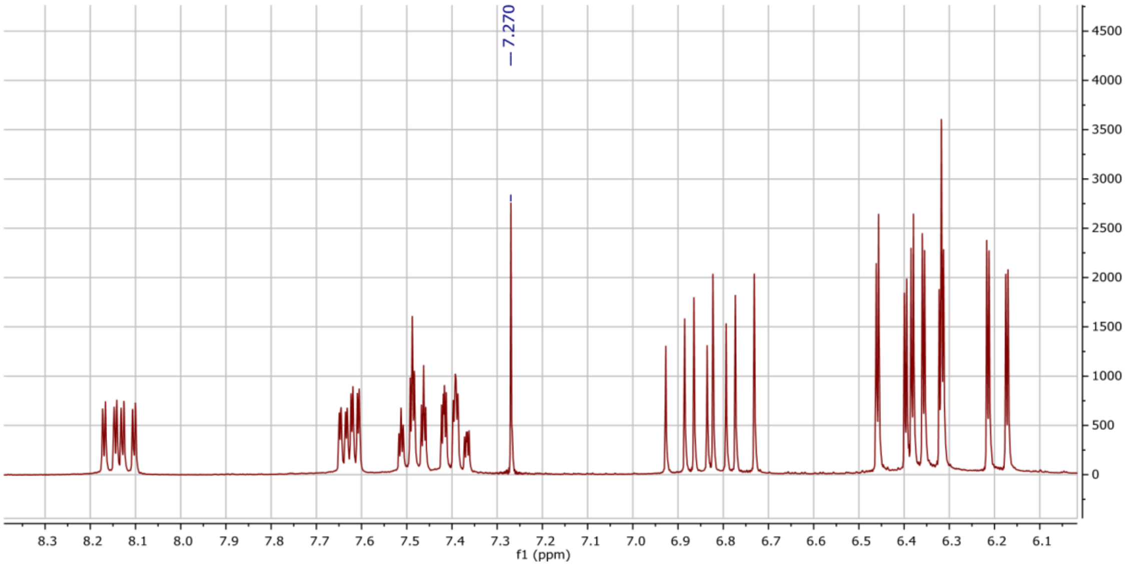

Figure S1. ^{1}H NMR of (2-bromophenyl)divinylphosphine oxide ($CDCl_3$, 300 MHz, 298 K). For clarity, only the downfield region is shown; no resonances appear outside of the region depicted.

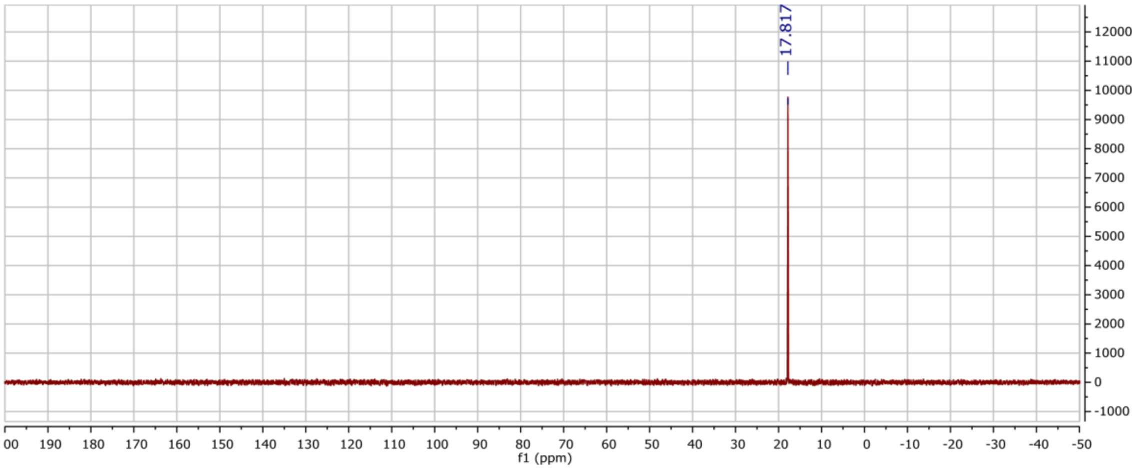

Figure S2. ^{31}P NMR of (2-bromophenyl)-divinylphosphine oxide ($CDCl_3$, 121 MHz, 298 K).

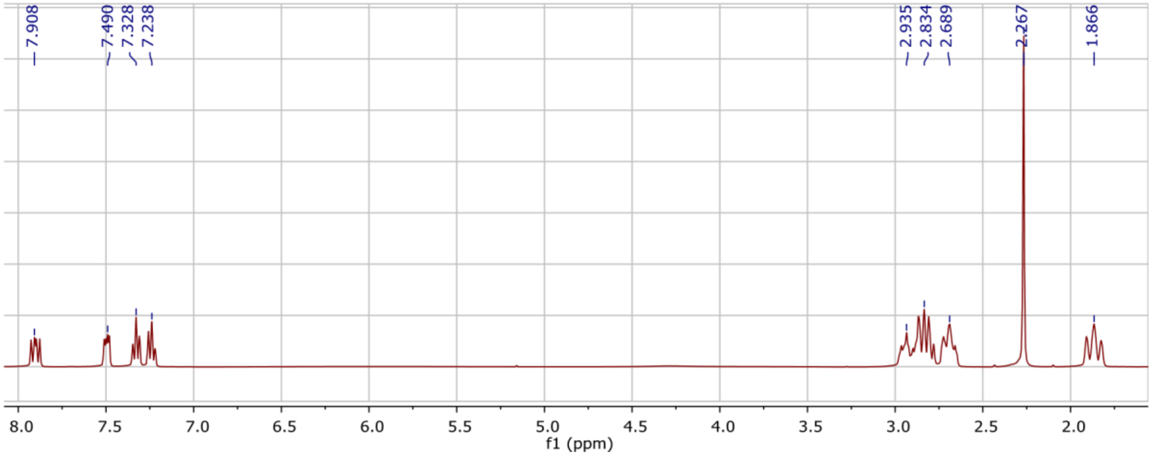

Figure S3. ^{1}H NMR of 4-(2-bromophenyl)-1-methyl-1,4-azaphosphinane-4-oxide ($CDCl_3$, 400 MHz, 298 K).

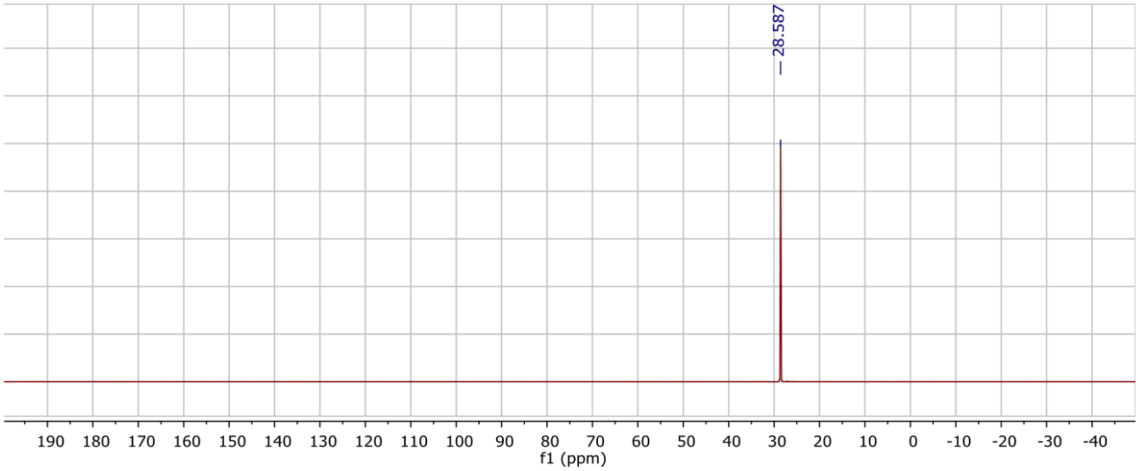

Figure S4. ^{31}P NMR of 4-(2-bromophenyl)-1-methyl-1,4-azaphosphinane-4-oxide ($CDCl_3$, 162 MHz, 298 K).

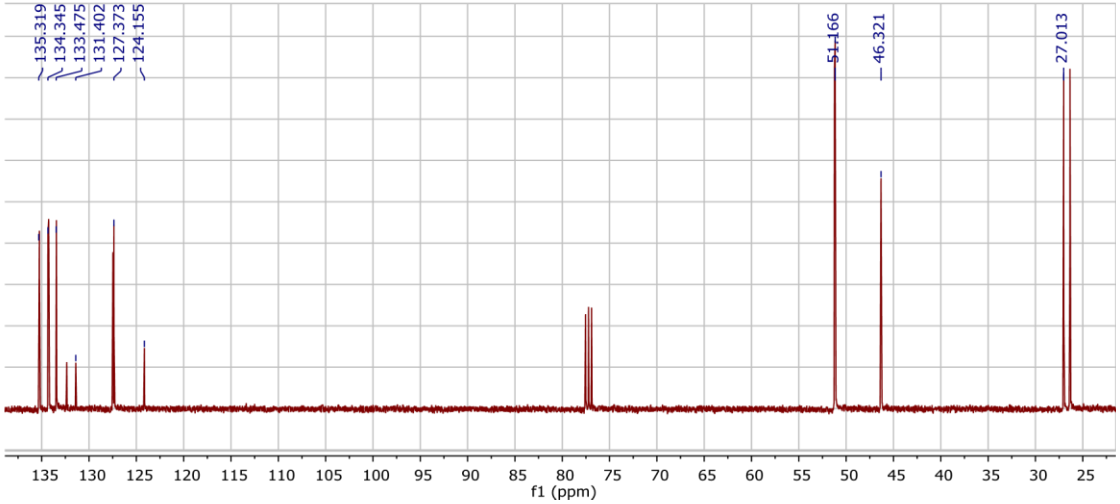

Figure S5. ^{13}C NMR of 4-(2-bromophenyl)-1-methyl-1,4-azaphosphinane-4-oxide ($CDCl_3$, 101 MHz, 298 K).

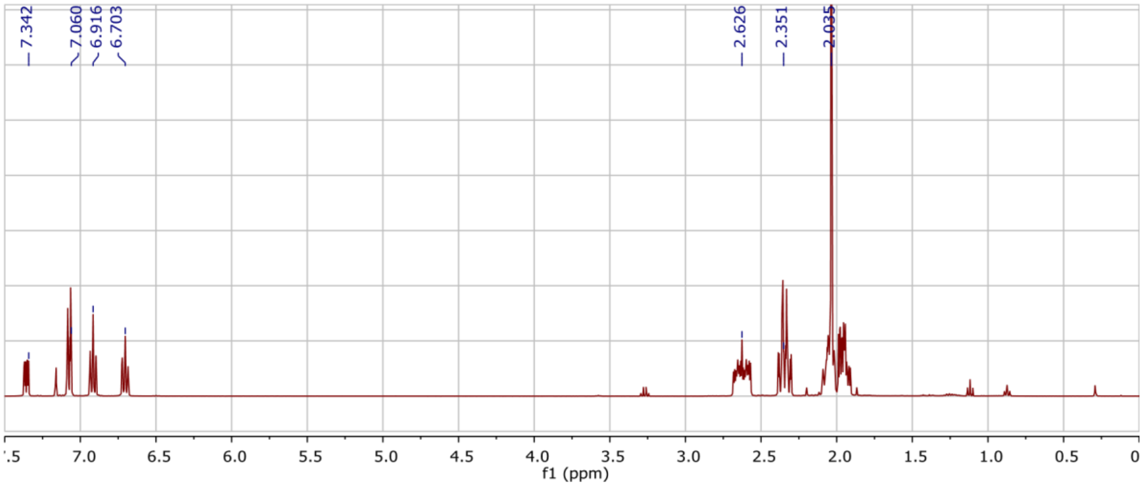

Figure S6. ^{1}H NMR of $\mathbf{L_0}$ (C_6D_6, 400 MHz, 298 K).

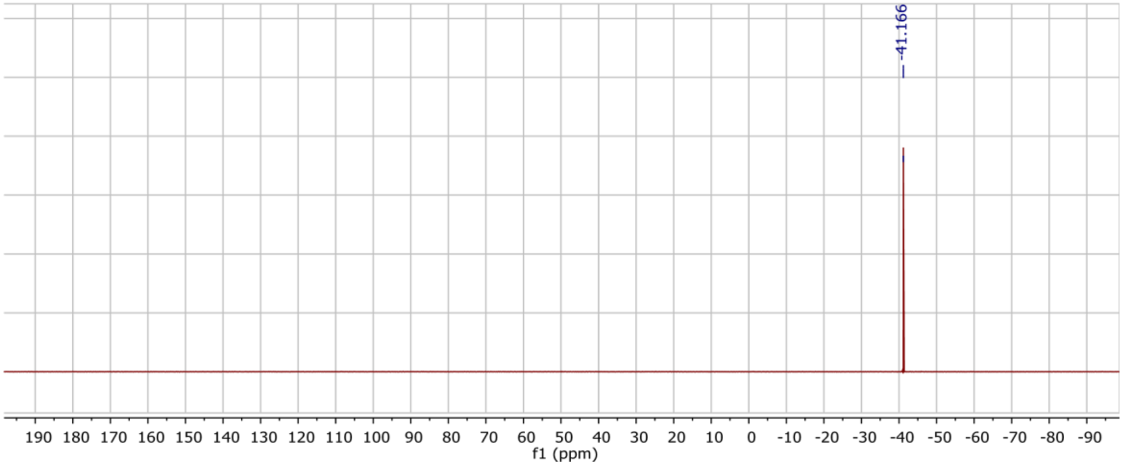

Figure S7. ^{31}P NMR of $\mathbf{L_0}$ (C_6D_6, 162 MHz, 298 K).

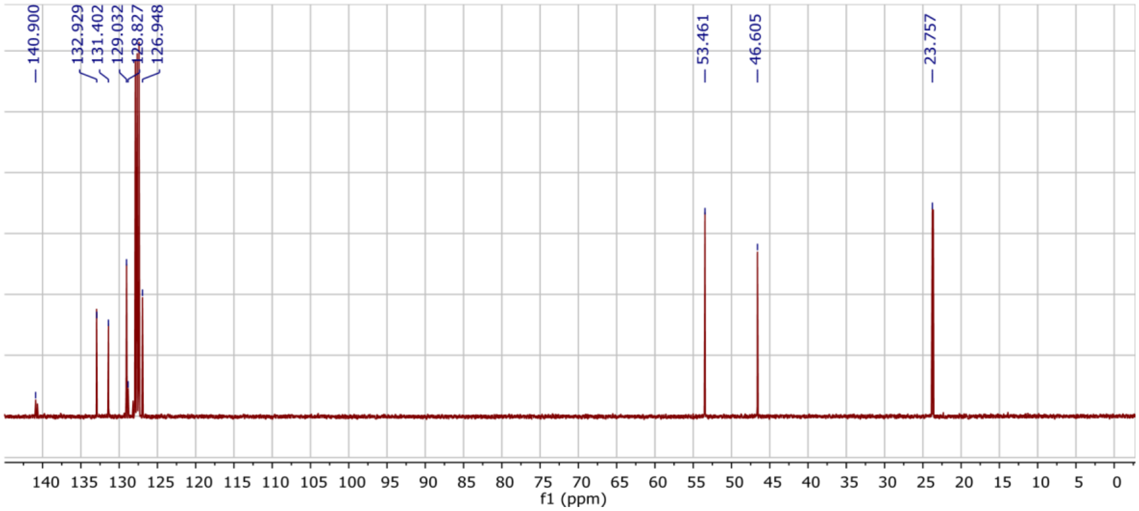

Figure S8. ^{13}C NMR of L_0 (C_6D_6, 101 MHz, 298 K).

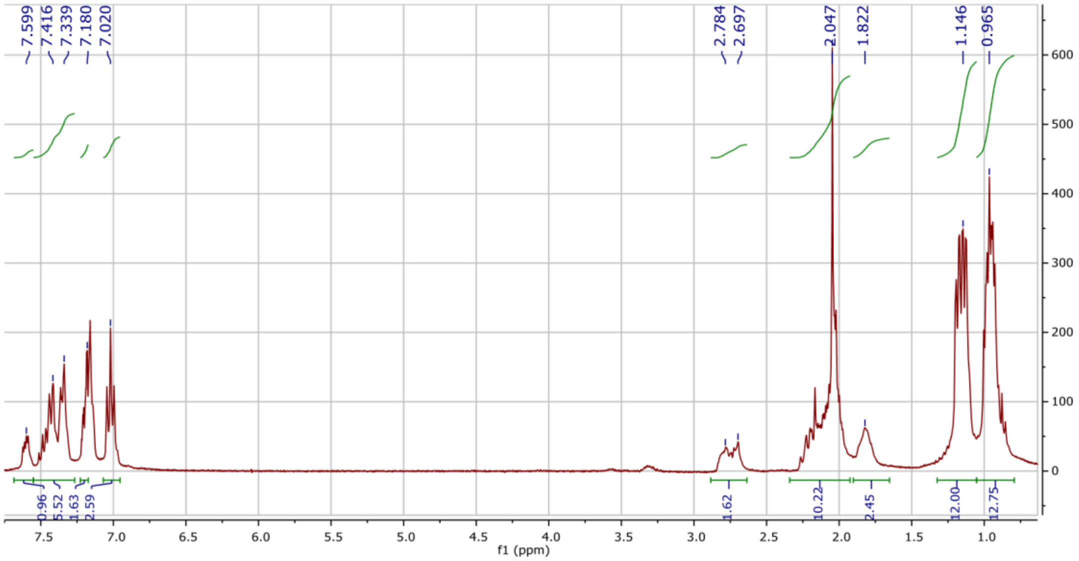

Figure S9. ^{1}H NMR of **L$_1$** (C_6D_6, 300 MHz, 298 K).

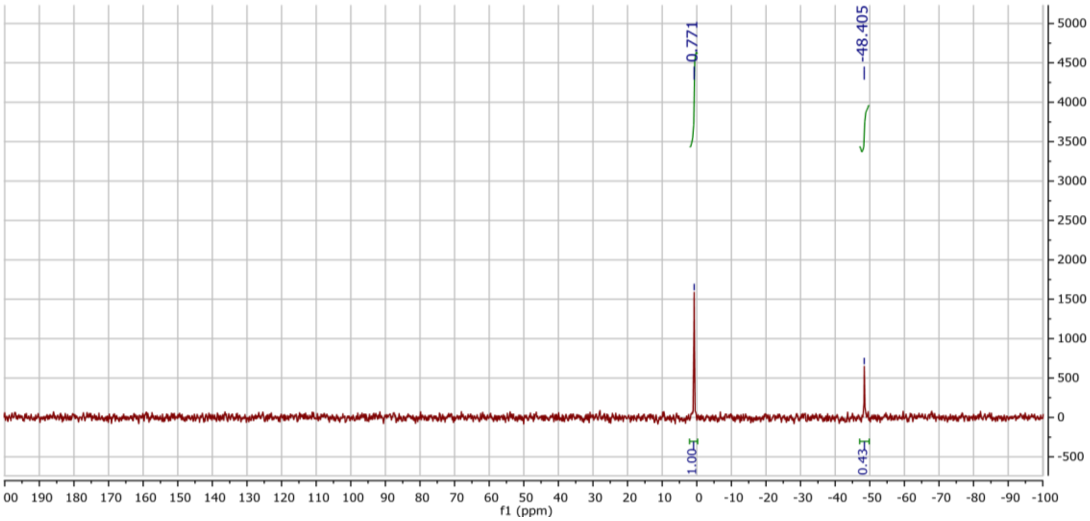

Figure S10. ^{31}P NMR of **L$_1$** (C_6D_6, 121 MHz, 298 K).

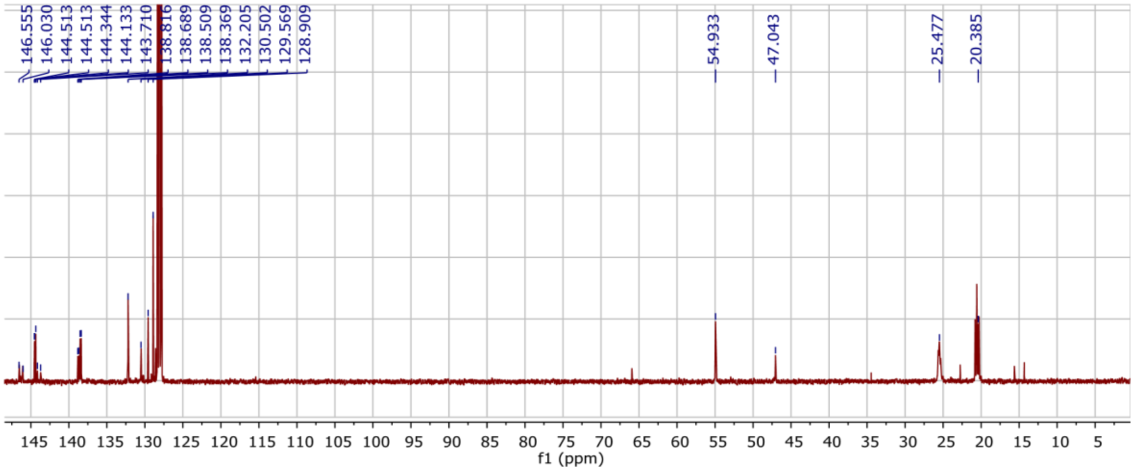

Figure S11. ^{13}C NMR of **L₁** (C_6D_6, 75 MHz, 298 K).

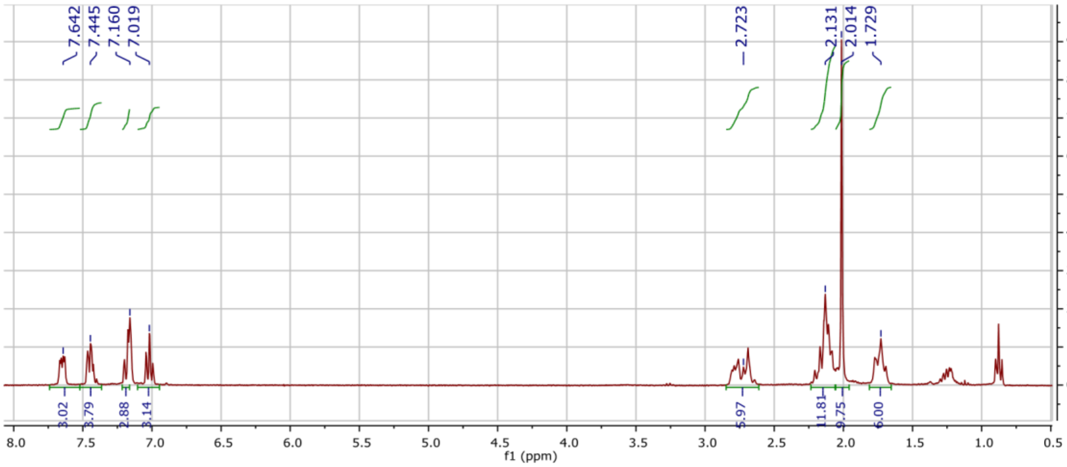

Figure S12. ^{1}H NMR of **L₂** (C_6D_6, 300 MHz, 298 K).

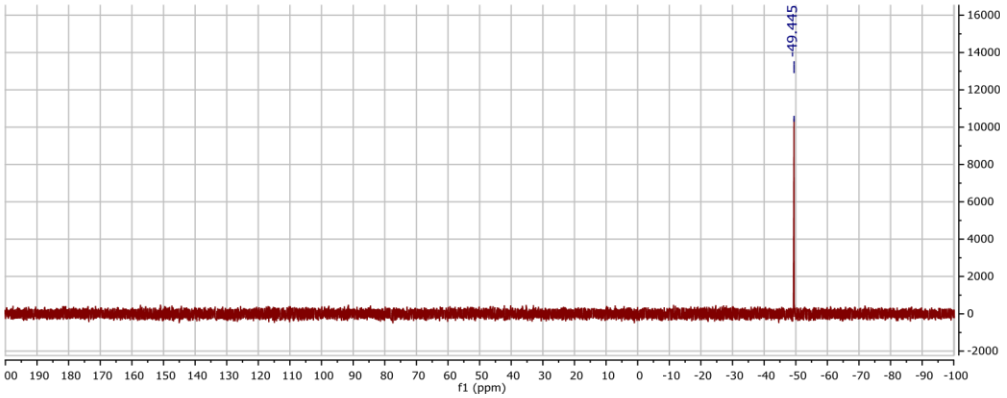

Figure S13. ^{31}P NMR of **L₂** (C_6D_6, 121 MHz, 298 K).

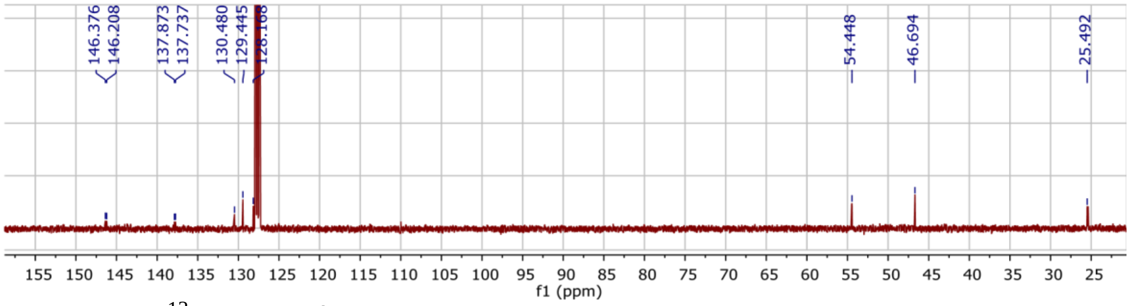

Figure S14. ^{13}C NMR of **L$_2$** (C_6D_6, 75 MHz, 298 K).

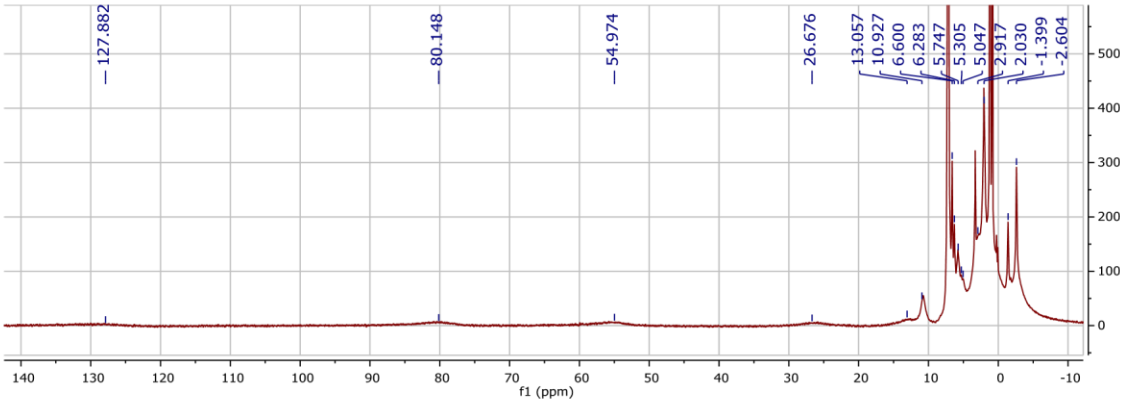

Figure S15. ^{1}H NMR of **1** (C_6D_6, 300 MHz, 298 K).

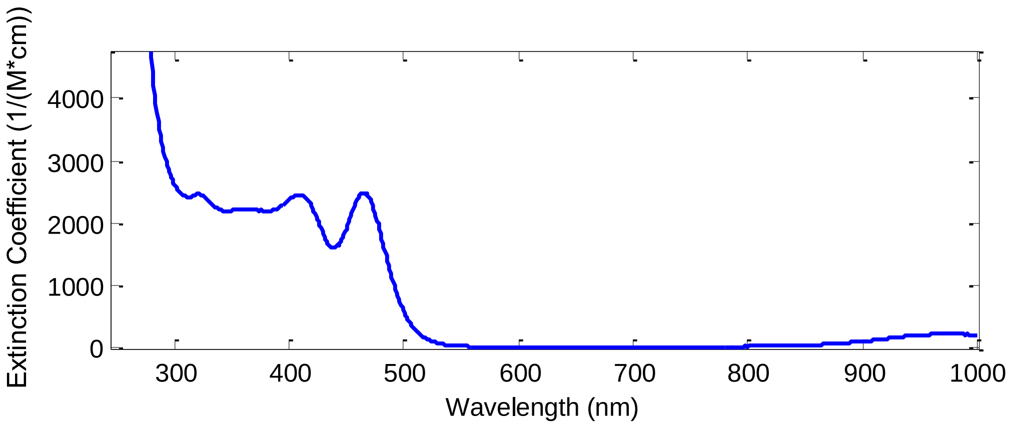

Figure S16. UV-vis spectrum of **1** in THF.

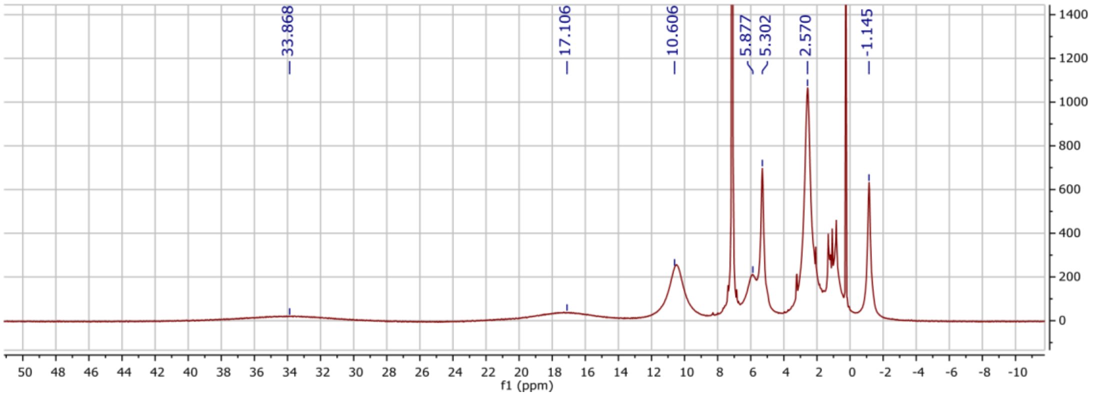

Figure S17. ^{1}H NMR of **1’** (C_6D_6, 300 MHz, 298 K).

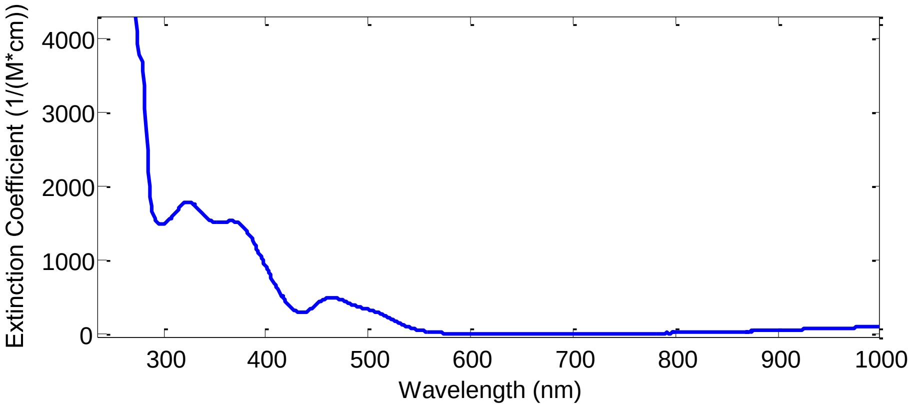

Figure S18. UV-vis spectrum of **1’** in THF.

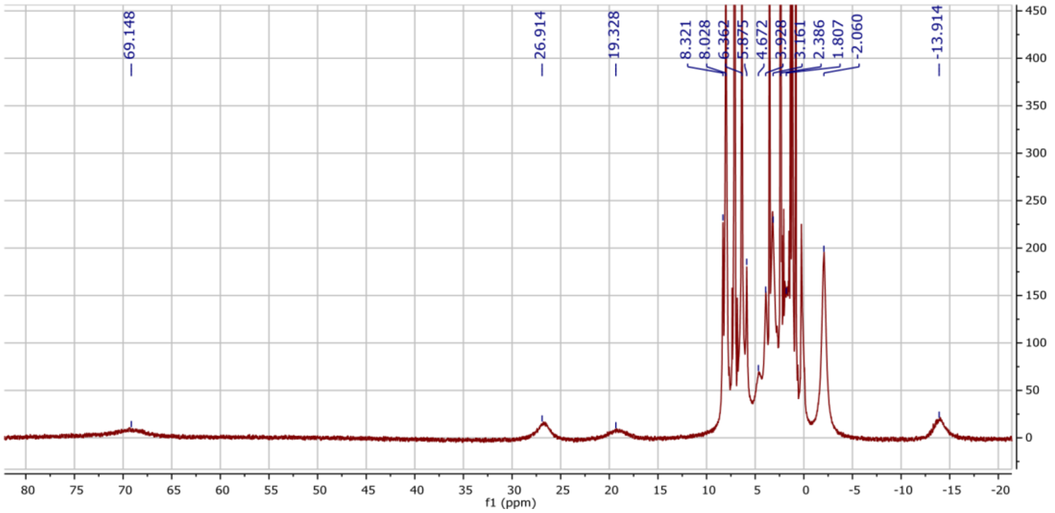

Figure S19. ^{1}H NMR of **2** (C_6D_6, 300 MHz, 298K).

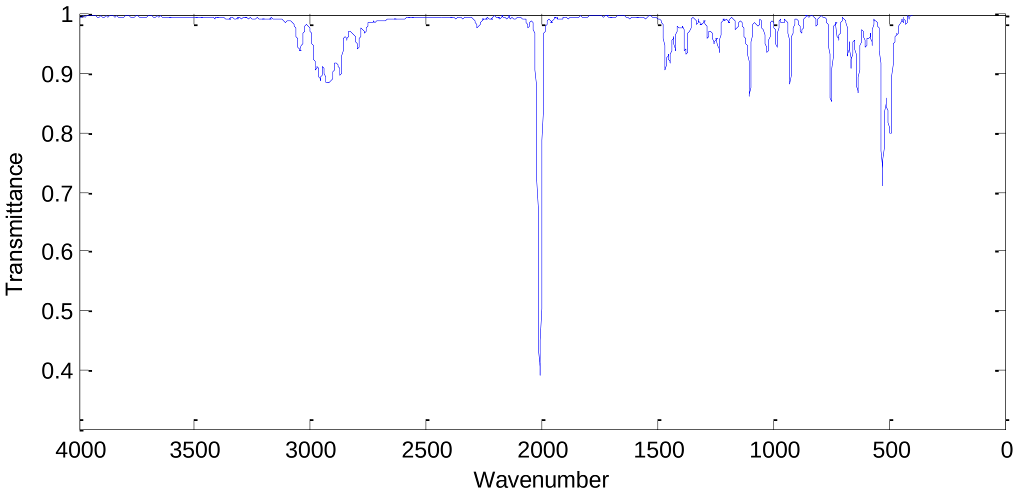

Figure S20. IR spectrum of **2** (thin film deposited from benzene).

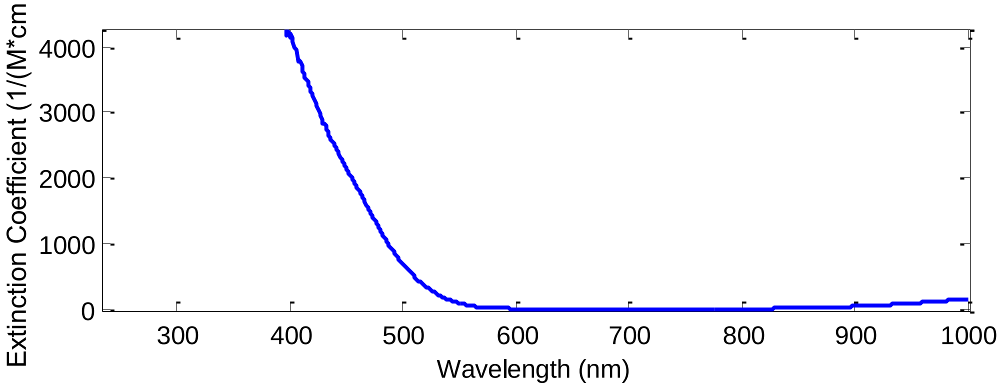

Figure S21. UV-vis spectrum of **2** in THF.

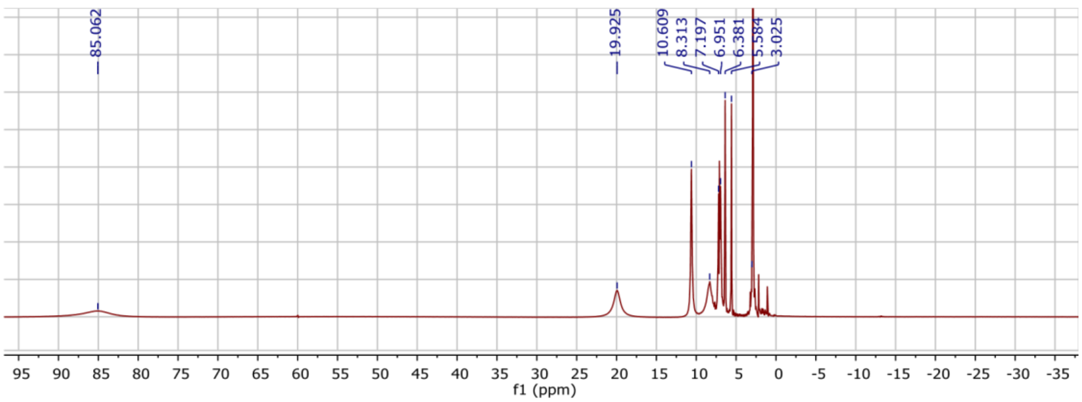

Figure S22. ^{1}H NMR of **2'** (C_6D_6, 300 MHz, 298 K).

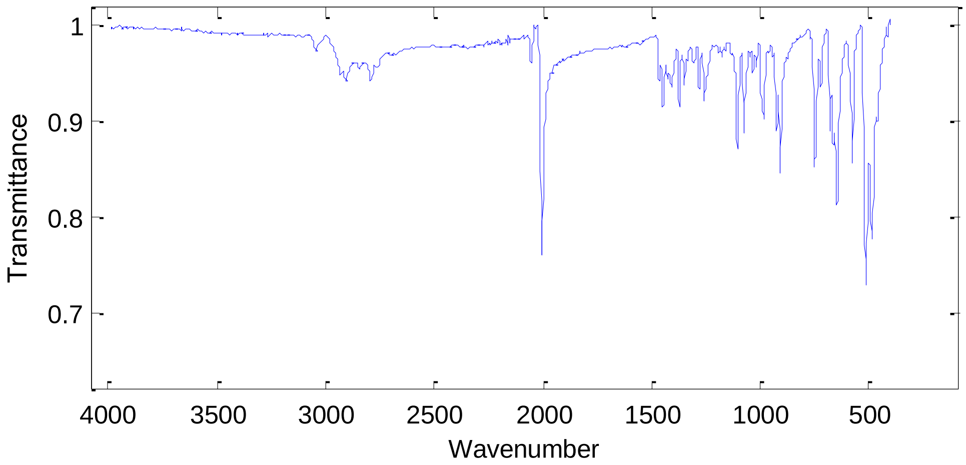

Figure S23. IR spectrum of **2’**(thin film deposited from benzene).

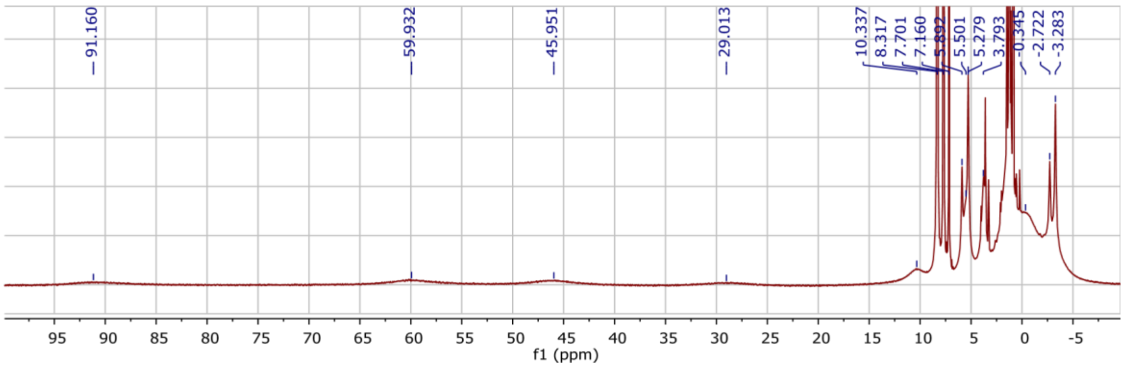

Figure S24. ^{1}H NMR of **3** (5:1 C_6D_6:d_8-THF, 300 MHz, 298 K).

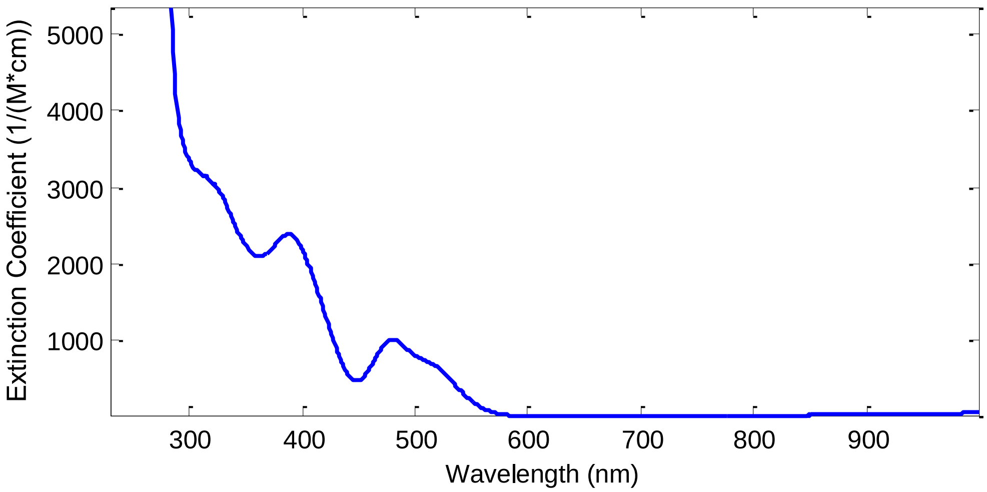

Figure S25. UV-vis spectrum of **3** in THF.

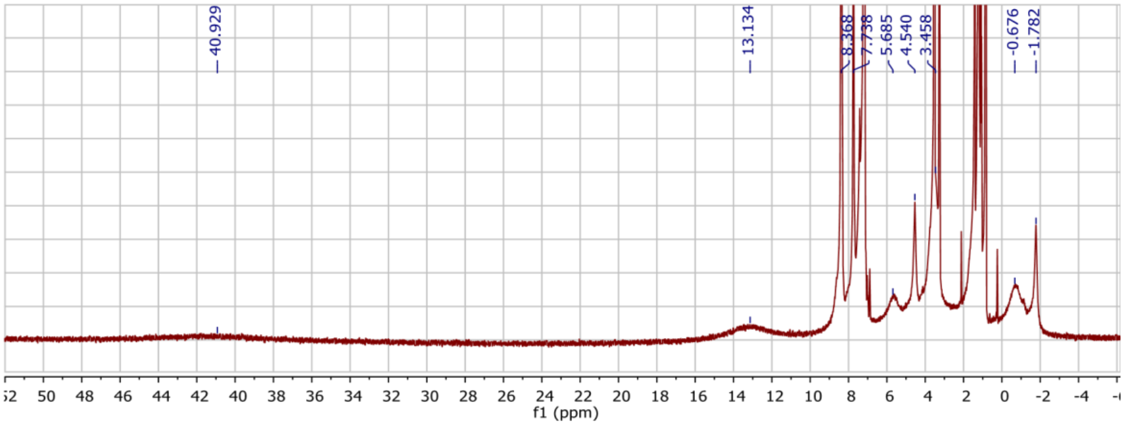

Figure S26. ^{1}H NMR of **3’** (5:1 C_6D_6:d_8-THF, 300 MHz, 298 K).

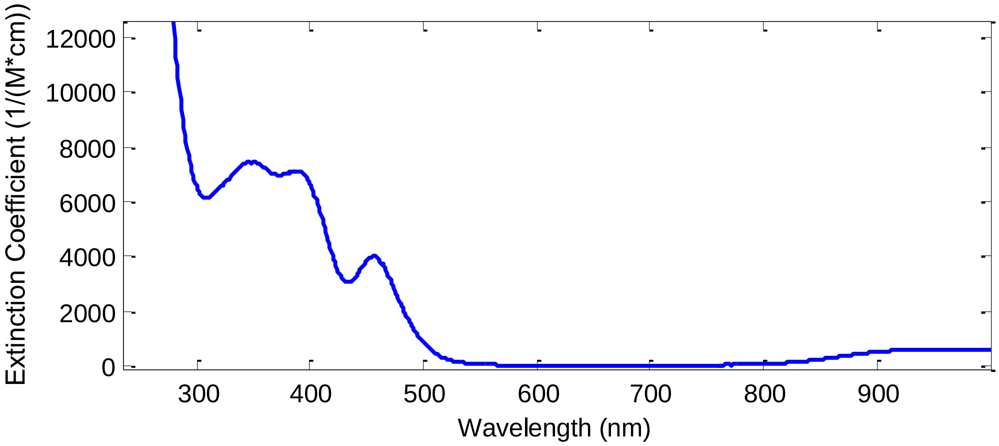

Figure S27. UV-vis spectrum of **3’** in THF.

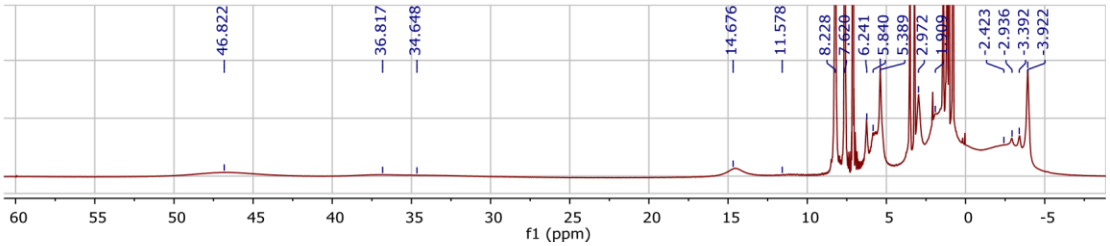

Figure S28. ^{1}H NMR of **4** (4:1 C_6D_6:d_8-THF, 300 MHz, 298 K).

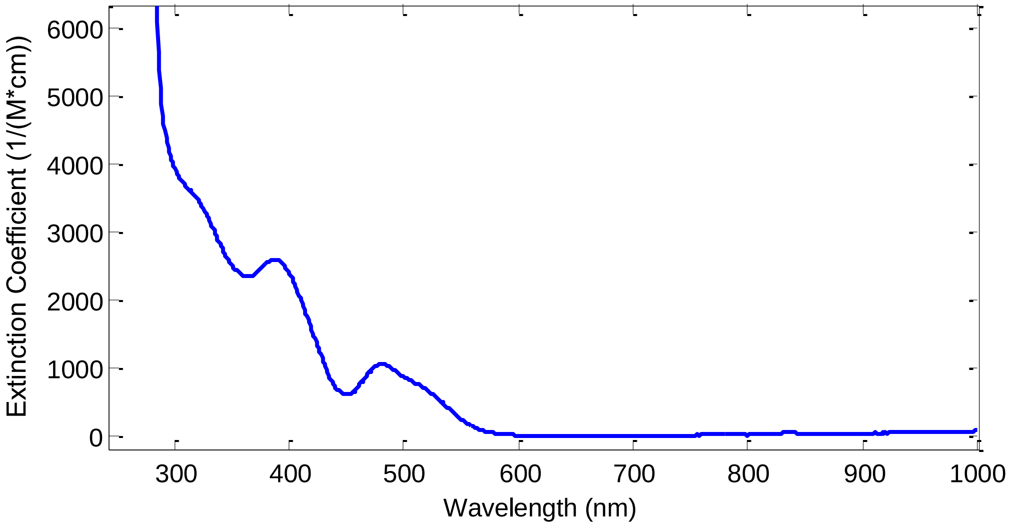

Figure S29. UV-vis spectrum of **4** in THF.

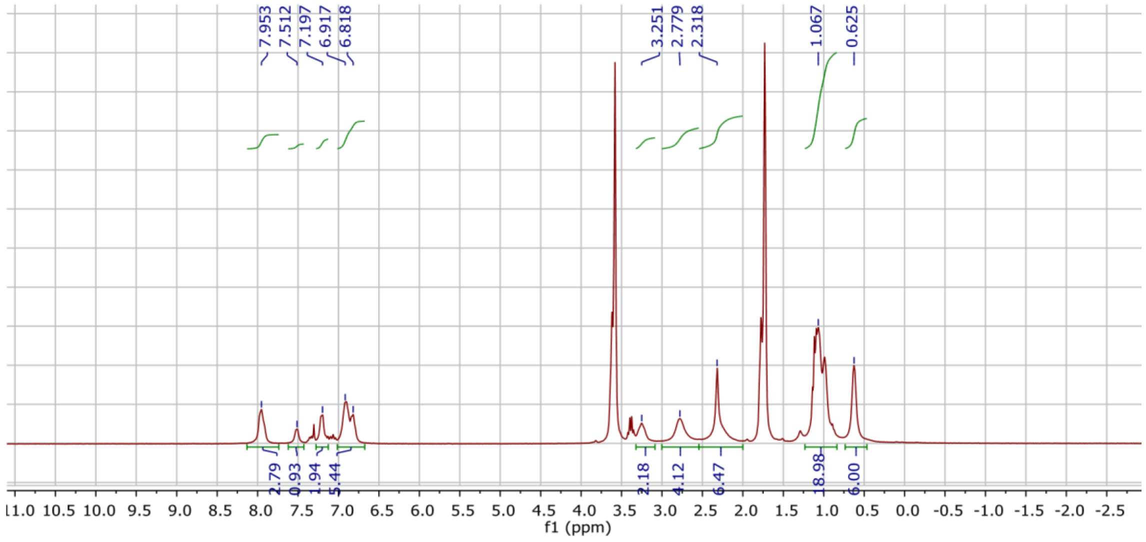

Figure S30. ^{1}H NMR of **6** in d_8-THF (300 MHz, 298 K).

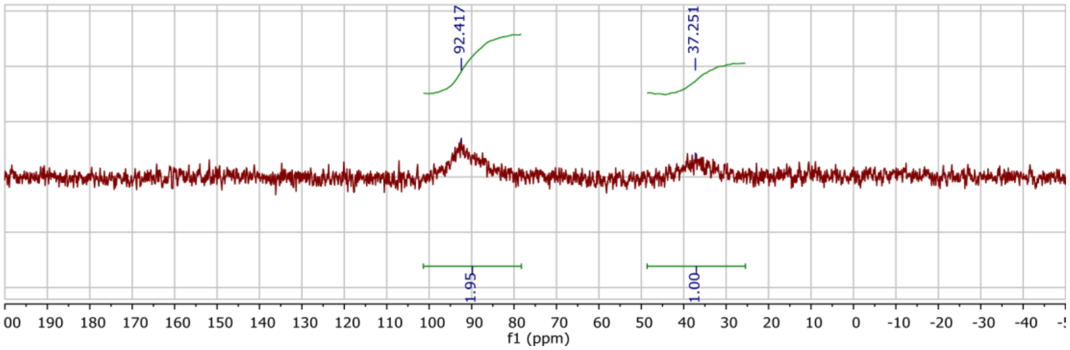

Figure S31. ^{31}P NMR of **6** in d_8-THF (121 MHz, 298 K).

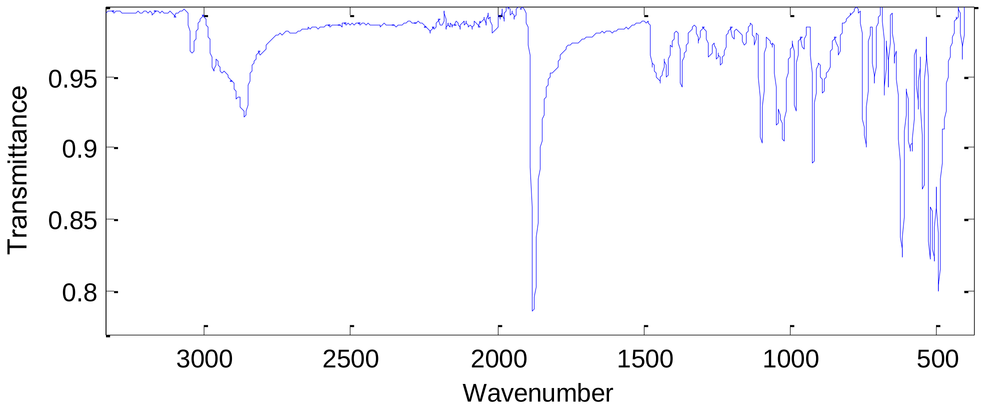

Figure S32. IR spectrum of **6** as a thin film deposited from THF.

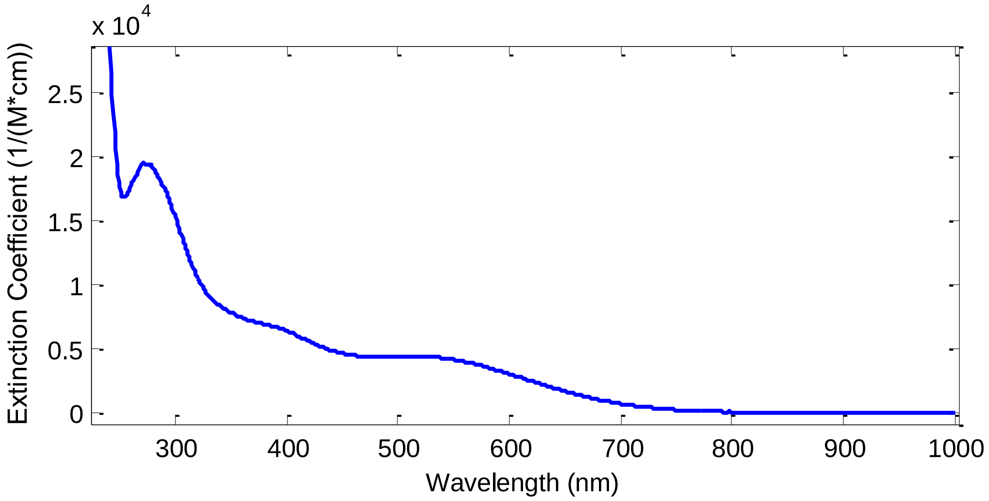

Figure S33. UV-vis spectrum of **6** in THF.

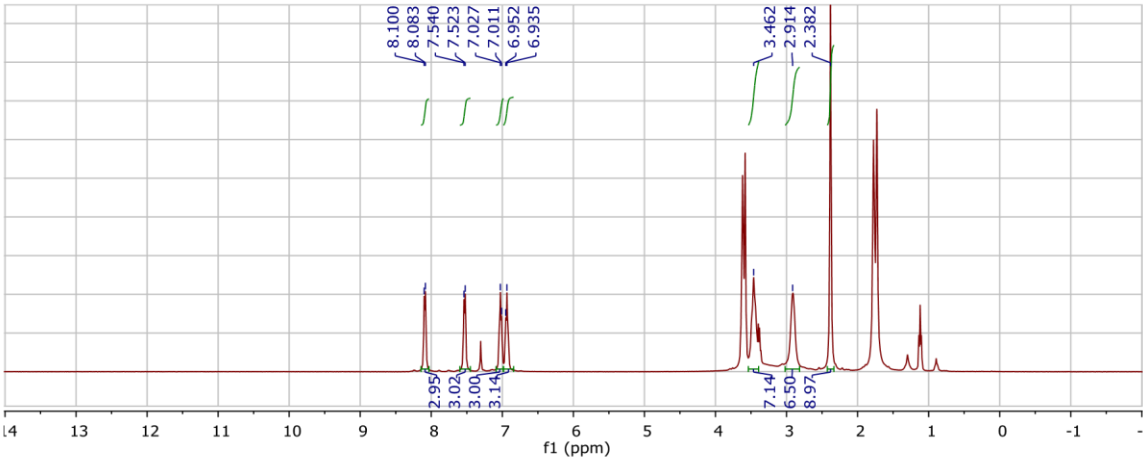

Figure S34. ^{1}H NMR spectrum of **6'** in d_8-THF (300 MHz, 298 K).

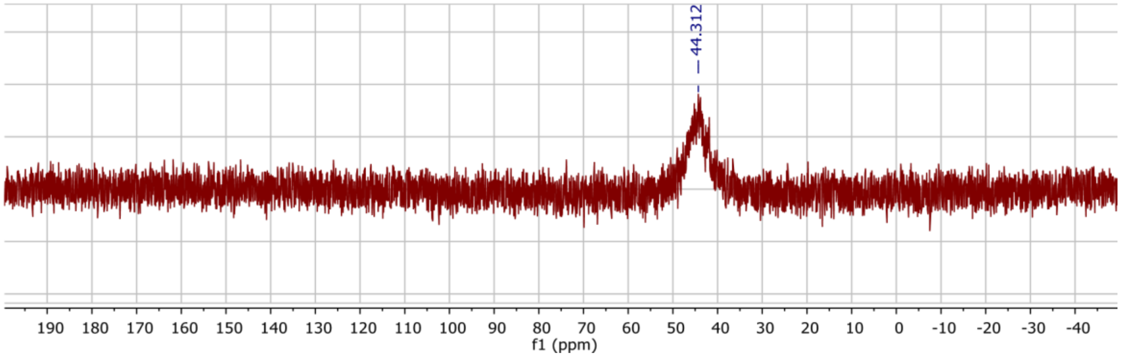

Figure S35. ^{31}P NMR spectrum of **6'** in d_8-THF (300 MHz, 298 K).

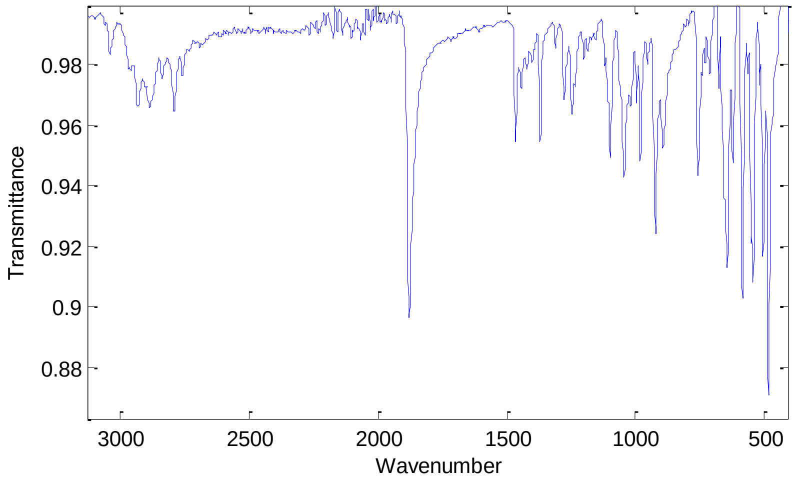

Figure S36. IR spectrum of **6'** as a thin film deposited from THF.

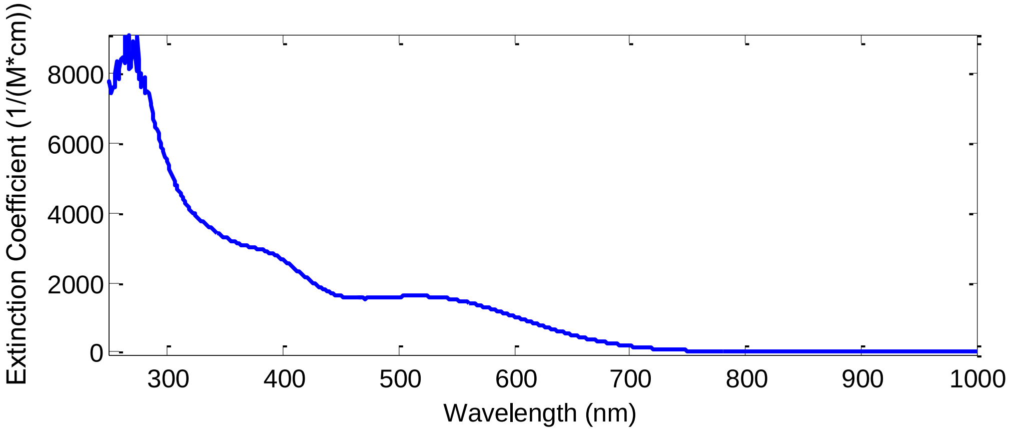

Figure S37. UV-vis spectrum of **6'** in THF.

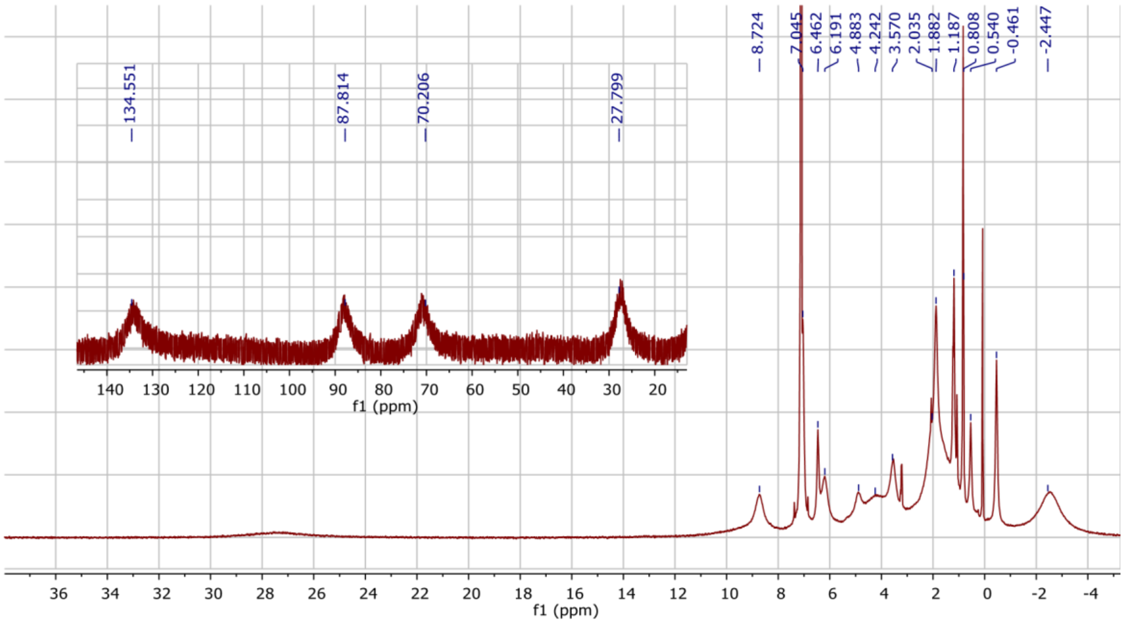

Figure S38. ^{1}H NMR of **5** in C_6D_6 (300 MHz, 298 K) with inset showing broad downfield resonances.

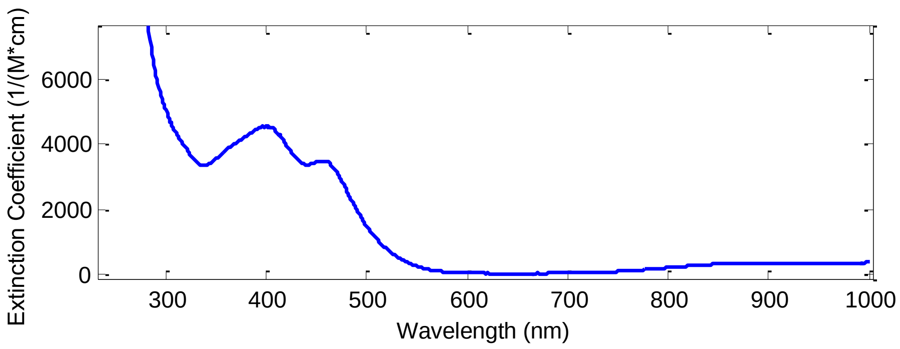

Figure S39. UV-vis spectrum of **5** in THF.

IV. Reactivity studies

Oxidation of [SiP^{iPr}$_2$P^{NMe}]FeNH$_2$ (5). A. 5 (25 mg, 0.037 mmol) was dissolved in Et_2O (3 mL) and cooled to -78 °C. A similarly cooled solution of FcBAr^F$_4$ (36.8 mg, 0.035 mmol) in Et_2O (3 mL) was added dropwise over two minutes. The orange solution turned a deeper red-orange color. The reaction mixture was allowed to warm to room temperature, concentrated to a volume of 1 mL, layered with pentane, and allowed to stand overnight. NMR analysis of the resulting orange crystalline precipitate confirmed the formation of {[SiP^{iPr}$_2$P^{NMe}]FeNH$_3$}{BAr^F$_4$}. **B.** (For EPR analysis) **5** (5.8 mg, 0.009 mmol) was dissolved in 2-MeTHF (0.5 mL) and frozen. A thawing solution of FcBAr^F$_4$ (8.1 mg, 0.008 mmol) in 2-MeTHF (0.5 mL) was added and the solution was allowed to thaw briefly, mixed, and transferred to a pre-cooled EPR tube. The reaction mixture was analyzed by EPR spectroscopy.

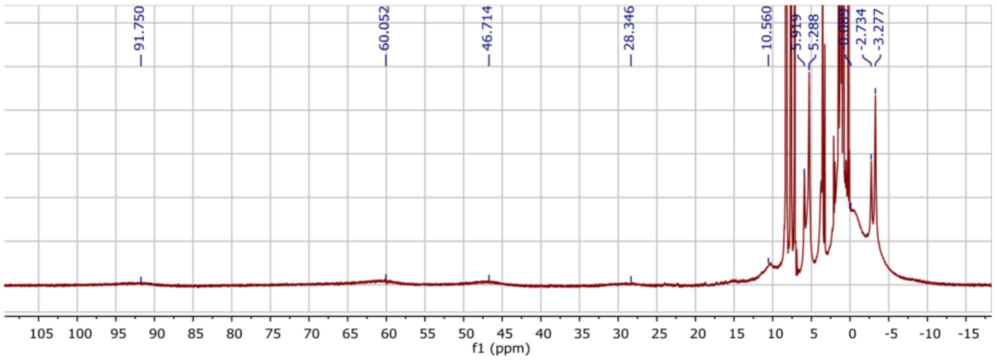

Figure S40. ^1H NMR (4:1 C_6D_6:d_8-THF, 300 MHz, 298 K) of terminal oxidation product of **5**.

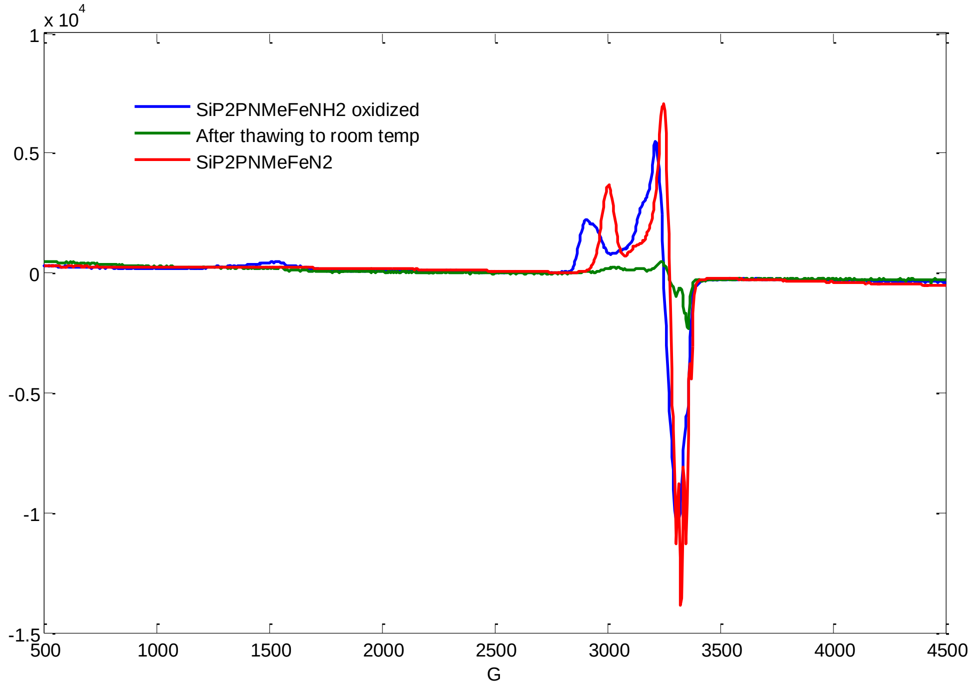

Figure S41. EPR spectrum of oxidation of **5** in thawing 2-MeTHF (blue), and after warming to room temperature (green). The EPR spectrum of **2** is also shown (red).

Attempted NH_3 production from {[SiP$_2$P^{NMe}]FeN$_2$}{Na(THF)$_2$} (6). {[SiP$_2$P^{NMe}]FeN$_2$}{Na(THF)$_2$} (1.6 mg, 0.0019 mmol) was dissolved in Et_2O (1 mL) and cooled to -78 ^oC. Similarly cooled solutions of KC_8 (15 mg, 55 eq, in 1 mL Et_2O) and $HBAr_4^F.2Et_2O$ (93 mg, 46 eq, in 1 mL Et_2O) were added rapidly and sequentially. The resulting reaction mixture was allowed to stir at low temperature for 2 hours before being allowed to warm to room temperature for one hour. The reaction mixture was then analyzed for ammonia production; no detectable ammonia was present.

Attempted NH_3 production from 6'. 6' (3.2 mg, 0.0035 mmol) was dissolved in Et_2O (1 mL) and cooled to -78 ^oC. Similarly cooled solutions of KC_8 (40 mg, 80 eq, in 1 mL Et_2O) and

$HBAr_4^F.2Et_2O$ (111 mg, 31 eq, in 1 mL Et_2O) were added rapidly and sequentially. The resulting reaction mixture was allowed to stir at low temperature for 2 hours before being allowed to warm to room temperature for one hour. The reaction mixture was then analyzed for ammonia production; no detectable ammonia was present.

Stoichiometric protonations of {[$SiP^{iPr}{}_2P^{NMe}$]FeN_2}{$Na(THF)_2$} (6). (a) {[$SiP^{iPr}{}_2P^{NMe}$]FeN_2}{$Na(THF)_2$} (54 mg, 0.063 mmol) was dissolved in Et_2O (2 mL) and cooled to -78 ^oC. A similarly cooled solution of $HBAr_4^F.2Et_2O$ (63 mg, 0.062 mmol) in 2 mL Et_2O was added in one portion with stirring, resulting in an immediate color change to yellow. The reaction was stirred at low temperature for 30 minutes and then allowed to warm to room temperature for one hour before concentrating to dryness. The resulting yellow residue was analyzed by NMR, which was consistent with formation of an iron hydride ([$SiP^{iPr}{}_2P^{NMe}$]$Fe(N_2)(H)$) as the main product. The NMR analysis demonstrates the *trans* disposition of the hydride ligand relative to the amine-bearing ligand arm, which is further confirmed crystallographically. X-ray quality crystals were grown by slow evaporation of a concentrated pentane solution of the crude reaction product. (b) {[$SiP^{iPr}{}_2P^{NMe}$]FeN_2}{$Na(THF)_2$} (10 mg, 0.012 mmol) was dissolved in Et_2O (2 mL) and cooled to -78 ^oC. A similarly cooled solution of [$HNEt^iPr_2$][BAr_4^F] (12.8 mg, 0.012 mmol) in 2 mL Et_2O was added in one portion with stirring, resulting in an immediate color change to yellow. The reaction was stirred at low temperature for 30 minutes and then allowed to warm to room temperature for one hour before concentrating to dryness. The resulting yellow residue was analyzed by NMR, which was again consistent with formation of an iron hydride ([$SiP^{iPr}{}_2P^{NMe}$]$Fe(N_2)(H)$) as the main product.

Stoichiometric protonation of 6'. 6' (5 mg, 0.0054 mmol) was dissolved in THF (1 mL) and cooled to -78 ^oC. A similarly cooled solution of $HBAr_4^F.2Et_2O$ (6.5 mg, 1.2 equiv.) in Et_2O (1 mL)

was added in one portion and the reaction mixture, which immediately turned yellow, was stirred at low temperature for one hour before being allowed to warm to room temperature. The yellow residue was then analyzed by NMR, which showed that the primary product is an iron hydride, $[SiP_3^{NMe}]Fe(N_2)(H)$. No oxidation product, $[SiP_3^{NMe}]FeN_2$, could be detected.

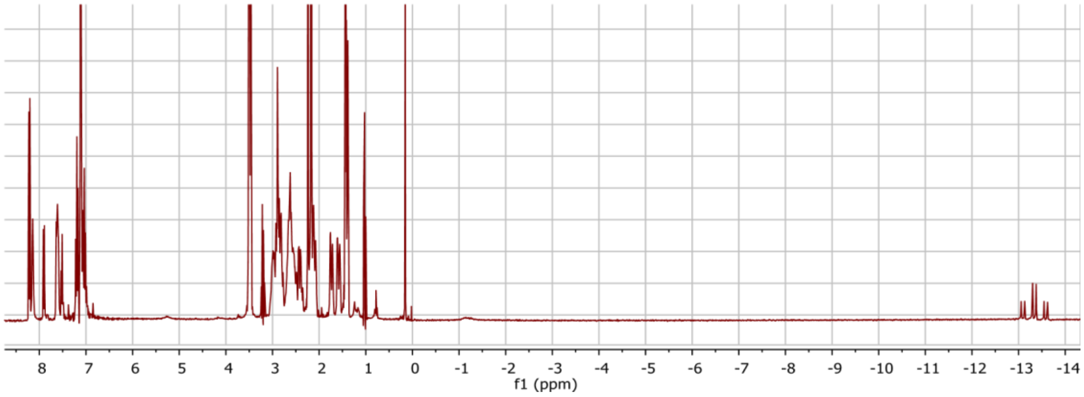

Figure S42. ^{1}H NMR (C_6D_6, 300 MHz, 298 K) of the protonation reaction of **6'** with one equivalent of $HBAr^F_4{\cdot}2Et_2O$.

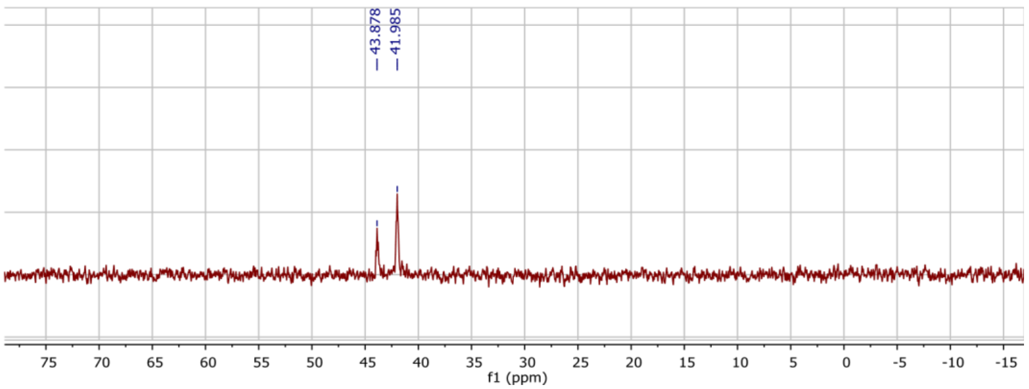

Figure S43. ^{31}P NMR (C_6D_6, 121 MHz, 298 K) of the protonation reaction of **6'** with one equivalent of $HBAr^F_4{\cdot}2Et_2O$.

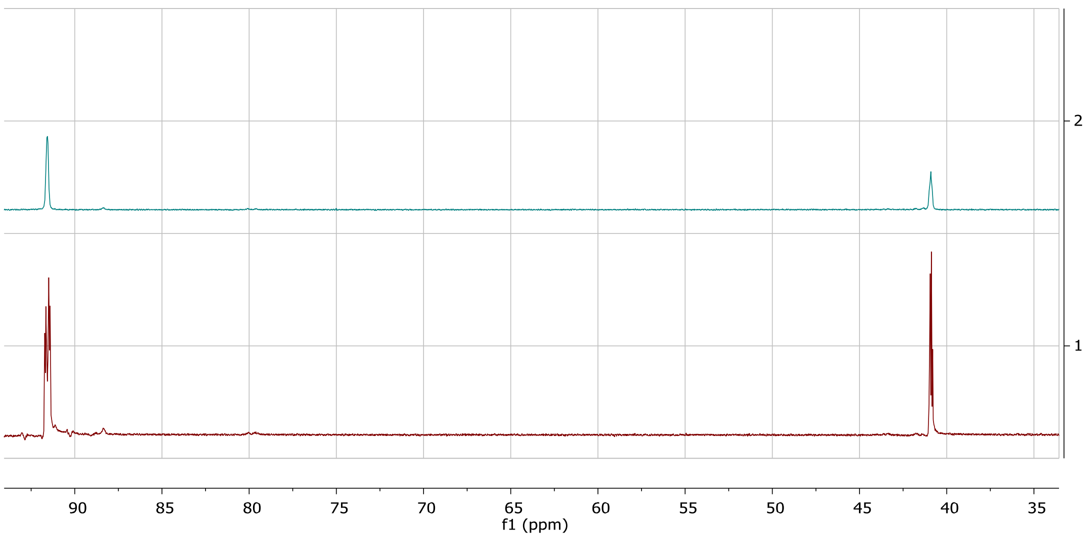

Figure S44. $^{31}P\{1H\}$ (top) and ^{31}P NMR spectra of the reaction mixture from protonation of **6** with one equivalent of $HBAr^{F}_{4}{\cdot}2Et_{2}O$ (C_6D_6, 298 K, 121 MHz).

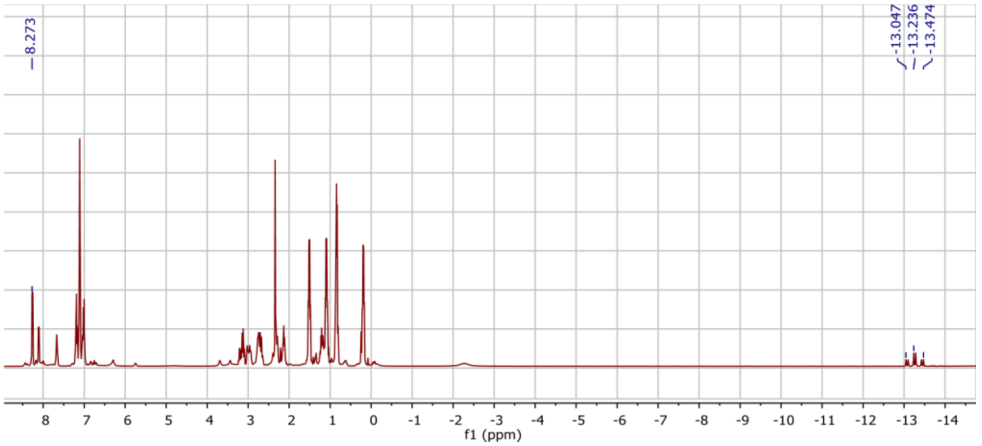

Figure S45. ^{1}H NMR spectrum of the reaction mixture from protonation of **6** with one equivalent of $HBAr^{F}_{4}{\cdot}2Et_{2}O$ (C_6D_6, 298 K, 300 MHz).

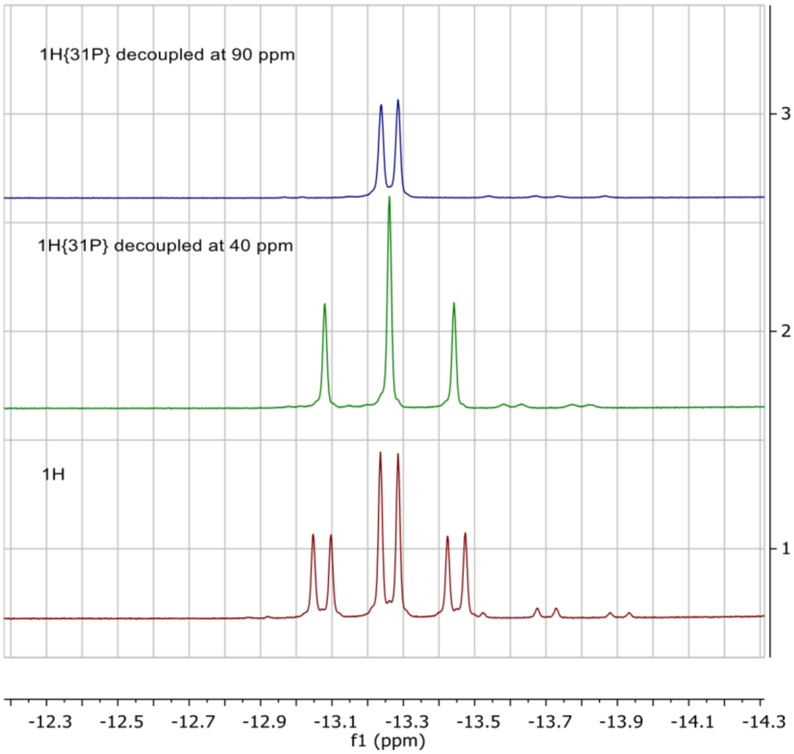

Figure S46. Close up on the Fe-H peak in the ^{1}H NMR spectrum, showing non-decoupled and ^{31}P-decoupled (decoupler set at 90 ppm and 40 ppm) spectra from protonation of **6** with one equivalent of $HBAr^{F}_{4}{\cdot}2Et_{2}O$ (C_6D_6, 298 K, 300 MHz).

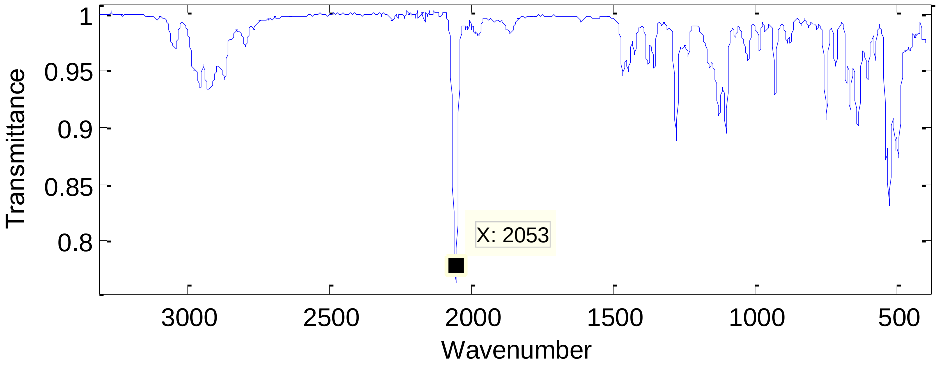

Figure S47. IR spectrum of the reaction mixture from protonation of **6** with one equivalent of $HBAr^{F}_{4}{\cdot}2Et_{2}O$ (thin film deposited from benzene).

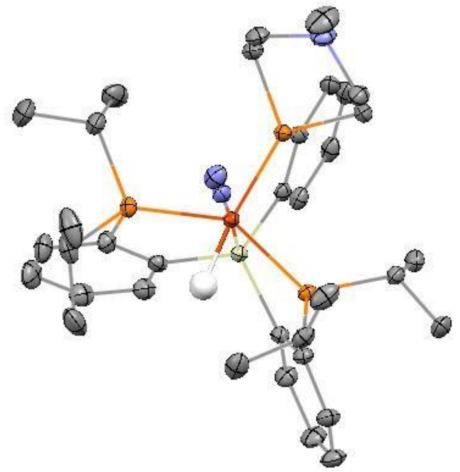

Figure S48. Structure of the $[SiP^{iPr}{}_2P^{NMe}]Fe(N_2)(H)$ product of the above reaction. Thermal ellipsoids are shown at 50% probability and hydrogen atoms (other than the hydride) are omitted for clarity. The metal-bound hydrogen atom was located crystallographically and the wide P-FeP angle *trans* to the unique ligand arm confirms its placement.

Protonation of {[SiP^{iPr}$_3$]FeN$_2$}{Na(THF)$_3$}. (a) $\{[SiP^{iPr}{}_3]FeN_2\}\{Na(THF)_3\}$ (20 mg, 0.022 mmol) was dissolved in Et_2O (2 mL) and cooled to -78 ^oC. A similarly cooled solution of $[HNEt^iPr_2][BAr_4^F]$ (21.4 mg, 0.022 mmol) in Et_2O (2 mL) was added in one portion, and the reaction mixture was stirred at low temperature for 2 hours, resulting in a color change to yelloworange. It was then warmed to room temperature for 1 hour before concentrating to dryness and analyzing the product by NMR and IR, which showed that it was solely the previously reported oxidation product, $[SiP^{iPr}{}_3]FeN_2$.[6] (b) $\{[SiP^{iPr}{}_3]FeN_2\}\{Na(THF)_3\}$ (10 mg, 0.011 mmol) was dissolved in Et_2O (2 mL) and cooled to -78 ^oC. A similarly cooled solution of $HBAr^F{}_4{\cdot}2Et_2O$ (12 mg, 0.011 mmol) in Et_2O (2 mL) was added in one portion, and the reaction mixture was stirred at low temperature for 2 hours, resulting in a color change to yellow-orange. It was then warmed to

room temperature for 1 hour before concentrating to dryness and analyzing the product by NMR and IR, which showed that it was solely the oxidation product, [SiP$_3^{iPr}$]FeN$_2$.

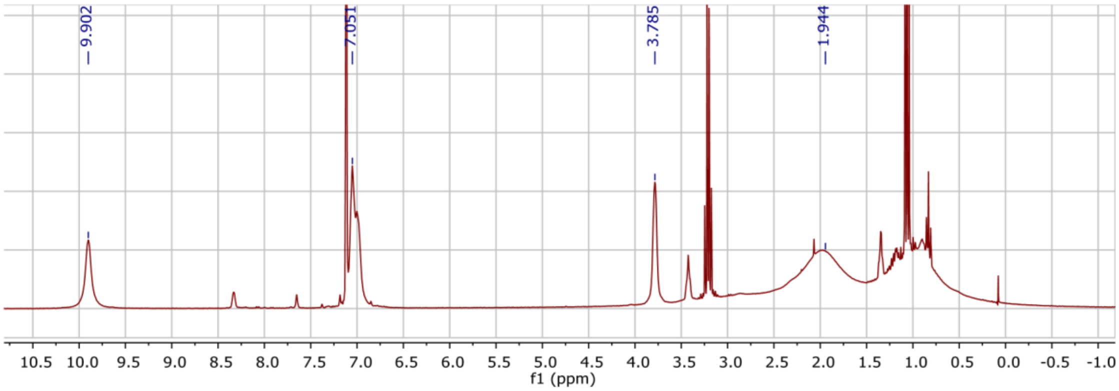

Figure S49. ^{1}H NMR (C_6D_6, 300 MHz, 298 K) from protonation of {[SiP$^{iPr}_3$]FeN$_2$}{Na(THF)$_3$} with one equivalent of HBAr$^F_4$·2Et$_2$O (thin film deposited from benzene).

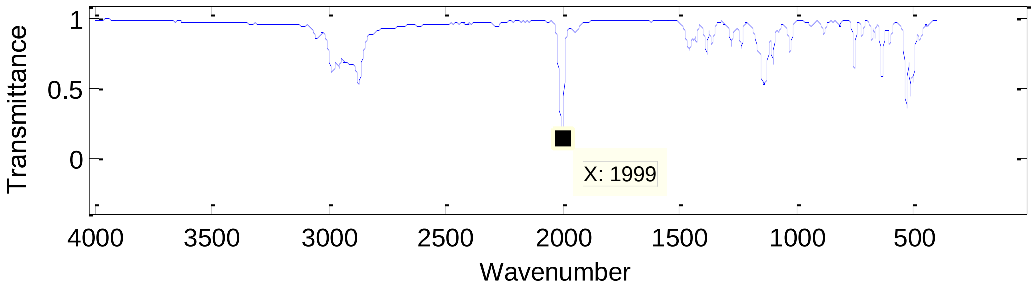

Figure S50. IR spectrum from protonation of {[SiP$^{iPr}_3$]FeN$_2$}{Na(THF)$_3$} with one equivalent of HBAr$^F_4$·2Et$_2$O (thin film deposited from benzene).

Ammonia quantification. Reaction mixtures were analyzed for the presence of ammonia according to the following procedure. Upon completion of the reaction, volatiles were vactransferred onto an ethereal solution of HCl (4 mL, 2 M). The solid residue was treated with a solution of KO^{t}Bu in THF and again vac transferred onto the HCl solution. The resulting ammonium chloride was analyzed by the indophenol method as previously reported.[8]

Decomposition of {[SiP$^{iPr}{}_2$P^{NMe}]FeN$_2$H$_4$}{BAr$^F{}_4$} (4) and {[SiP$^{iPr}{}_3$]FeN$_2$H$_4$}{BAr$^F{}_4$}. The decomposition of a 0.02 M solution of **4** or {[SiP$^{iPr}{}_3$]FeN$_2$H$_4$}{BAr$^F{}_4$} in 5:1 C_6D_6:d_8-THF at 60 °C was monitored by NMR. The decomposition of **4** was complete within four hours, while the decomposition of {[SiP$^{iPr}{}_3$]FeN$_2$H$_4$}{BAr$^F{}_4$} required >48 hours.

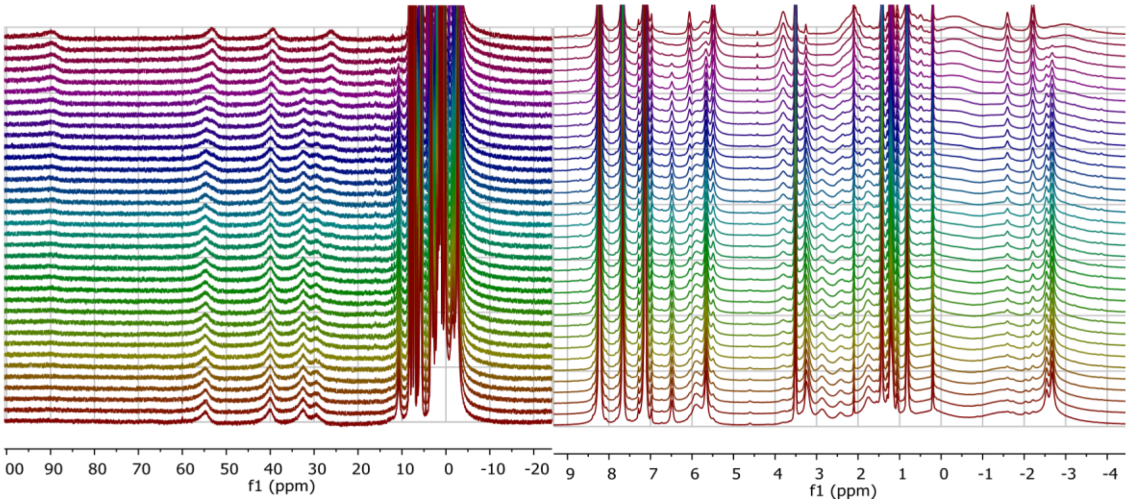

Figure S51. Arrayed ^{1}H NMR spectra (500 MHz, 333 K, 5:1 C_6D_6:d_8-THF) following decomposition of **4** (bottom) to **3** (top) over the course of four hours, showing full (left) and closeup (right) spectra. Scans were acquired 7 minutes apart.

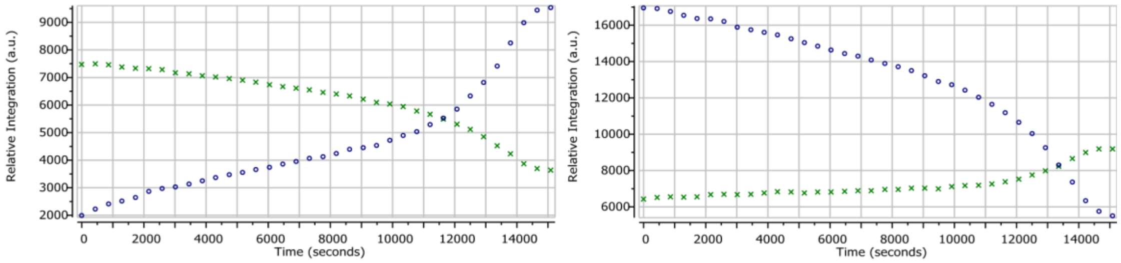

Figure S52. Relative integration of select peaks corresponding to **4** and **3** from the arrayed spectra shown in Fig. S51. Left graph shows the relative integration of the peak at 5.66 ppm corresponding to **4** (green) and the peak at 5.49 ppm corresponding to **3** (blue). Right graph shows the relative integration of the two peaks centered at -2.6 ppm corresponding to **4** (blue) and the peak at -2.25 ppm corresponding to **3** (green). Scans were acquired 7 minutes apart.

V. Computational details

All calculations were carried out using the Gaussian program suite.[9]

Computational analysis of conformer energies. In order to roughly approximate the difference in energy between ligand conformational isomers bearing the six-membered azaphosphine ring in a "chair-like" versus a "boat-like" conformation, DFT geometry optimizations and energy calculations were carried out on the uncoordinated ligand $HSiP^{Me}{}_2P^{NMe}$ (**L1'**), identical to **L1** except that the isopropyl groups have been truncated to methyls. The starting geometries for **L1'chair** and **L1'-boat** were obtained from the crystal structures of $[SiP^{iPr}{}_2P^{NMe}]FeNH_2$ (**5**) and $\{[SiP^{iPr}{}_2P^{NMe}]FeNH_3\}\{BAr^F{}_4\}$ (**3**), respectively, following removal of the $[FeNH_2]$ and $[FeNH_3]$ fragments, removal of the counterion, and addition of a proton bonded to silicon. Geometry optimizations followed by frequency calculations were carried out at the BP86/6-31g(d) level of theory.

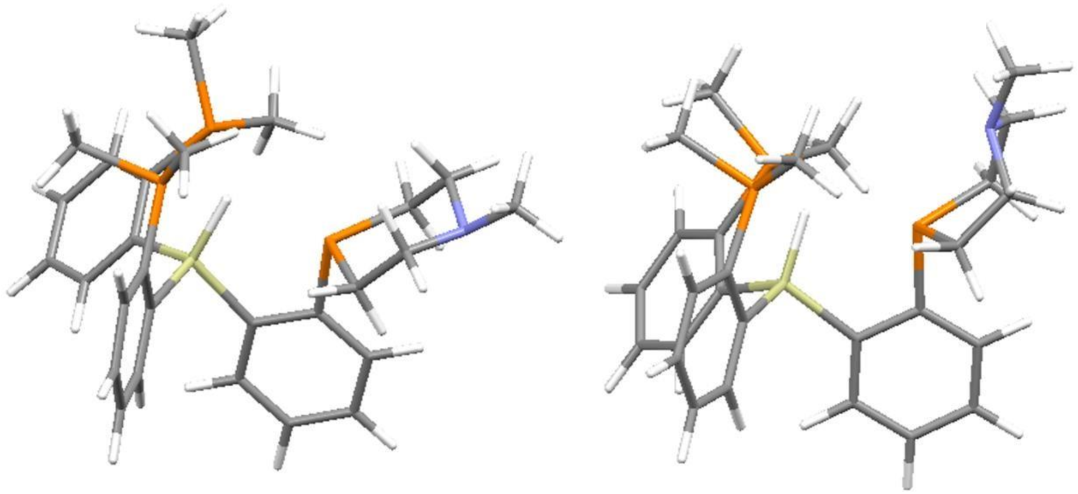

Figure S53. Optimized structures of (truncated) **L$_1$** in the chair (left) and boat (right) conformations.

The calculated ΔG(298K) between the two isomers is 4.1 kcal/mol, with the boat conformer lying higher in energy as expected.

Proton affinity calculations. Gas-phase proton affinities (-ΔH(298K)) were calculated for the protonation of the {[SiP$^{iPr}{}_2$P^{NMe}]FeN$_2^-$} model anion at three different locations—at the terminal nitrogen of the N_2 ligand, at the tertiary amine, and at the metal to give an iron hydride. Geometry optimizations and energy calculations were carried out for the starting material and products based on the crystallographically determined coordinates of {[SiP$^{iPr}{}_2$P^{NMe}]FeN$_2$}{Na(THF)$_2$} following removal of the cation and solvent and addition of a proton where applicable, or starting from the crystallographically determined coordinates of [SiP$^{iPr}{}_2$P^{NMe}]Fe(N$_2$)(H), at the BP86/6-31g(d) level of theory. All species were calculated as singlets. The calculations suggest that, consistent with the experimental results, protonation at the metal to give a hydride is the thermodynamically preferred product by a substantial margin, while, thermodynamically, protonation at the N_2 ligand is slightly favored over protonation of the tertiary amine.

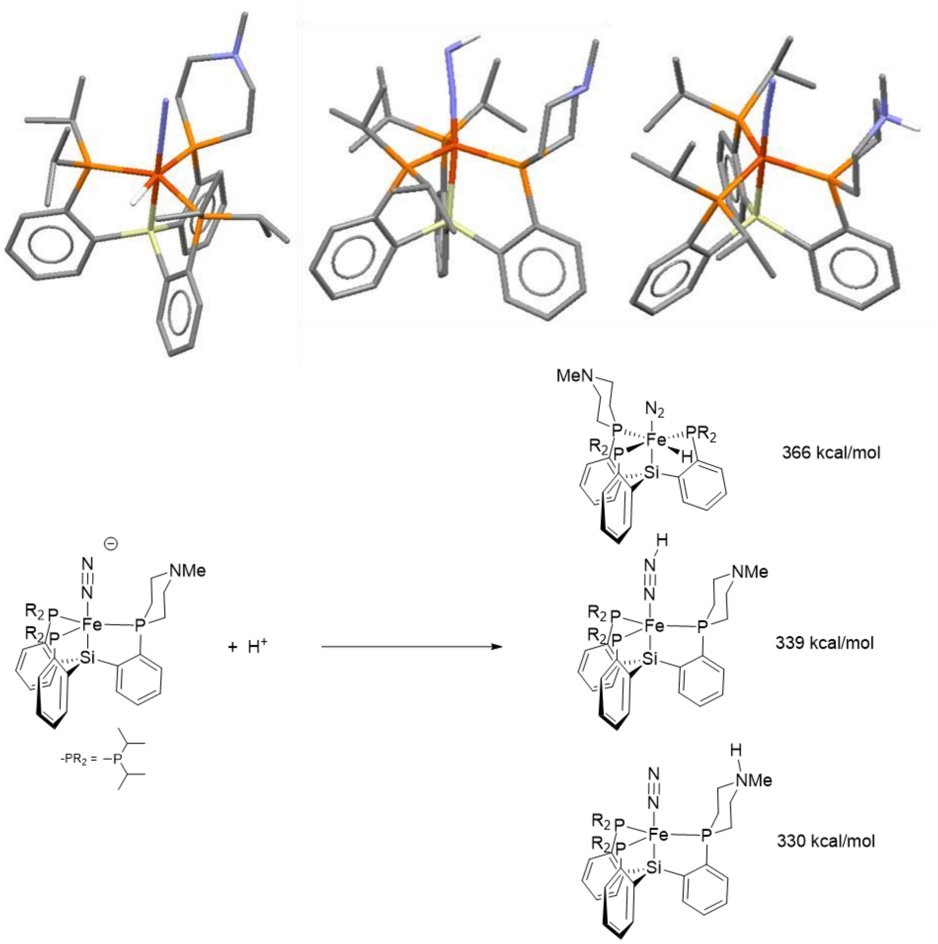

Figure S54. Calculated structures of different possible protonation products of **6**, and calculated proton affinities for each isomer.

VI. Crystallographic details

General. XRD studies were carried out at the Beckman Institute Crystallography Facility on either a Bruker Kappa Apex II diffractometer or a Bruker D8 Venture Kappa Duo Photon 100 CMOS instrument (Mo Kα or Cu Kα radiation). Structures were solved using SHELXS and refined against F^2 on all data by full-matrix least-squares with SHELXL.[10] The crystals were mounted on a wire loop or glass fiber under paratone oil. Methyl group hydrogen atoms not involved in disorder were placed at calculated positions starting from the point of maximum electron density. All other hydrogen atoms, except where otherwise noted, were placed at geometrically calculated positions and refined using a riding model. The isotropic displacement parameters of the hydrogen atoms were fixed at 1.2 (1.5 for methyl groups) times the U_{eq} of the atoms to which they are bonded. 1,2- and 1,3- rigid bond restraints were applied to all non-hydrogen atoms.

CCDC 1511362 (**1'**), 1511363 (**2'**), 1511369 (**3**), 1511371 (**3'**), 1511365 (**4**), 1511368 (**5**), 1511367 (**6**), 1511364 (**6'** at 100 K), 1511366 (**6'** at 200 K), and 1511370 ([$SiP^{iPr}_2P^{NMe}$]$Fe(N_2)(H)$) contain supplementary crystallographic data for this paper.

$[SiP_3^{NMe}]FeCl$ (1'). The asymmetric unit for this crystal contains two full molecules of the complex as well as two benzene molecules.

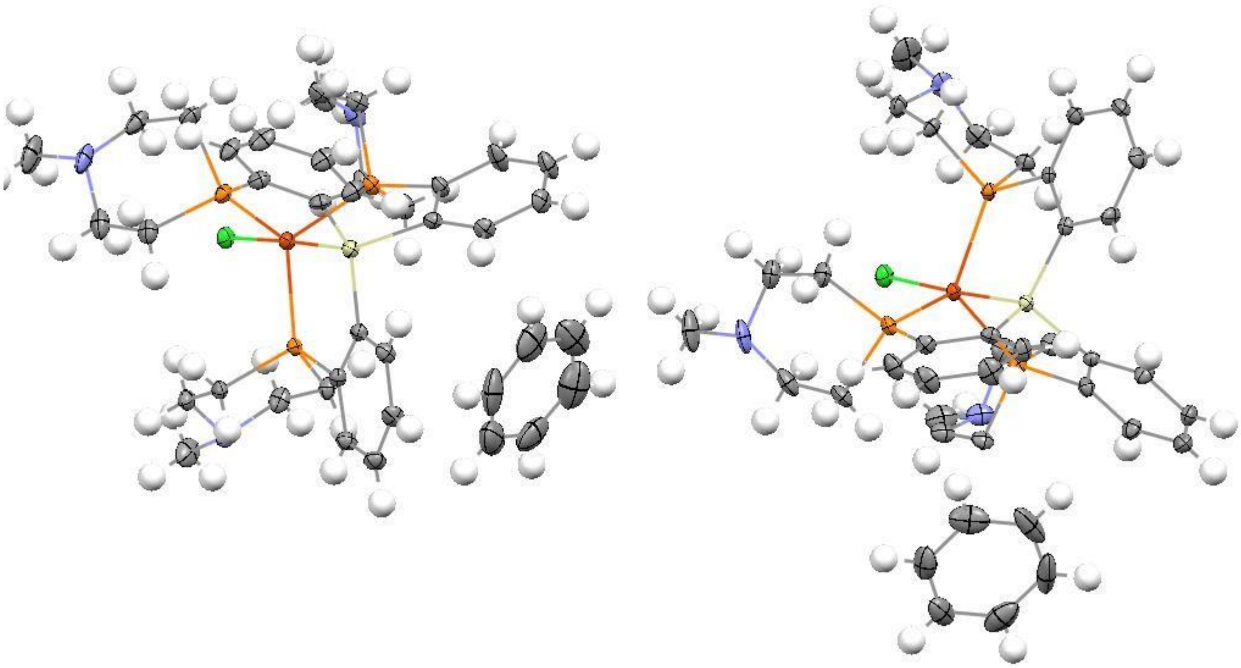

Figure S55. Full asymmetric unit of the structure of **1'**. Thermal ellipsoids shown at 50% probability.

[SiP$_3^{NMe}$]FeN$_2$ (2'). The asymmetric unit for this crystal contains two full molecules of the complex and no solvent molecules.

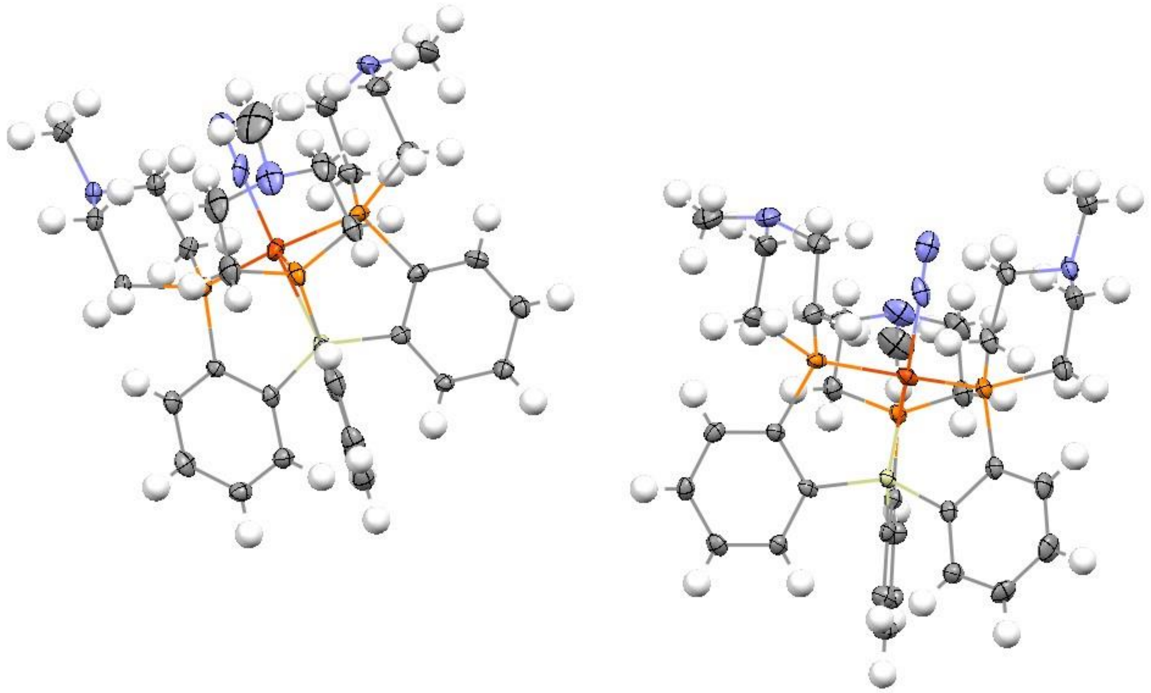

Figure S56. Full asymmetric unit of the structure of **2'**. Thermal ellipsoids shown at 50% probability.

{[SiP$^{iPr}_2$P^{NMe}]FeNH$_3$}{BAr$_4^F$} (3). The asymmetric unit for this crystal contains one full molecule (cation and BAr$^F_4$ anion) and 0.5 diethyl ether molecules (located on a two-fold special position). The N-bound hydrogen atoms were located in the density difference map and refined freely. Four isopropyl groups were modeled as disordered over two positions in 54:46, 58:42, 56:44, and 56:44 ratios. One phenyl group was also modeled with a 56:44 disorder. Additionally, three CF$_3$ groups on the BAr$^F_4$ anion were modeled as a rotational disorder over two positions in 52:48, 52:48, and 52:48 ratios. ISOR, SAME, and other geometric (AFIX 66) restraints were used to aid in modeling these disorders, along with EADP constraints where appropriate.

{[SiP^{iPr}$_2$P^{NMe}]FeN$_2$H$_4$}{BAr$_4$^{F}} (4). The asymmetric unit for this crystal contains one full molecule (cation and BAr^F$_4$ anion) and 1.5 diethyl ether molecules (one ether molecule is located on a two-fold special position). The N-bound hydrogen atoms were located in the density difference map and refined freely. One CF_3 group on the BAr^F$_4$ anion was modeled as an 80:20 rotational disorder.

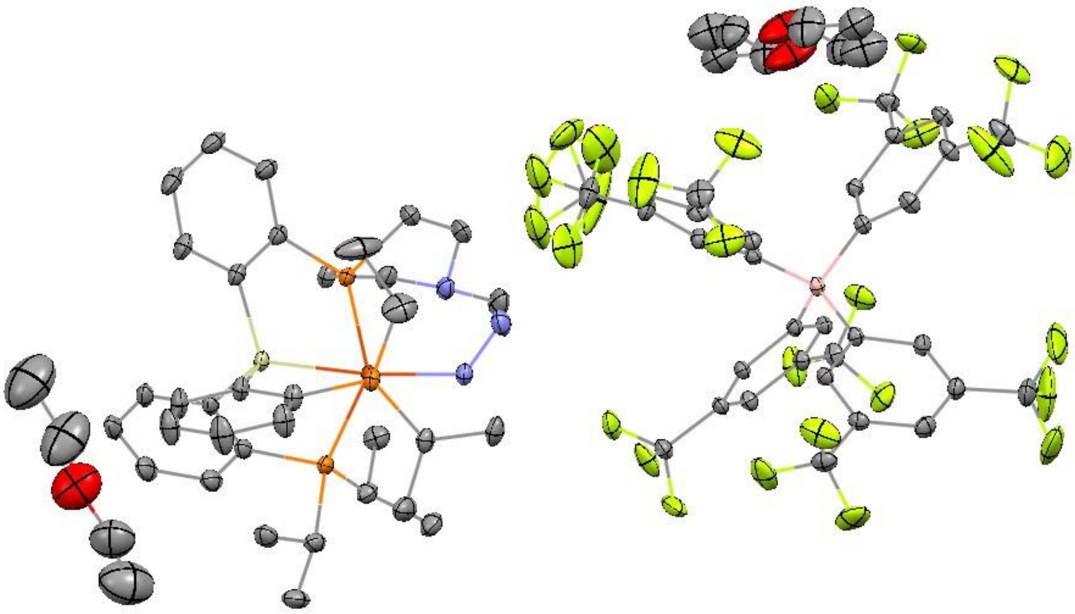

Figure S57. Full asymmetric unit of **4**. Thermal ellipsoids shown at 50% probability and hydrogen atoms omitted for clarity.

{[SiP$_3$^{NMe}]FeNH$_3$}{BAr$_4$^{F}} (3'). The asymmetric unit for this crystal contains two full cations of the complex, two BAr^F$_4$ counteranions, and two diethyl ether molecules. Five disagreeable reflections were omitted from the refinement. N-bound hydrogen atoms were located in the density difference map and refined isotropically and freely except that DFIX restraints of 1.01 Å were applied to two of the N-H bonds (one on each molecule). On one of the cations, two of the azaphosphine rings were modeled as disordered over two positions, one as a 66:34 disorder between boat and chair conformations, respectively, and one as a 74:26 disorder between two boat

conformers. In the BAr^F_4 counteranions, four CF_3 groups were modeled as disorders in 50:50, 82:18, 48:52, and 59:41 ratios. SAME and ISOR restraints were used to aid in modeling in these disorders; however, one A-level and four B-level alerts still persist for the U_{min}/U_{max} ratios of some of these fluorine atoms due to these disorders.

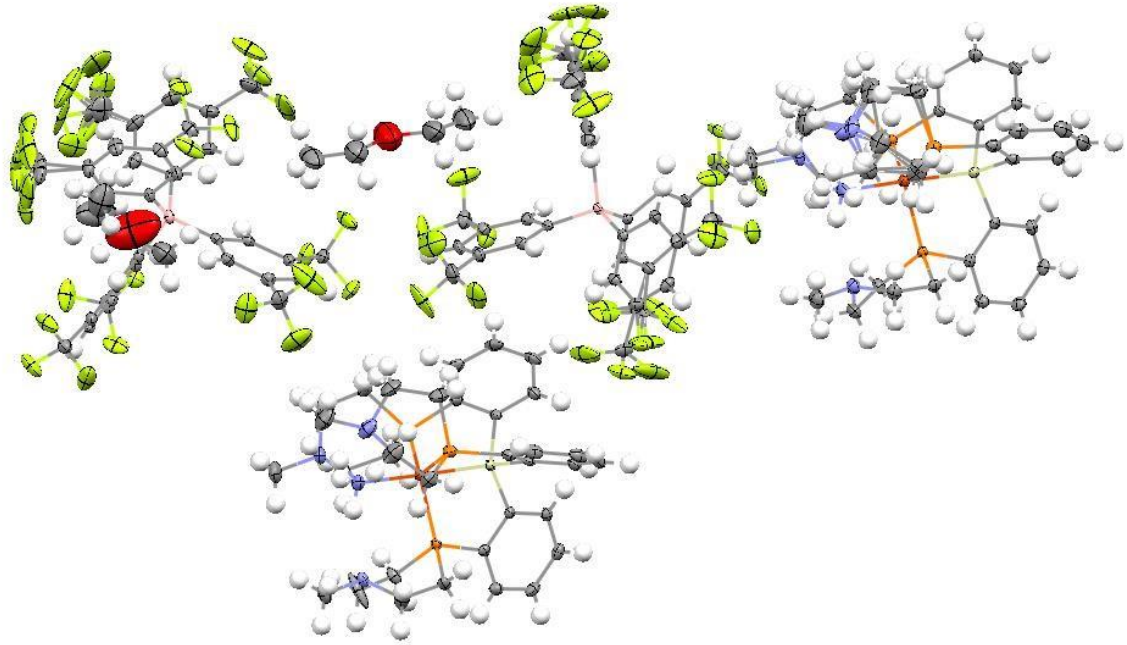

Figure S58. Full asymmetric unit of the structure of **3'**. Thermal ellipsoids shown at 50% probability.

[$SiP_2^{iPr}P^{NMe}$]$FeNH_2$ (5). Three disagreeable reflections were omitted from the refinement, and the data was refined as a racemic twin (BASF = 0.02031). The N-bound hydrogen atoms were located in the density difference map and restrained using DFIX to an N-H bond distance of about 1.0 Å, but otherwise were refined freely. One ligand isopropyl group was modeled as disordered in a 68:32 ratio.

{[$SiP_2^{iPr}P^{NMe}$]FeN_2}{$Na(THF)_2$} (6). This compound crystallizes as an infinite chain with sodium atoms bridging between molecules. One molecule is located in each asymmetric unit. One

disagreeable reflection (blocked by the beamstop) was omitted from the reflection. The data was refined as a twin with twin law [-1 0 0 0 -1 0 0 0 1 2] and BASF = 0.14269.

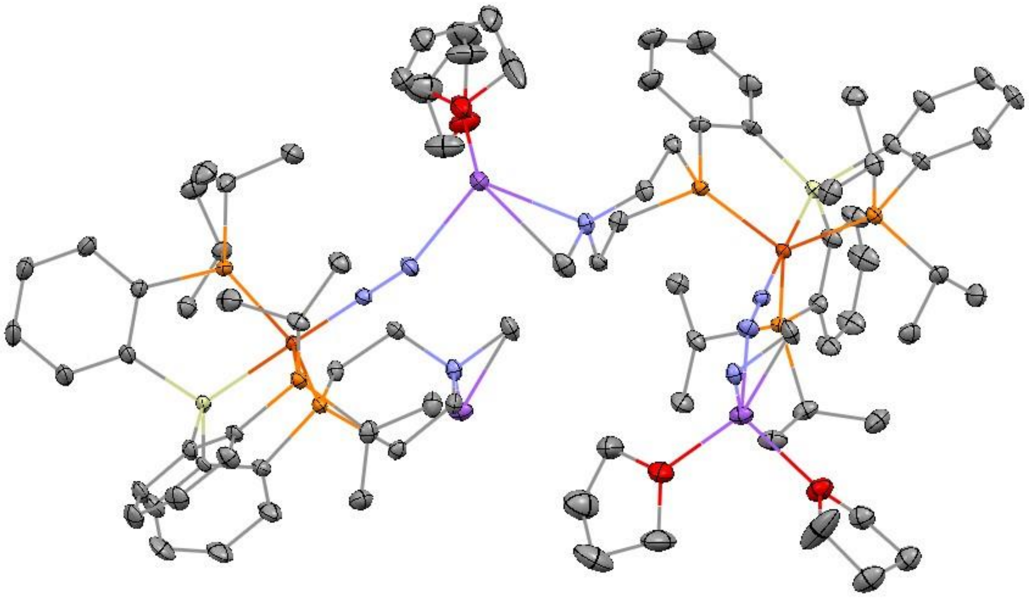

Figure S59. Structure of **6** showing two molecular units which make up part of an infinite chain. Thermal ellipsoids shown at 50% probability and hydrogen atoms omitted for clarity.

{[SiP$_3^{NMe}$]FeN$_2$}{Na(THF)$_3$} (6'). This crystal appears to undergo a phase change from an orthorhombic to a monoclinic crystal system upon cooling. Thus, data collected at 100 K suffered from twinning and the refined structure (monoclinic P2_1/c) has several large (1-3 e$^-$) residual electron density peaks near the metal center. In contrast, data collected at 200 K was successfully refined as orthorhombic (P$_{nma}$). In the 200 K structure, a mirror plane coincides with the Fe-P-NNa plane. One THF molecule is disordered across this plane. One other THF molecule was also modeled as disordered in a 59:41 ratio. The 100 K structure was refined as a twin (twin law: 1 0 0 0 -1 0 0 0 -1, BASF 0.37).

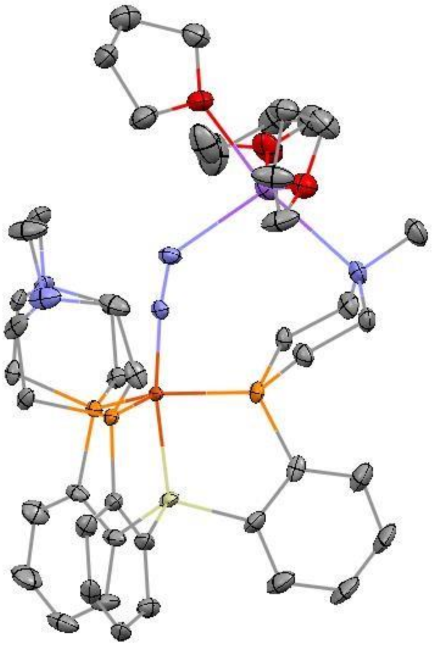

Figure S60. Full structure of **6'** at 100 K, thermal ellipsoids shown at 50% probability and hydrogen atoms omitted.

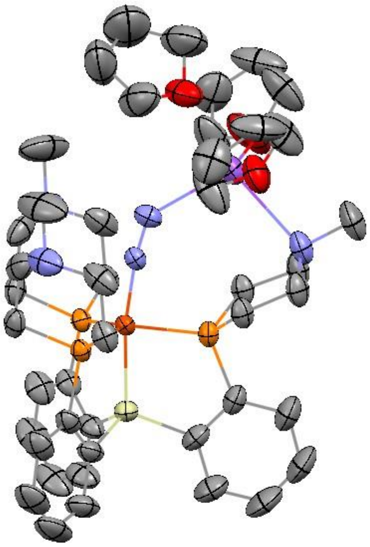

Figure S61. Structure of **6'** at 200 K, thermal ellipsoids shown at 50% probability and hydrogen atoms omitted.

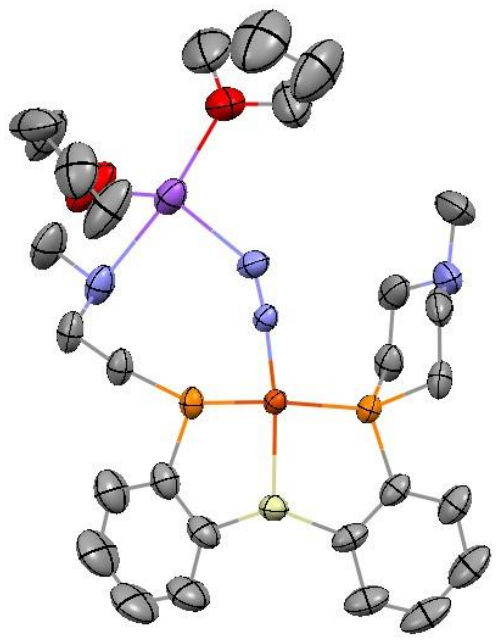

Figure S62. Asymmetric unit of the structure of **6** at 200 K.

[SiP^{iPr}$_2$P^{NMe}]Fe(N$_2$)(H). This species crystallizes with two molecules per asymmetric unit. Five disagreeable reflections (affected by the beamstop) were omitted from the refinement. The two metal-bound hydrogen atoms were located in the difference density map and refined freely. One phosphorus atom was refined as disordered over two positions in a 92:8 ratio, which may reflect a small population of a different isomer or compound.

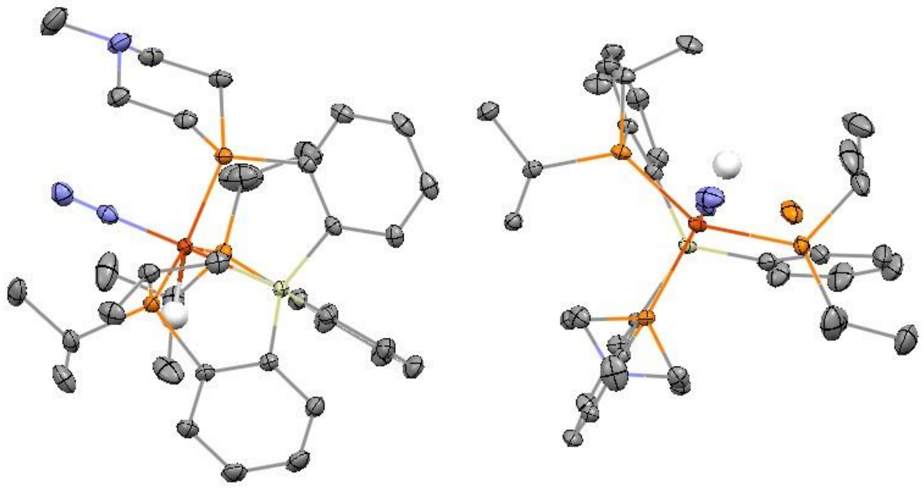

Figure S63. Structure of $[SiP^{iPr}{}_2P^{NMe}]Fe(N_2)(H)$ (two different views); ellipsoids shown at 50%; hydrogen atoms and disordered components omitted for clarity.

Compound	**1’**	**2’**	**4**
Identification code	a14104_final	a14107_final	a14133_final
Empirical formula	$C_{78}H_{94}Cl_2Fe_2N_6P_6Si_2$	$C_{33}H_{45}ClFeN_5P_3Si$	$C_{73}H_{82}BF_{24}FeN_3O_{1.5}P_3Si$
Formula weight	1540.23	688.59	1669.07
Temperature/K	100(2)	100(2)	100(2)
Crystal system	orthorhombic	monoclinic	triclinic
Space group	Pna21	P21	P-1
a/Å	38.962(2)	10.6457(5)	12.6269(13)
b/Å	11.0406(6)	19.2443(9)	16.1557(17)
c/Å	18.1487(9)	16.2208(8)	20.397(2)
α/°	90	90	86.951(5)
β/°	90	91.270(3)	77.672(5)
γ/°	90	90	72.605(5)
Volume/Å3	7807.0(7)	3322.3(3)	3878.7(7)
Z	4	4	2
ρ_{calc} g/cm3	1.348	1.377	1.429
μ/mm-1	0.640	0.666	0.375
F(000)	3224.0	1452.0	1718.0
Radiation	MoKα (λ = 0.71073)	MoKα (λ = 0.71073)	MoKα (λ = 0.71073)
2Θ range for data collection/°	3.066 to 62.012	2.512 to 56.686	2.642 to 66.56
Index ranges	-56 ≤ h ≤ 41, -15 ≤ k ≤ 15, -26 ≤ l ≤ 26	-14 ≤ h ≤ 14, -25 ≤ k ≤ 25, -21 ≤ l ≤ 21	-19 ≤ h ≤ 19, -24 ≤ k ≤ 24, -31 ≤ l ≤ 31
Reflections collected	150704	126704	247472
Independent reflections	24608 [R_{int} = 0.0476, R_{sigma} = 0.0370]	16433 [R_{int} = 0.0531, R_{sigma} = 0.0342]	29765 [R_{int} = 0.0480, R_{sigma} = 0.0287]
Data/restraints/parameters	24608/1/866	16433/1/776	29765/1087/1041
Goodness-of-fit on F2	1.158	1.100	1.021
Final R indexes [I>=2σ (I)]	R_1 = 0.0481, wR_2 = 0.1089	R_1 = 0.0461, wR_2 = 0.1121	R_1 = 0.0552, wR_2 = 0.1576
Final R indexes [all data]	R_1 = 0.0532, wR_2 = 0.1108	R_1 = 0.0501, wR_2 = 0.1145	R_1 = 0.0743, wR_2 = 0.1750
Largest diff. peak/hole / e Å-3	0.74/-0.54	1.66/-0.71	1.98/-1.33
Flack parameter	0.147(14)	0.027(16)	--

Table S1. Crystallographic data for the structures of **1’**, **2’**, and **4.**

Compound	**3’**	**6**	**6’ (200 K)**
Identification code	a14143_final	a14450_final	a15176_a
Empirical formula	$C_{69}H_{70}BF_{24}FeN_4OP_3Si$	$C_{43}H_{65}FeN_3NaO_2P_3Si$	$C_{45}H_{69}FeN_5NaO_3P_3Si$
Formula weight	1614.95	855.82	927.89
Temperature/K	100(2)	100(2)	200(2)
Crystal system	triclinic	monoclinic	orthorhombic
Space group	P-1	P21/c	Pnma
a/Å	17.5105(12)	13.454(5)	24.918(6)
b/Å	20.6792(14)	10.208(4)	16.256(4)
c/Å	22.9097(16)	32.264(14)	12.349(3)
α/°	102.798(3)	90	90
β/°	94.824(3)	90.860(8)	90
γ/°	111.770(3)	90	90
Volume/Å3	7385.0(9)	4431(3)	5002(2)
Z	4	4	4
$\rho_{calc}g/cm^3$	1.453	1.283	1.232
μ/mm-1	0.391	0.524	0.472
F(000)	3304.0	1824.0	1976.0
Radiation	MoKα (λ = 0.71073)	MoKα (λ = 0.71073)	MoKα (λ = 0.71073)
2Θ range for data collection/°	3.128 to 56.86	3.262 to 75.85	3.268 to 62.642
Index ranges	-23 ≤ h ≤ 23, -27 ≤ k ≤ 27, -30 ≤ l ≤ 30	-23 ≤ h ≤ 22, -17 ≤ k ≤ 17, -54 ≤ l ≤ 55	-34 ≤ h ≤ 36, -23 ≤ k ≤ 23, -17 ≤ l ≤ 16
Reflections collected	227665	108180	92587
Independent reflections	37050 [R_{int} = 0.0751, R_{sigma} = 0.0544]	23557 [R_{int} = 0.0795, R_{sigma} = 0.0880]	8081 [R_{int} = 0.0837, R_{sigma} = 0.0509]
Data/restraints/parameters	37050/2604/2118	23557/0/496	8081/521/356
Goodness-of-fit on F^2	1.014	1.055	1.063
Final R indexes [I>=2σ (I)]	R_1 = 0.0549, wR_2 = 0.1287	R_1 = 0.0860, wR_2 = 0.1956	R_1 = 0.0639, wR_2 = 0.1529
Final R indexes [all data]	R_1 = 0.0929, wR_2 = 0.1496	R1 = 0.1237, wR2 = 0.2116	R_1 = 0.1081, wR_2 = 0.1717
Largest diff. peak/hole / e Å-3	1.50/-1.13	1.42/-1.49	0.66/-0.29
Flack parameter	--	--	--

Table S2. Crystallographic data for the structures of **3’,** and **6**, and **6’** at 200 K**.**

Compound	**5**	**3**
Identification code	p14062_final	p14067_final
Empirical formula	$C_{35}H_{53}FeN_2P_3Si$	$C_{69}H_{71}BF_{24}FeN_2O_{0.5}P_3Si$
Formula weight	678.64	1579.93
Temperature/K	100(2)	100(2)
Crystal system	orthorhombic	triclinic
Space group	Pca21	P-1
a/Å	17.8087(5)	13.2134(6)
b/Å	11.3207(3)	16.5453(8)
c/Å	17.3127(5)	19.1158(9)
α/°	90	107.944(3)
β/°	90	103.367(3)
γ/°	90	104.913(3)
Volume/Å3	3490.36(17)	3616.2(3)
Z	4	2
ρ_{calc}g/cm3	1.291	1.451
μ/mm-1	0.631	3.393
F(000)	1448.0	1618.0
Radiation	MoKα (λ = 0.71073)	CuKα (λ = 1.54178)
2Θ range for data collection/°	5.82 to 72.676	5.162 to 150.52
Index ranges	-29 ≤ h ≤ 29, -18 ≤ k ≤ 18, -28 ≤ l ≤ 28	-16 ≤ h ≤ 16, -20 ≤ k ≤ 20, -23 ≤ l ≤ 23
Reflections collected	101085	134383
Independent reflections	16906 [R_{int} = 0.0536, R_{sigma} = 0.0453]	14802 [R_{int} = 0.1344, R_{sigma} = 0.0621]
Data/restraints/parameters	16906/3/425	14802/2398/1078
Goodness-of-fit on F2	1.034	1.014
Final R indexes [I>=2σ (I)]	R_1 = 0.0393, wR_2 = 0.0784	R_1 = 0.0690, wR_2 = 0.1612
Final R indexes [all data]	R_1 = 0.0571, wR_2 = 0.0845	R_1 = 0.1025, wR_2 = 0.1811
Largest diff. peak/hole / e Å-3	0.79/-0.54	0.70/-0.61
Flack parameter	0.020(11)	--

Table S3. Crystallographic data for the structures of **5** and **3.**

Compound	**SiP$_{iPr2}$P$_{NMe}$Fe(N$_2$)(H)**	**6' (100 K)**
Identification code	p15558_2	a14130_m_a
Empirical formula	$C_{70}H_{106}Fe_2N_6P_6Si_2$	$C_{45}H_{69}N_5O_3NaSiP_3Fe$
Formula weight	1385.30	927.89
Temperature/K	100(2)	99.99
Crystal system	triclinic	monoclinic
Space group	P-1	P21/c
a/Å	10.4778(10)	16.278(3)
b/Å	17.2329(17)	12.177(2)
c/Å	20.001(2)	24.314(4)
α/°	92.885(4)	90
β/°	96.497(3)	90.733(5)
γ/°	98.431(3)	90
Volume/Å3	3541.2(6)	4819.0(14)
Z	2	4
ρ_{calc}g/cm3	1.299	1.279
μ/mm-1	0.624	0.490
F(000)	1476.0	1976.0
Radiation	MoKα (λ = 0.71073)	MoKα (λ = 0.71073)
2Θ range for data collection/°	4.896 to 61.17	1.676 to 50.052
Index ranges	-14 ≤ h ≤ 14, -24 ≤ k ≤ 24, -28 ≤ l ≤ 28	-19 ≤ h ≤ 19, -14 ≤ k ≤ 14, -28 ≤ l ≤ 28
Reflections collected	301134	40561
Independent reflections	21650 [R_{int} = 0.0487, R_{sigma} = 0.0222]	8216 [R_{int} = 0.0638, R_{sigma} = 0.0707]
Data/restraints/parameters	21650/831/809	8216/1166/533
Goodness-of-fit on F2	1.021	1.173
Final R indexes [I>=2σ (I)]	R1 = 0.0358, wR2 = 0.0832	R1 = 0.0879, wR2 = 0.2229
Final R indexes [all data]	R1 = 0.0458, wR2 = 0.0883	R1 = 0.0996, wR2 = 0.2294
Largest diff. peak/hole / e Å-3	1.79/-0.98	1.78/-0.64

Table S4. Crystallographic data for the structures of SiP$^{iPr}_2$P^{NMe}Fe(N$_2$)(H) and **6'** at 100K.

VII. References.

[1] I. Bonnaventure and A. B. Charette, *J. Org. Chem.*, 2008, **73**, 6330.

[2] I. Chavez, A. Alvarez-Carena, E. Molins, A. Roig, W. Maniukiewicz, A. Arancibia, V. Arancibia, H. Brand and J. M. Manriquez, *J. Organomet. Chem.*, 2000, **601**, 126.

[3] M. Brookhart, B. Grant and A. F. Volpe, Jr., *Organometallics*, 1992, **11**, 3920.

[4] I. S. Weitz and M. Rabinovitz, *J. Chem. Soc., Perkin Trans. 1*, 19993, 117.

[5] A. Takaoka, N. P. Mankad and J. C. Peters, *J. Am. Chem. Soc.*, 2011, **133,** 8440.

[6] Y. Lee, N. P. Mankad and J. C. Peters, *Nat. Chem.*, 2010, **2**, 558.

[7] (a) D. F. Evans, *J. Chem. Soc.*, 1959, 2003; (b) E. M. Schubert, *J. Chem. Educ.*, 1992, **69**, 62.

[8] (a) M. W. Weatherburn, *Anal. Chem.*, 2003, **125**, 971; (b) J. S. Anderson, J. Rittle and J. C. Peters, *Nature*, 2013, **501**, 84.

[9] M. J. Frisch, G. W. Trucks, H. B. Schlegel, G. E. Scuseria, M. A. Robb, J. R. Cheeseman, G. Scalmani, V. Barone, B. Mennucci, G. A. Petersson, H. Nakatsuji, M. Caricato, X. Li, H. P. Hratchian, A. F. Izmaylov, J. Bloino, G. Zheng, J. L. Sonnenberg, M. Hada, M. Ehara, K. Toyota, R. Fukuda, J. Hasegawa, M. Ishida, T. Nakajima, Y. Honda, O. Kitao, H. Nakai, T. Vreven, J. A. Montgomery Jr, J. E. Peralta, F. Ogliaro, M. J. Bearpark, J. Heyd, E. N. Brothers, K. N. Kudin, V. N. Staroverov, R. Kobayashi, J. Normand, K. Raghavachari, A. P. Rendell, J. C. Burant, S. S. Iyengar, J. Tomasi, M. Cossi, N. Rega, N. J. Millam, M. Klene, J. E. Knox, J. B. Cross, V. Bakken, C. Adamo, J. Jaramillo, R. Gomperts, R. E. Stratmann, O. Yazyev, A. J. Austin, R. Cammi, C. Pomelli, J. W. Ochterski, R. L. Martin, K. Morokuma, V. G. Zakrzewski, G. A. Voth, P. Salvador, J. J. Dannenberg, S. Dapprich, A. D. Daniels, Ö. Farkas, J. B. Foresman, J. V. Ortiz, J. Cioslowski and D. J. Fox, Gaussian, Inc., Wallingford, CT, USA, 2009

[10] G. M. Sheldrick, *Acta Cryst. A*, 2008, **A64**, 112.
